# Intrinsic plasmon canalization in the biaxial van der Waals crystal MoOCl_2_

**DOI:** 10.1038/s41565-026-02243-9

**Published:** 2026-08-03

**Authors:** Farid Aghashirinov, Andrea Mancini, Lin Nan, Giacomo Venturi, Bettina Frank, Harald Giessen, Antonio Ambrosio

**Affiliations:** 1 4th Physics Institute and Research Center SCoPE, University of Stuttgart, Stuttgart, Germany; 2 Centre for Nano Science and Technology, Italian Institute of Technology, Milan, Italy

**Keywords:** Polaritons, Two-dimensional materials, Sub-wavelength optics

## Abstract

Anisotropic polaritons in low-symmetry crystals allow for subwavelength confinement and directional routing of light. The most extreme form of such anisotropy arises at the topological transition between elliptical and hyperbolic dispersion, where the isofrequency contours collapse into parallel lines and polaritons propagate in a diffractionless, beam-like fashion. This canalization regime has previously been accessed through twisted heterostructures or engineered metasurfaces. Here we show that intrinsic canalization can be achieved without any fabrication or structuring by exploiting the intrinsic elliptical-to-hyperbolic transition in the van der Waals crystal MoOCl_2_ at room temperature. Using near-field imaging, we directly visualize plasmon-polariton canalization emerging at the low-loss Drude crossing point along the [010] crystal axis. Owing to the moderate slope of the Drude permittivity, the resulting polaritons remain highly directional across a broad spectral window. This weak dispersion also enables robust thickness-dependent tuning, and we demonstrate, both experimentally and theoretically, that the canalization wavelength can be adjusted by more than 1 μm simply by varying the flake thickness. This work brings canalized polariton propagation into the 4.5–6 μm range, beyond the frequency limits of phonon–polariton platforms and overlapping with important molecular vibrations, opening new opportunities for mid-infrared nanophotonics and sensing.

## Abstract (web summary)

Near the Drude zero crossing where one in-plane permittivity component changes sign, the biaxial crystal MoOCl_2_ supports intrinsic diffraction-free canalized plasmon polaritons at wavelengths that are controlled by flake thickness.

## Main

Polaritons in van der Waals (vdW) materials provide extreme electromagnetic field confinement^1–4^, strong nonlinear responses^5,6^ and widely tunable dispersion^7,8^, offering a powerful platform for nanoscale control of optical energy^9–15^. In anisotropic crystals, polaritons can exhibit hyperbolic dispersion with open isofrequency contours (IFCs), enabling highly directional and deeply subdiffractional propagation^16–19^. Such behaviour has been extensively explored in vdW crystals supporting phonon polaritons, where lattice vibrations couple to electromagnetic fields to form strongly confined modes in the mid-infrared (IR)^20–22^. Recently, low-loss hyperbolicity has been extended to visible and near-IR frequencies via real-space imaging of plasmon–polariton (PP) propagation in the vdW crystal MoOCl_2_ (refs. ^23–27^), enabling access to natural hyperbolic polaritons in a spectral range that was previously inaccessible.

Among anisotropic dispersion phenomena, canalization is of particular interest as it occurs at the topological transition between hyperbolic and elliptical dispersions, where polaritons propagate without diffraction, forming highly directional, beam-like modes. Canalization has been realized through engineered in-plane anisotropy using metasurfaces^28–31^, twisted heterostructures^25,32–36^ and substrate engineering^37–41^. A conceptually simpler approach to achieve canalization is to exploit the intrinsic hyperbolic to elliptical transition occurring when one component of the permittivity changes sign. However, despite its simplicity, this effect has thus far been mainly explored in phonon–polariton platforms, where its observation is often hindered by the spectral structure of the dielectric response. In many cases, the transition frequency lies close to a phonon resonance, leading to increased damping and limiting the visibility of propagating canalized modes^42–47^, in addition to the intrinsically narrow bandwidth of Reststrahlen bands and, in some systems, the need for out-of-plane response^48^ or low-temperature operation^49^. So far, the only reported exception is the broadband plasmon canalization found in 2M-WS_2_, where the reduced steepness of its Drude dispersion enables canalized propagation over an extended wavelength range^50^. This observation points to a general materials strategy for broadband canalization based on plasmonic anisotropy rather than phononic resonances. However, proof of intrinsic canalization in 2M-WS_2_ relied on patterned structures to indirectly probe the polaritonic IFCs, precluding a direct, real-space visualization of canalized polariton propagation^50^.

Here, we demonstrate broadband PP canalization in MoOCl_2_ at mid-IR frequencies and at room temperature through direct near-field real-space mapping. We visualize the transition from hyperbolic to elliptical propagation across the 2.3–7 μm wavelength range and corroborate our observations with numerical simulations and analytical calculations of the IFCs. Furthermore, we theoretically predict that the canalization wavelength can be tuned over more than 1 μm by varying the flake thickness. This trend is experimentally confirmed through Fourier analysis of the near-field patterns, where the vanishing of the IFC curvature serves as a signature of the topological transition. In contrast to conventional phonon–polaritonic platforms governed by Lorentz-type dispersions, the Drude-like response of MoOCl_2_ enables broadband canalization over an extended spectral range while simultaneously providing efficient thickness-dependent tunability. The observed shift of the canalization wavelength is primarily driven by the dispersion along the [010] axis, whereas the strongly metallic response along [100] remains deeply negative and therefore only weakly influences the evolution of the IFC curvature with thickness.

## Intrinsic SPP canalization in MoOCl_2_

**Fig. 1:**
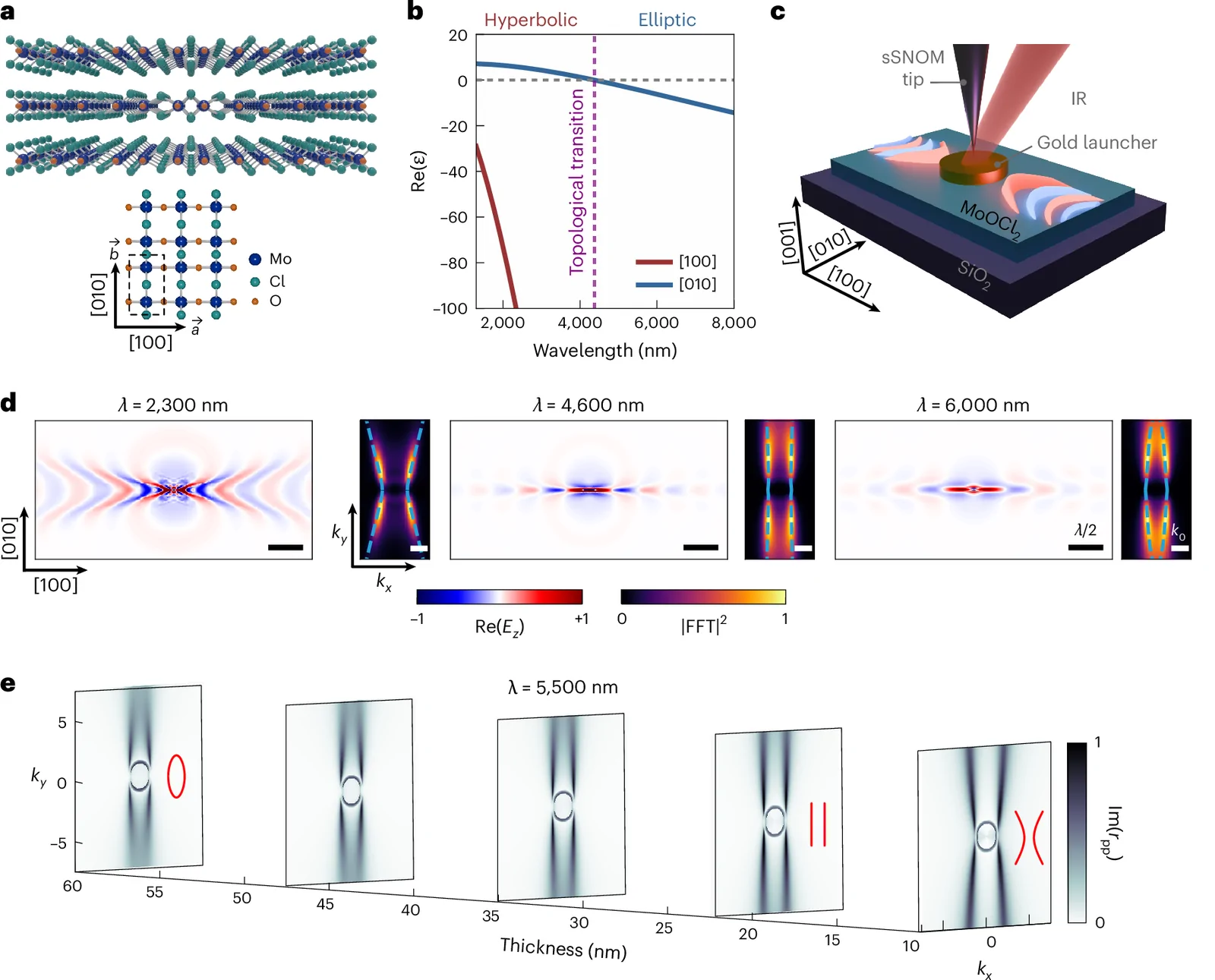
Intrinsic mid-IR PP canalization in hyperbolic MoOCl_2_. **a**, Crystal structure of bulk MoOCl_2_ and its in-plane view with the atomic 2D unit cell illustrated as dashed lines. **b**, Real part of the dielectric function of MoOCl_2_ along the *x* [100] and *y* [010] directions, showing the transition from the hyperbolic to elliptical regime. The point where ${\rm{Re}}(\varepsilon_{y} )=0$ corresponds to the topological transition frequency, at which canalization is expected. **c**, Schematic of the sSNOM measurement on a MoOCl_2_ flake where a gold disk nanoantenna is used to launch polaritons. **d**, Simulated near-field out of plane electric field maps of a dipole source placed on top of a MoOCl_2_ flake with a thickness of 45 nm and corresponding *k*-space images. Dashed lines are the analytically calculated in-plane plasmon IFCs. **e**, Transfer-matrix calculations at *λ* = 5,500 nm for different MoOCl_2_ thicknesses. *r*_pp_, *p*-polarized Fresnel coefficient.

Materials that intrinsically combine strong in-plane anisotropy with broadband plasmonic response are particularly promising candidates for realizing intrinsic PP canalization. Molybdenum oxychloride (MoOCl_2_) is a layered vdW material with a strongly anisotropic crystal and electronic structure^23,25,51–53^ (Fig. 1a), exhibiting metallic and dielectric responses along orthogonal in-plane directions from the visible to the mid-IR range. Due to an orbital-selective Peierls distortion, the in-plane permittivity components *ε*_*x*_ and *ε*_*y*_ have opposite signs over a broad spectral range (Fig. 1b), naturally supporting hyperbolic PPs^23,24^. The gradual Drude dispersion in MoOCl_2_ further produces a smooth transition between elliptical and hyperbolic regimes, enabling PP canalization without external symmetry breaking (Fig. 1b). The in-plane permittivity in Fig. 1b, which we use throughout our work, is experimentally extracted by fitting reflection and transmission spectra with a three layer model for various flake thicknesses^51^ (Methods; Supplementary Figs. 1–4).

We first confirm the presence of plasmon canalization around the transition frequency where the [010] permittivity changes sign via full three-dimensional electromagnetic simulations (Fig. 1d). We place a near-field source 50 nm above a 45-nm slab of MoOCl_2_ and record the out-of-plane electric field 5 nm above the surface. By sweeping the dipole emission wavelength from 2,300 nm to 6,000 nm, we can recognize all three propagation regimes: hyperbolic, canalized and elliptical. By two-dimensional (2D) fast Fourier transform (FFT) of the complex field, we obtain the IFC shape, which confirms the observation of the topological transition. The theoretical dispersion calculated in the small thickness limit^23,54^ (dashed blue lines) shows agreement of the model with the computed IFCs. We also observe that the shape of the IFCs around the topological transition can be adjusted by changing the MoOCl_2_ thickness, allowing for the engineering of the canalization frequency. We reveal this trend by computing the 2D IFCs via transfer-matrix formalism^55^ at a fixed wavelength (*λ* = 5,500 nm) for thicknesses ranging from 10 nm to 60 nm (Fig. 1e). We observe that thinner flakes show hyperbolic propagation, while thicker ones behave as elliptic, implying that for some intermediate value the IFCs must transition through a canalization point, which is here found around 22.5 nm. This analysis also explains why the canalization observed in the thin film case shown in Fig. 1d does not occur exactly at the bulk topological transition identified in Fig. 1b.

## Real-space imaging of canalized SPPs in MoOCl_2_ flakes

**Fig. 2:**
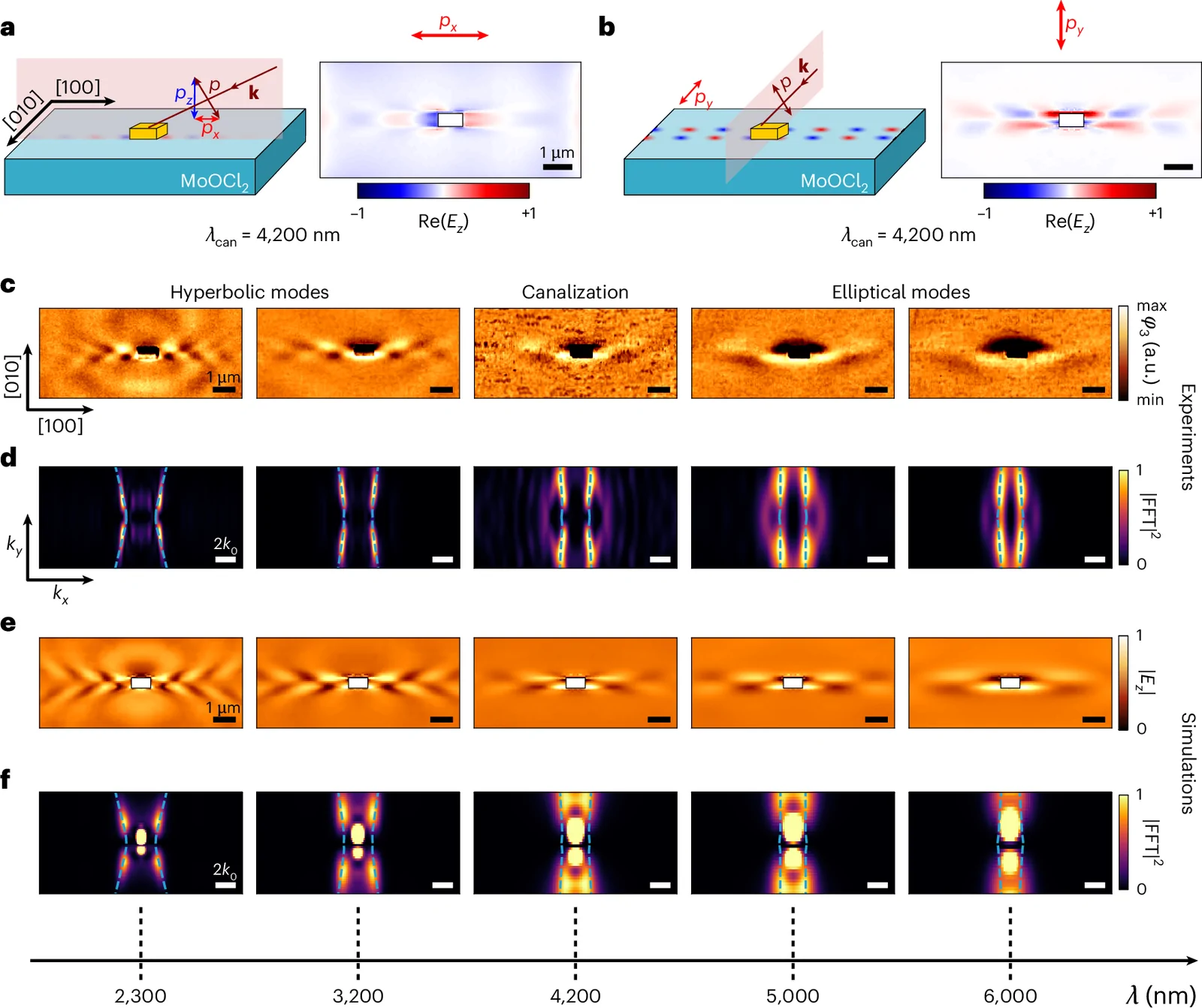
Real-space imaging of the intrinsic PP topological transition in MoOCl_2_. **a**,**b**, Schematic illustration and simulated near-field maps showing the polarization-dependent launching efficiency of a gold nanorod at the canalization wavelength. The red arrows illustrate the in-plane *p*_*x*_ and *p*_*y*_ polarization directions, so that the incident polarization is along the [100] direction (**a**) and along the [010] one (**b**). **c**,**d**, Experimental sSNOM phase maps (third demodulation order) of PPs launched by a gold nanorod fabricated on top of the flake (thickness 45 nm) at *λ*_0_ = 2,300, 3,200, 4,200, 5,000 and 6,000 nm (scale bars, 1 μm) (**c**) and corresponding *k*-space maps with analytical results of the PP dispersion (**d**). **e**,**f**, Simulated near-field maps of PPs launched by a gold nanorod fabricated on top of the flake (**e**), and the corresponding *k*-space maps (**f**).

To experimentally confirm the theoretical predictions presented in Fig. 1, we conduct extensive near-field measurements at various wavelengths on several flakes of different thicknesses. MoOCl_2_ flakes were obtained by mechanical exfoliation from commercially sourced bulk crystals and subsequently transferred onto 285-nm SiO_2_/Si substrates (Methods). Because MoOCl_2_ is strongly anisotropic, the in-plane polarization plays a crucial role in the excitation efficiency of PPs. In the experiments, the incident light is p-polarized to ensure efficient coupling to the scattering Scanning Near-field Optical Microscope (sSNOM) tip. When the in-plane electric field component *p*_*x*_ is aligned with the metallic crystallographic axis [100], the launching efficiency of the gold nanoantenna is strongly suppressed, as illustrated in Fig. 2a. Corresponding simulations at *λ*_can_ = 4,200 nm for a 45-nm-thick flake show rapidly decaying polaritons with only a few visible interference fringes. By contrast, aligning the in-plane polarization along the dielectric [010] axis enhances the launching efficiency, resulting in more extended interference patterns (Fig. 2b). For this reason, all subsequent measurements were performed with the in-plane polarization aligned along [010]. This behaviour can be qualitatively understood in terms of a better overlap between the angular distribution of the in-plane dipole induced in the launcher and the shape of the polariton IFCs at the canalization condition (see Supplementary Figs. 7–10 for more details and experimental verification).

The visual difference of the field profiles in Fig. 2a,b can be associated with a different instantaneous charge displacement direction with respect to the polaritons wavevector: parallel in Fig. 2a and orthogonal in Fig. 2b. An additional advantage of this arrangement is that no correction of the IFCs obtained from the experimental FFTs is required. This is because the light wavevector is nearly orthogonal to the polariton propagation direction, so the measured fringe periodicity directly corresponds to the polariton wavelength. By contrast, for collinear illumination geometries, the measured periodicity includes an additional contribution from the projection of the free-space wavevector, which depends on the illumination angle^56^. While this can be advantageous for accessing additional information by simultaneously observing both forward and backward-propagating polaritons with respect to the incident light momentum, it also introduces additional uncertainty in the quantitative extraction of the polariton wavevector due to the angle-dependent momentum contribution.

Near-field phase maps obtained on a 45-nm-thick flake at free-space wavelengths *λ*_0_ = 2,300, 3,200, 4,200, 5,000 and 6,000 nm are displayed in Fig. 2c. At *λ*_0_ = 2,300 nm and 3,200 nm, hyperbolic wavefronts are observed, originating from PPs launched by the gold nanorod, whereas at *λ*_0_ = 5,000 nm and 6,000 nm the wavefronts exhibit elliptical shapes. Between the hyperbolic and elliptic regimes, an intermediate propagation regime is expected in which waves propagate without divergence, commonly referred to as the canalization regime. According to Fig. 1b, for a flake thickness of 45 nm, this regime is predicted to occur at a wavelength of approximately 4,400 nm. However, this wavelength lies outside the accessible range of our laser source. Consequently, we employed the closest available wavelength that could be generated by our laser system to probe the canalization regime. This approach is justified because the dispersion relation shown in Fig. 1b varies slowly in this spectral region, and canalization does not occur at a single, sharply defined wavelength, but rather over a finite wavelength interval around the target value. For these reasons, we use a wavelength of 4,200 nm to excite the canalization mode. The transitions between the different propagation regimes can also be clearly observed in momentum space, as shown in Fig. 2d. By applying Fourier-transform analysis to the experimental near-field data, the resulting *k*-space images show open IFCs at *λ*_0_ = 2,300 nm and 3,200 nm, corresponding to the hyperbolic regime. At *λ*_0_ = 4,200 nm, the IFCs collapse into nearly parallel lines, characteristic of the canalization regime, whereas at *λ*_0_ = 5,000 nm and 6,000 nm the contours become closed, indicating the elliptic regime. Dashed blue lines are analytically calculated IFCs in the small thickness limit, showing excellent agreement of the analytical model and experimental FFTs. The observation of the polariton topological transition is further well confirmed by the simulated ∣*E*_*z*_∣ maps shown in Fig. 2e, as well as by the corresponding momentum-space (*k*-space) images presented in Fig. 2f together with analytical calculations (dashed blue lines).

## Canalization wavelength engineering via flake thickness

**Fig. 3:**
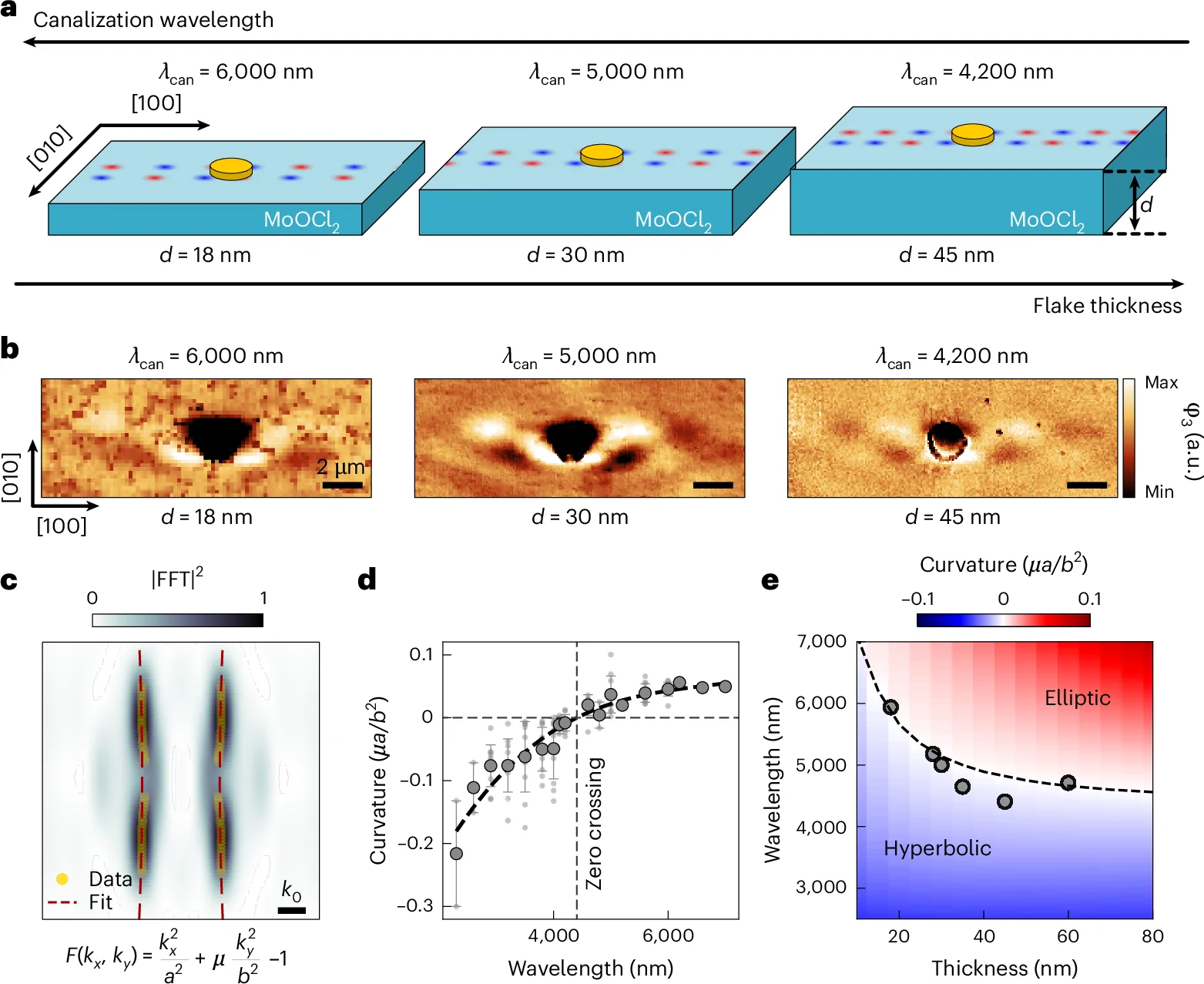
Tailoring canalization wavelength via flake thickness. **a**, Schematic illustrating the shift of the canalization wavelength towards lower values with increasing flake thickness. **b**, Real-space sSNOM phase maps (third demodulation order) showing canalized wavefronts launched by the gold disk nanoantenna for flakes of different thicknesses. **c**, Fourier transform of the canalized wavefront, fitted with a conic function (dashed red lines) to extract the curvature values. **d**, The curvature plot illustrates the transition of the supported modes from hyperbolic to canalized and subsequently to elliptical. Small grey dots represent curvature values extracted from the experimental data. Larger grey dots correspond to averaged values ± the standard deviation (*n* = 2 or *n* = 8 at each wavelength depending on the use of single of four-disk maps). The large grey dots are fitted with a polynomial curve to identify the zero-crossing position (vertical dashed line), which marks the expected canalization wavelength at approximately 4,400 nm. **e**, PP dispersion regimes with canalization window (white area) obtained from experiment (grey dots) and from simulations (dashed line). Error bars are obtained by propagating the uncertainty of the polynomial fit to the extracted curvature zero crossing. The blue and red regions correspond to hyperbolic and elliptic regimes, respectively.

As anticipated in Fig. 3e, the canalization wavelength can be controlled by tuning the flake thickness. Figure 3a schematically illustrates this effect. As the flake thickness increases, the wavelength at which canalization occurs shifts towards shorter values. To experimentally confirm this theoretical prediction, we carry out near-field experiments at frequencies both above and below the topological transition for several flakes of different thickness. In these experiments, gold disks are used as launchers instead of nanorods. The disks excite the same modes as the nanorods, as the launcher geometry for subwavelength scatterers does not appreciably alter the observed polariton pattern. Additional experimental data obtained using both gold nanorod and disk launchers are provided in Supplementary Figs. 11–15.

The experimental observations confirming the blueshift of the canalization wavelength with increasing thickness are shown in Fig. 3b. For a 18-nm-thick MoOCl_2_ flake, the canalization wavelength is observed at 6,000 nm. Increasing the thickness to 30 nm shifts the canalization wavelength to 5,000 nm, while for a thickness of 45 nm, canalization is observed at 4,200 nm. To quantitatively evaluate the shift of the canalization frequency, we fit the experimental *k*-space images with the conic function $f({k}_{x},{k}_{y})={k}_{x}^{2}/{a}^{2}+\mu {k}_{y}^{2}/{b}^{2}-1$, where *a* and *b* are free parameters, which can represent both elliptical (*μ* = 1) and hyperbolic (*μ* = −1) IFCs (see an example in Fig. 3c). To perform the fit, we have to reduce each image to a set of discrete *k*-space points: we perform this task by binarization of the FFT images, followed by detection of connected regions. From each of these regions, we extract a continuous line from the row-by-row weighted average of the data (for more details, see Supplementary Fig. 16). From the conic fit, we compute the vertex curvature at the *k*_*y*_ = 0 point as *κ* = *μa*/*b*^2^, which can be used as a clear signature of the topological transition, as *κ* = 0 represents straight line IFCs. From measurements taken on a wide range of wavelengths, we can produce a curvature versus wavelength curve (Fig. 3d), which we fit with a third-order polynomial to extract the zero-crossing point, representing our retrieved canalization wavelength (Supplementary Fig. 17). We carry out a similar procedure by using the same conic function to fit the analytically calculated IFCs for films with different thicknesses (see Supplementary Fig. 18 for more details). As a result, we obtain the image in Fig. 3e, from which we can trace the evolution of the zero crossing as highlighted by the dashed black line. The fact that the canalization frequency moves with the film thickness is not unexpected, as the shape of the IFCs for a hyperbolic film depend not only on the dielectric function, but also on the layer thickness. By looking at the equation for the theoretical IFCs (Supplementary Information section 6), additional insight into the thickness dependence of the canalization condition can be obtained. Assuming a frequency-independent dielectric response, with one in-plane permittivity component remaining deeply negative and the other negative but closer to zero, the canalization wavelength is expected to increase linearly with thickness. This follows from the analytical expression, where the slab thickness and free-space wavevector appear only through their product, so that the canalization condition is preserved by scaling thickness and wavelength by the same factor (Supplementary Fig. 20). In MoOCl_2_, however, the opposite trend is observed experimentally and theoretically: increasing the flake thickness leads to a reduction of the canalization wavelength. This behaviour is mainly driven by the dispersion of the [010] permittivity, which governs the evolution of the IFC shape across the topological transition. Because the permittivity enters the canalization condition multiplied by the flake thickness *d*, an increase in the absolute value of *ε*_*y*_ (that is, a more negative response) requires a smaller thickness to satisfy the condition. As a result, the thickness dependence of the canalization wavelength qualitatively follows the Drude-like dispersion of the [010] axis, explaining the trend reported in Fig. 3e. By contrast, the permittivity along the [100] direction remains strongly negative throughout the relevant spectral range and therefore behaves almost as a perfect electric conductor, contributing only weakly to the thickness-induced shift of the canalization condition.

From sSNOM experiments on flakes between 18 nm and 60 nm, we obtain the experimental data plotted as dots in Fig. 3e, confirming that the canalization wavelength can be tuned over more than 1 μm, corresponding to a variation >25% of the central wavelength by adjusting the flake thickness. We also show that, in a model Lorentz–Lorentz phonon–polariton material, canalization emerges only within a narrow spectral. As a consequence, the achievable thickness-dependent tuning remains intrinsically limited by the restricted bandwidth over which canalization exists (Supplementary Fig. 24). By contrast, the Drude-like response of MoOCl_2_ enables canalization across a much broader spectral range, allowing substantially larger shifts of the canalization wavelength with thickness. Importantly, such a high tuning bandwidth goes well beyond what has been achieved in phonon–polariton systems, where tuning via twist angle in bilayer^32,33^ and trilayer^35^ heterostructures or by substrate engineering^39,40^ was constrained to <9% of the central wavelength. Larger tunability has been reported for PhPs at terahertz frequencies^34^, yet it is still limited to less than 14% of the central energy.

## Conclusion

In summary, we have demonstrated that strongly directional, diffractionless PP propagation can arise in a low-symmetry vdW crystal by exploiting its intrinsic elliptical-to-hyperbolic topological transition, rather than relying on external patterning, twist engineering or artificial metastructures. This establishes a conceptually simple route to diffraction-free plasmon transport that is robust against fabrication imperfections and compatible with atomically thin, planar architectures.

An important implication of this work is that canalization in plasmonic systems does not need to be confined to a narrowly defined operating point. In MoOCl_2_, the gradual Drude dispersion enables a finite spectral window in which highly directional propagation persists, while thickness provides an efficient knob to position this window across the mid-IR. This combination of broadband response and tunability suggests new strategies for matching polariton propagation to external constraints, such as molecular vibrational bands, detector sensitivities or on-chip optical components.

Beyond MoOCl_2_, our findings motivate a broader search for intrinsic canalization in other anisotropic conductors and semimetals, particularly those exhibiting orbital-selective or symmetry-driven electronic anisotropy. Materials hosting multiple zero crossings or coexisting in-plane transitions could support cascaded or multichannel canalized propagation. Furthermore, integrating such crystals with electrostatic gating, phase-change substrates or strain fields may enable real-time steering and reconfiguration of polariton beams without lithographic modification.

## Methods

### Sample fabrication

MoOCl_2_ flakes were obtained by mechanical exfoliation from the corresponding bulk crystal (Nanjing MKNANO Tech.). Flakes investigated in the far-field experiments were deposited on SiO_2_, while for the near-field experiments we used Si/SiO_2_ (285 nm) substrates (Wafer University). Flakes with suitable ranges of thicknesses were selected for the launcher fabrication via the flake colour. The thicknesses were then confirmed by atomic force microscopy (AFM) measurements. The nanostructures were fabricated by a standard electron-beam lithography followed by metal deposition and lift-off. A layer of poly(methyl methacrylate) resist, PMMA 950K A4, was spin-coated onto the substrate at 4,000 rpm for 60 s and subsequently baked at 180 °C for 3 min. The resist was patterned using 50-kV electron-beam lithography with a 50-μm aperture. After exposure, the sample was developed for 60 s in a 1:3 mixture of methyl isobutyl ketone and isopropyl alcohol. The development was stopped by rinsing the sample in isopropyl alcohol, followed by drying under a nitrogen gas flow. A 2-nm chromium adhesion layer and a 45-nm gold layer were then deposited by electron-beam evaporation. Finally, lift-off was performed by immersing the sample in acetone for more than 3 h, leaving the patterned gold nanostructures on the flakes.

### Fourier-transform IR spectroscopy

Polarization-resolved reflectivity and transmission IR spectra were acquired on the sample using a Bruker Vertex 80 microscope Hyperion 3000 equipped with a 36× Cassegrain objective. For each crystallographic axis of MoOCl_2_, the reflectance and transmittance were measured under near-normal incidence for a number of flakes with different thicknesses. The in-plane dielectric function was extracted by fitting the measured spectra with a three-layer model (vacuum–MoOCl_2_–SiO_2_) analogously to what was performed in ref. ^51^. For simplicity, we ignored the off-angle illumination introduced by the Cassegrain objective. Transmission measurements were fitted only up to 5 μm because of the onset of absorption of the glass substrate. The final dielectric function was obtained by fitting the average dielectric function with two Drude–Lorentz models (one for each crystal axis). Before fitting, the thickness of each flake was fixed by fitting the optical response (reflection and transmission) in the visible along both axes with the dielectric function reported in ref. ^51^ (see also Supplementary Information section 1). The fitting was performed with the layer thickness as the only free parameter. IR spectra were acquired using a broadband light source in conjunction with a CaF_2_ near-IR beam splitter, covering the spectral range from 0.7 to 8.3 μm. Visible spectra were acquired with a custom-made microscope using a quartz tungsten-halogen Lamp (Thorlabs, QTH10) as the light source. Light was focused and collected by a pair of 20× long working distance objectives (Mitutoyo) and directed to a fibre-coupled spectrometer (Thorlabs, CCT10). Before collection, a 4*f* system and an iris were used to cut the re-imaged image plane to the area of the flake we intended to measure. A polarizer (Thorlabs, WP25M-UB1) in conjunction with a broadband half waveplate (Thorlabs, AQWP05M-580) were used to measure along the different axes of the crystal. By rotating the half waveplate and collecting angle-dependent reflectivity spectra, we determine the [100] and [010] axes as the ones with, respectively, the maximum and minimum integrated reflectance in the range 700–850 nm.

### sSNOM

Near-field maps presented in Fig. 2c were acquired using a commercial room-temperature sSNOM from Neaspec GmbH coupled to a broadband tunable laser source from Stuttgart Instruments GmbH covering 1,450–20,000 nm wavelength range. In the present experiments, the laser wavelength was tuned from 2,300 nm to 7,000 nm to selectively excite specific plasmon-polaritonic modes. The laser beam was focused by an off-axis parabolic mirror (incident angle ~60°) onto the apex of a metal-coated AFM tip (NCLPt, Pt/Ir coating). Light is p-polarized to maximize the projection along the tip’s vertical axis. The tip was operated in tapping mode, with the cantilever oscillating at 193 kHz, and the tapping amplitude was adjusted depending on the wavelength to ensure operation strictly in the near-field regime and to maximize the signal-to-noise ratio.

The sample, consisting of gold launchers (disks and nanorods) fabricated on a MoOCl_2_ flake, was raster-scanned beneath the AFM tip. The tip acts as a localized near-field source, providing the momentum required to launch PPs on the sample surface. These modes propagate across the MoOCl_2_ flake and are scattered by the gold launcher, or conversely, the launcher scatters modes launched by the tip. The resulting interference between light scattered from the tip and the launcher generates a modulated far-field signal at the detector. The amplitude and phase of this modulation depend sensitively on the tip–launcher distance, enabling high-resolution imaging of the spatial distribution and propagation characteristics of the PPs. To isolate the near-field contribution from background far-field scattering, the detector signal was demodulated at higher harmonics (*nΩ*, *n* = 3) of the cantilever oscillation frequency.

Before focusing on the sample, half of the laser beam was redirected to a pseudo-heterodyne interferometer via a beam splitter. Light scattered by the tip was then recombined with the interferometer reference, allowing retrieval of both amplitude- and phase-resolved maps of the electromagnetic field at the sample surface by properly setting the mirror oscillation amplitude. To maximize PP scattering efficiency, the MoOCl_2_ flake was rotated such that its dielectric axis was aligned with the laser incidence direction (Fig. 2b).

### Numerical simulations

Electromagnetic simulations were performed with a commercial solver (CST Studio) in the frequency domain. The dielectric function of MoOCl_2_ was extracted from the experimental optical spectra as detailed above. For the dipole simulations, we placed a near-field source above (50 nm) a MoOCl_2_ slab. The near-field source was obtained by inserting a discrete port in a vacuum gap inside a perfect electric conductor cylinder. As CST does not support open boundaries for anisotropic media, a vacuum gap was inserted between the end of the MoOCl_2_ slab and the open boundary conditions. Simulations of the PPs launched by disks and rods (modelled as perfect electric conductor solids) were carried out with a plane-wave sourced with a 60° tilt from the normal direction.

Calculations of the imaginary part of the reflection coefficients were performed using a 4 × 4 transfer matrix formalism^55^ for the homogenous vacuum–MoOCl_2_–SiO_2_ stack. The 2D maps were obtained by rotating the MoOCl_2_ dielectric function around the *z* axis and computing the reflection coefficients at each angle.

## Online content

Any methods, additional references, Nature Portfolio reporting summaries, source data, extended data, supplementary information, acknowledgements, peer review information; details of author contributions and competing interests; and statements of data and code availability are available at https://doi.org/10.1038/s41565-026-02243-9.

## Supplementary information

Supplementary Information Supplementary Notes 1–8, Figs. 1–31 and Tables 1 and 2.

Peer Review File

Supplementary Video 1 Transfer matrix calculation of the wavelength evolution of the IFCs for a 45 nm MoOCl_2_ film.

Supplementary Video 2 Near-field maps of polaritons launched by a gold rod and analytical calculations of the wavelength evolution of the IFCs for a 45-nm MoOCl_2_ film.

Supplementary Video 3 Phase evolution at the canalization wavelength for polaritons launched by a gold rod on a 45-nm MoOCl_2_ film. The video is reconstructed from the near-field complex data.

## Data Availability

The data that support the findings of this study are presented in the Article and its Supplementary Information. The associated raw data are available via Zenodo at https://doi.org/10.5281/zenodo.20816757 (ref. ^57^). Additional data are available from the authors upon reasonable request.
