# Supplementary material for: Intrinsic plasmon canalization in the biaxial van der Waals crystal MoOCl2
Source: Nat Nanotechnol. 2026 Aug 3;21(9):1238–44. doi: 10.1038/s41565-026-02243-9 (PMC13569449; doi:10.1038/s41565-026-02243-9)
Supplement: Supplementary file 1 [file 41565_2026_2243_MOESM1_ESM.pdf]

nature nanotechnology

Supplementary information https://doi.org/10.1038/s41565-026-02243-9

Intrinsic plasmon canalization in the biaxial van der Waals crystal $MoOCl_2$

In the format provided by the authors and unedited

Supplementary Information

Contents

1 Extraction of mid-IR in-plane dielectric function **2**

2 Role of the polarization for the efficient excitation of plasmon polaritons **8**

2.1 Simulations of launched polaritons under different polarization and rod orientation . . . 8

2.2 Understanding the advantage of using polarization aligned with the [010] direction . . . 10

2.3 Experimental polariton launching under different polarization excitation . . . 12

3 Additional sSNOM phase maps **13**

4 Extraction of experimental IFC curvature **16**

5 Experimental thickness-dependent canalization frequency **19**

6 Theoretical thickness-dependent canalization frequency **20**

6.1 Canalization wavelength shift in a Lorentz-Lorentz material . . 25

7 Propagation length of anisotropic plasmon polaritons **29**

8 Increasing canalization mode confinement **30**

1 Extraction of mid-IR in-plane dielectric function

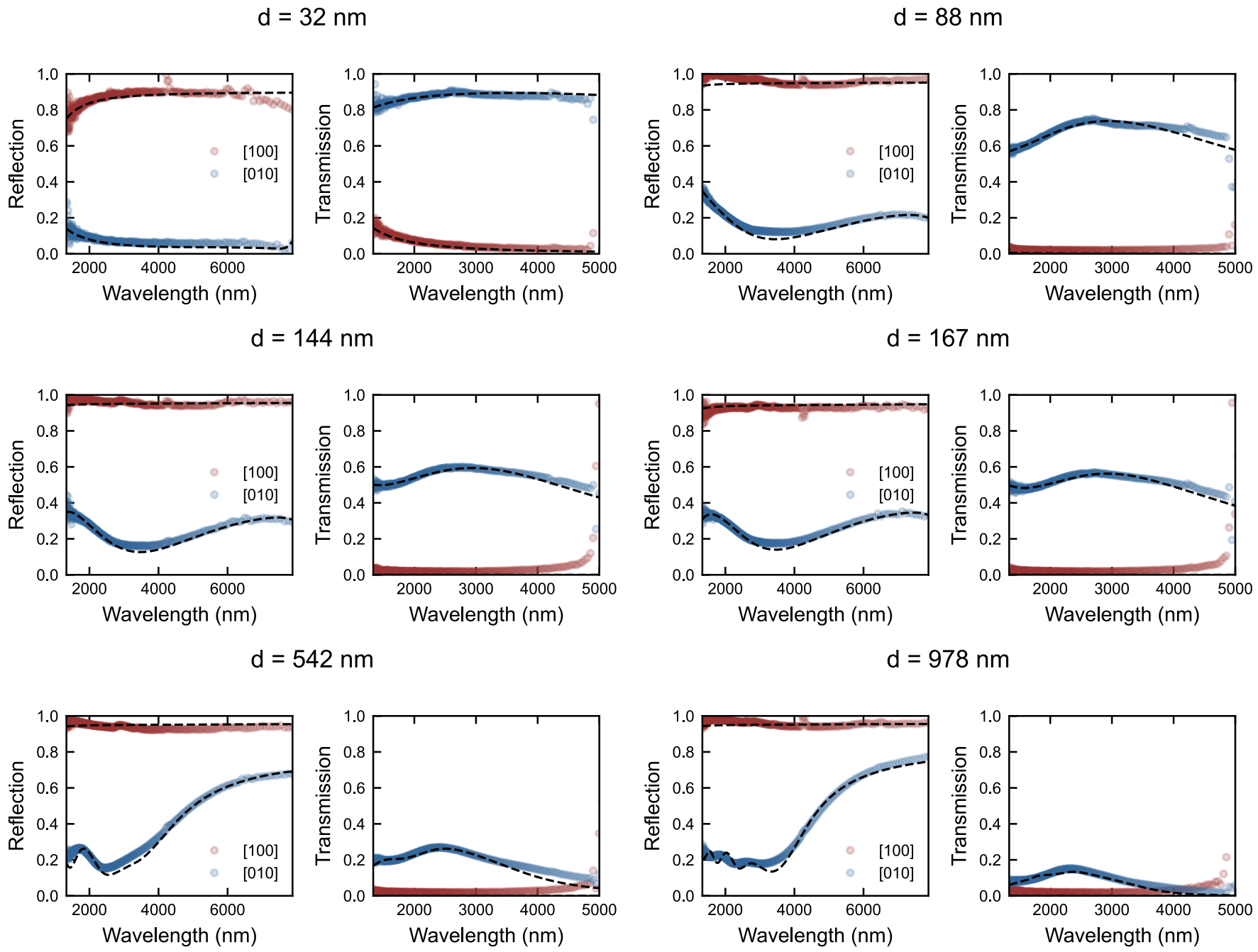

Fig. 1S: **Extraction of the in-plane dielectric function.** Fits of FTIR reflection and transmission spectra (dashed black lines) with a three layer model from which the in-plane dielectric function is extracted.

The in-plane dielectric function was extracted similarly to Ref. [1] by fitting experimental reflection and transmission spectra with a 3-layer model (vacuum-$MoOCl_2$-SiO_2). Polarization-resolved reflectivity and transmission infrared spectra acquired on several $MoOCl_2$ flakes taken with a FTIR microscope are shown in Fig. 1S. The two axes are readily identified by the minimum and maximum reflection points while rotating the polarizer (see also Methods section). For simplicity, we neglect here the tilted illumination provided by the Cassegrain objective and assume normal incidence. The three-layer system is modeled by the following equations for the reflection and transmission coefficients [2]:

$$r = \frac{r_{12} + r_{23}e^{2i\delta}}{1 + r_{12}r_{23}e^{2i\delta}} \tag{1}$$

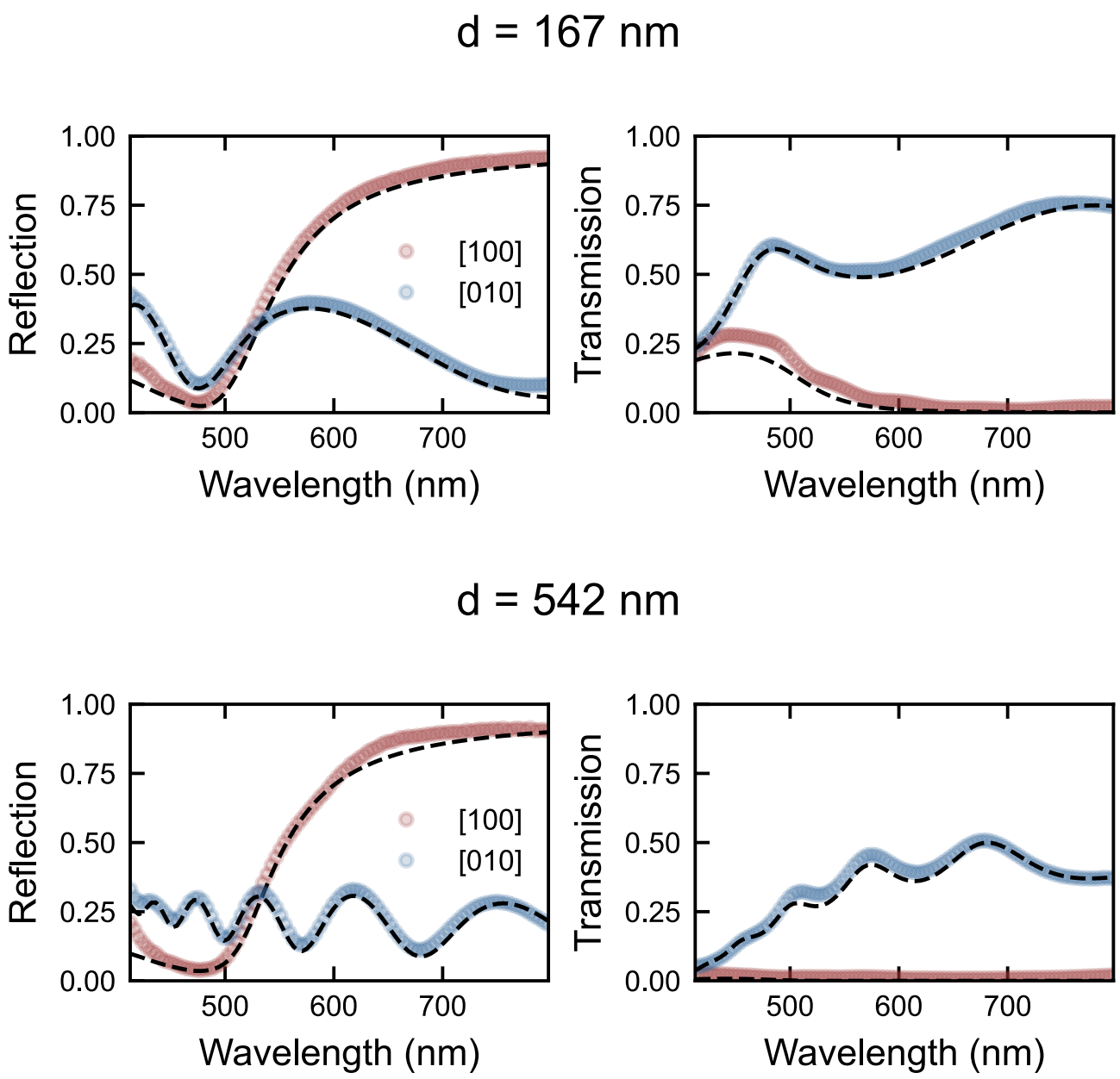

Fig. 2S: **Determination of flake thickness via visible spectroscopy.** Examples of the determination of flake thicknesses by fitting optical spectra in a range where the dielectric function is known [1].

$$t = \frac{t_{12}t_{23}e^{i\delta}}{1 + r_{12}r_{23}e^{2i\delta}} \tag{2}$$

where the r_{ij} and t_{ij} are Frensel coefficients of single interfaces:

$$\delta = 2\pi dn/\lambda$$
$$r_{ij} = \frac{n_i - n_j}{n_i + n_j}$$
$$t_{ij} = \frac{2n_j}{n_i + n_j}$$

Here d and n are the $MoOCl_2$ thickness and complex refractive index, respectively, and λ is the wavelength. The indexes correspond to 1 for the material above the flake (air, $n = 1$), 2 for the $MoOCl_2$ layer, and 3 for the SiO_2 substrate. The observed reflection and transmission are then calculated as $R = \mathsf{r}^2$ and $T = \mathsf{t}^2/n_3$. Each flake thickness is determined from optical spectra taken in the visible as detailed in Ref. [1], where the dielectric function is fixed and the thickness is left as the only fitting parameter (Fig. 2S).

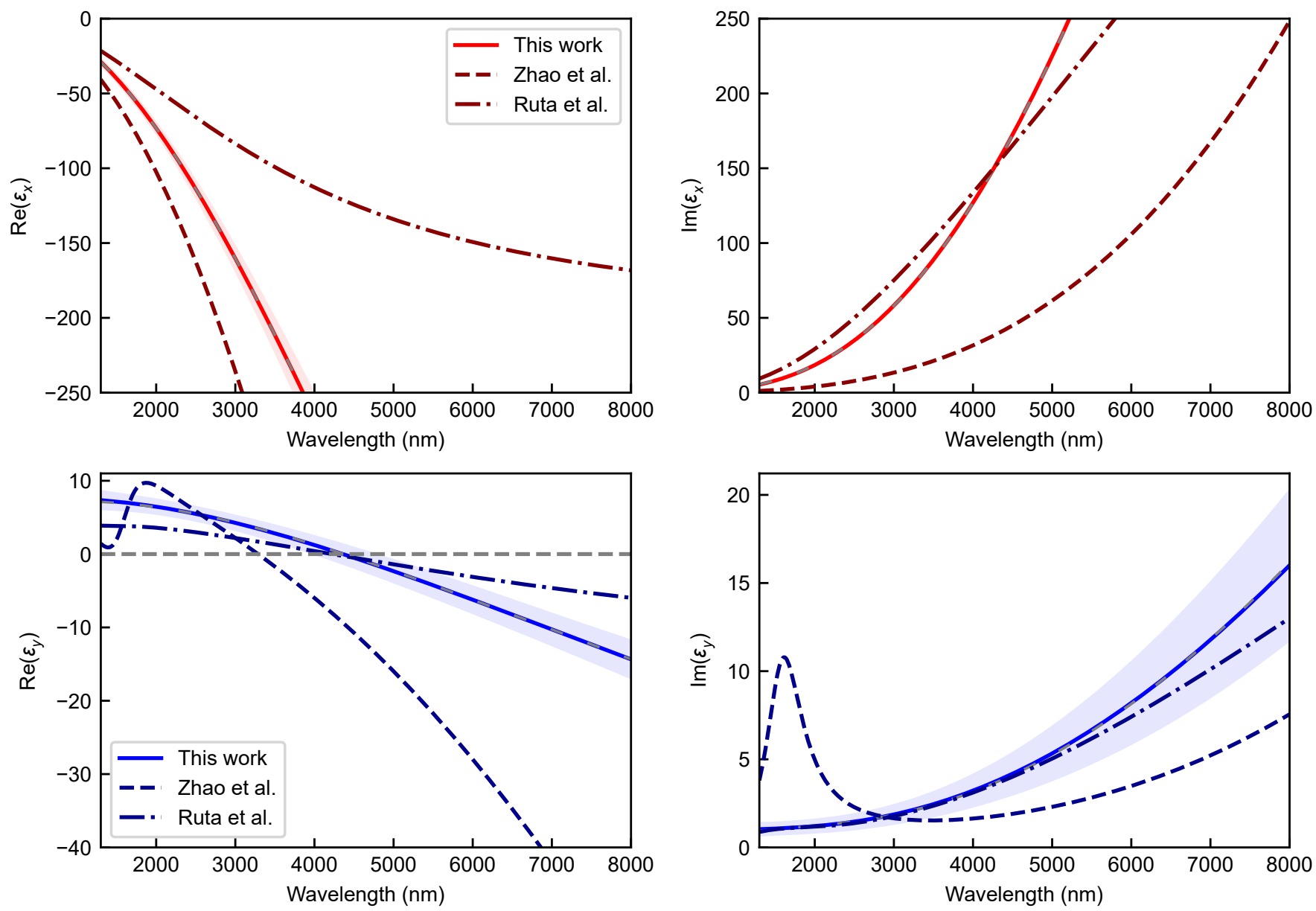

Fig. 3S: **Mid-IR dielectric function comparison.** Comparison of the extracted mid-IR complex dielectric function along both crystal directions with previously proposed models [3, 4].

For the extraction of the mid-IR parameters, we fit the dielectric function of both the $MoOCl_2$ [100] and [010] axes with a Lorentz-Drude model:

$$\varepsilon(\omega) = \varepsilon_\infty - \frac{\omega_p^2}{\omega(\omega + i\gamma)} + \frac{\omega_{p,j}^2}{\omega_{0,j}^2 - \omega^2 - i\gamma_j\omega} \tag{3}$$

The parameters in Eq.(3) are the ones used to minimize the fit to the experimental data. For a robust estimation of the dielectric function parameters, we fit reflection and transmission spectra along a single axis at the same time with the same Drude-Lorentz values. We do this by stitching the reflection and transmission experimental data, which we fit with the concatenation of the calculated spectra from the three layer system. The extracted Drude Lorentz parameters are reported in table 1, while a comparison of the dielectric function with the one calculated by DFT [3] and extracted via reflection experiment of a single thick flake [4] is shown in Fig. 3S.

The decomposition of the extracted dielectric function into its constituent contributions (Fig. 4S) highlights the distinct physical origins of the optical response along the two crystallographic directions. In the [100]-direction, the permittivity is well described by a single Drude term, indicating a predominantly free-carrier response with no significant interband contributions in the spectral range of interest. In contrast, the [010]-direction requires the inclusion

Axis	ε_∞	ω_p	γ	$\omega_{0,1}$	$\omega_{p,1}$	γ_1
[100]	0	5.63	0.15	0	0	0
[010]	7.85	0.91	0.11	1.15	1.30	1.99

Table 1: **Values of the parameters for the mid-IR dielectric function extracted from the fit**. All the values are reported in eV, except for ε_∞ which is a dimensionless parameter.

of an additional Lorentz oscillator to accurately capture the low transmission at lower wavelengths in the investigated range.

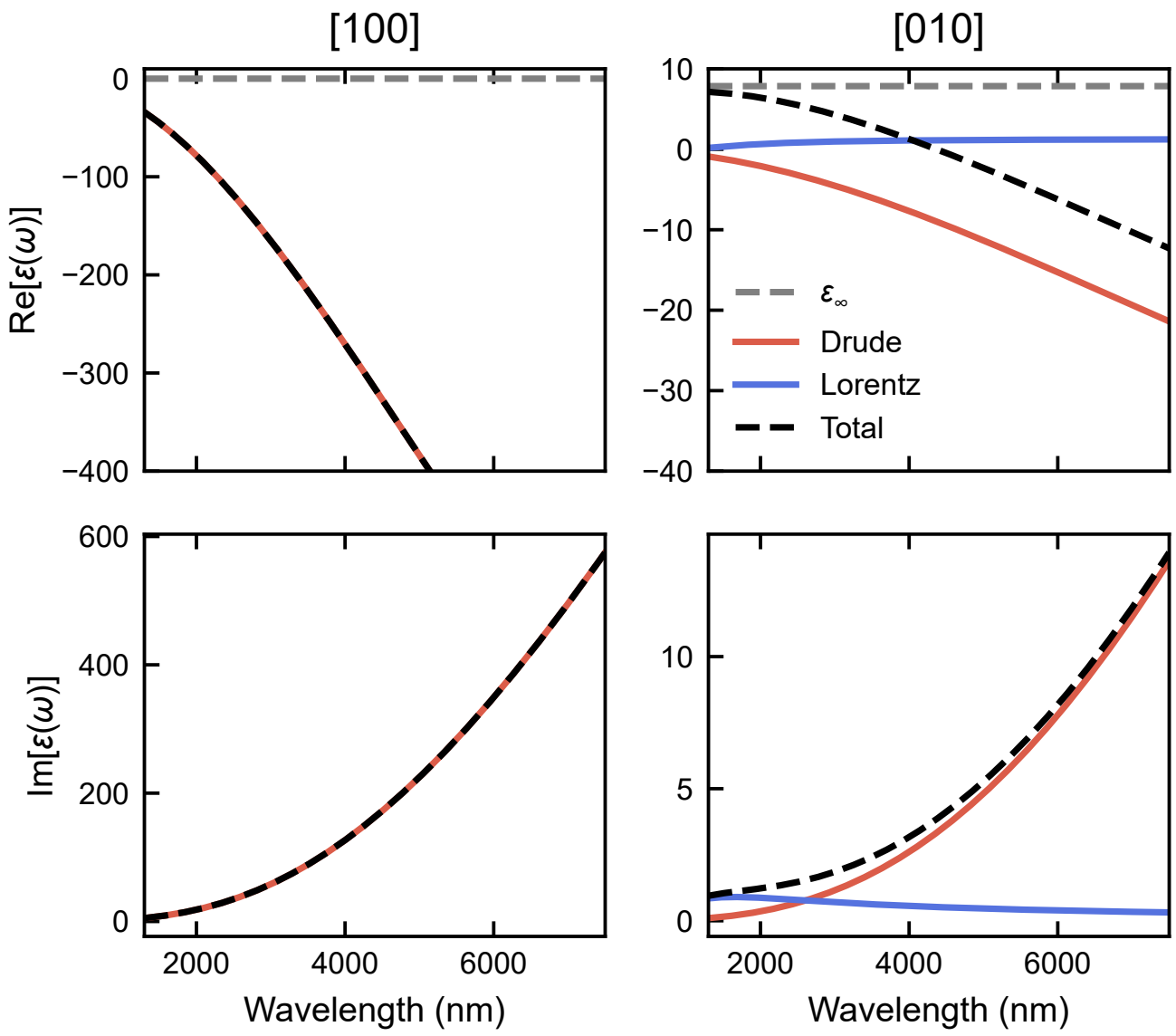

Fig. 4S: **Mid-IR dielectric function decomposition** Contribution from the Drude and Lorentz terms to the extracted dielectric function of $MoOCl_2$ in the IR range.

We also performed a sensitivity analysis of the dielectric function fitting. For the [100] direction, $\mathrm{Re}(\varepsilon_x)$ is strongly negative in the spectral range of interest, so the response is effectively metallic and the fit is well constrained by a single Drude term, leading to negligible impact on the extracted IFCs. In contrast, the [010] direction allows for greater flexibility, as different parameter combinations can reproduce the reflection and transmission data with comparable accuracy (Fig. 5S, Fit 1 corresponds to the one used in the manuscript with parameters reported above). In particular, variations in ε_∞ can be partially compensated by a redistribution between the Drude and Lorentz contributions. To assess this, we performed additional fits starting from different

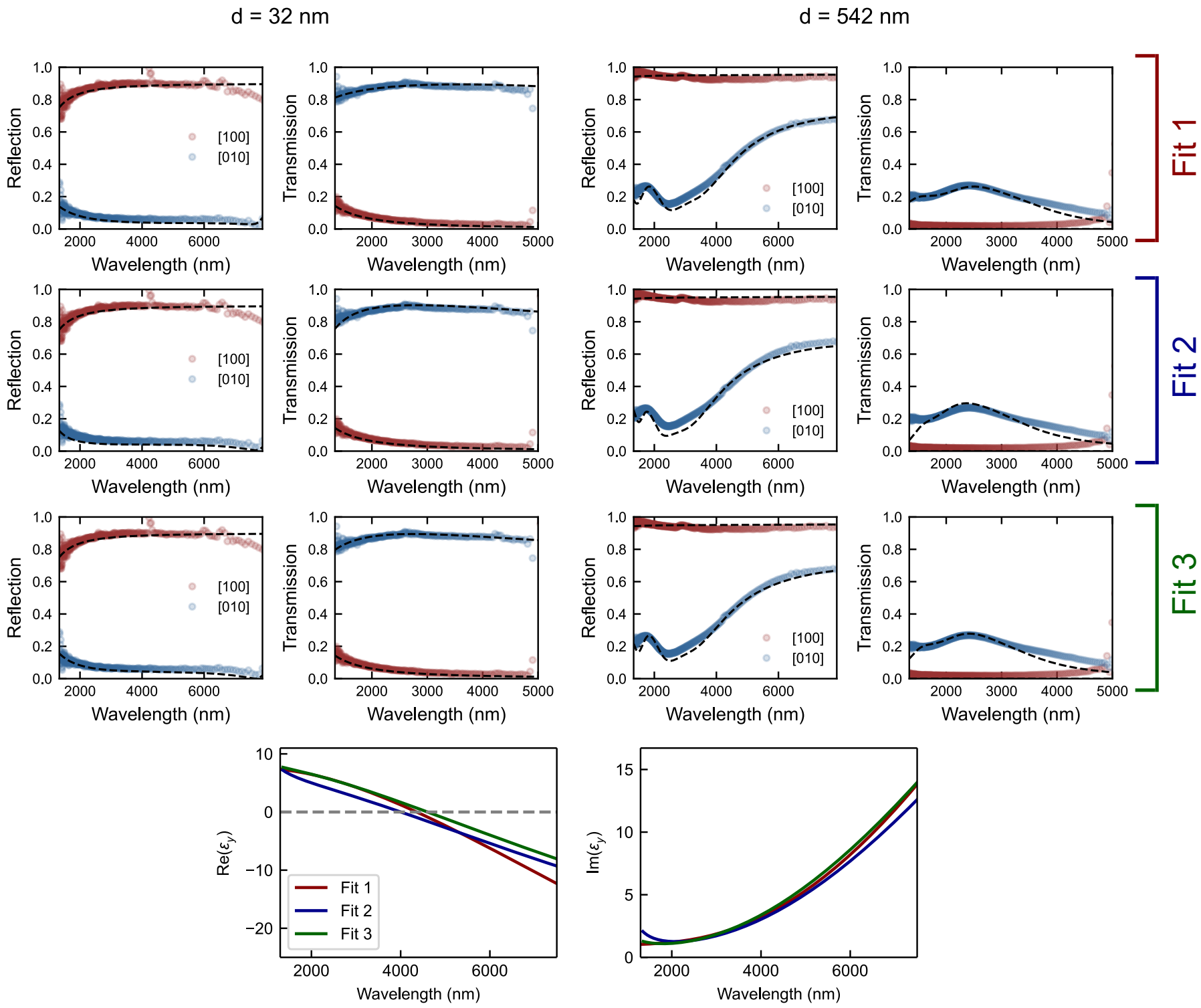

Fig. 5S: **Sensitivity of the [010] dielectric function fitting.** Reflection and transmission spectra along the [100] and [010] directions for two representative flake thicknesses (32 and 542 nm) are shown together with three fits obtained from different starting parameters. Bottom panels show the corresponding extracted real and imaginary parts (obtained from the average of all the flakes thicknesses as in Fig. 1S) of ε along the [010] axis, illustrating the variability arising from fitting degeneracy.

ε_∞ values and extracted the corresponding dielectric functions. The resulting variation is illustrated in the new figure, where representative fits and the corresponding extracted permittivities are shown for two flake thicknesses, with the associated fit parameters (for Fit #2 and #3) reported in Table 2.

The sensitivity of the polariton IFCs to the different dielectric-function fits is shown in Fig. 6S. For each fitted permittivity, we recalculated the IFCs, extracted the curvature using the same conic fitting procedure employed in the main text, and determined the curvature zero-crossing wavelength as a function of thickness. The calculated curvature maps and zero-crossing lines show that the qualitative behavior is robust across the different parameterizations: all fits reproduce a thickness-dependent redshift of the canalization wavelength with a similar trend of the hyperbolic-to-elliptical evolution of the IFCs. However, the absolute spectral position of the canalization curve depends

Fit # ([010])	ε_∞	ω_p	γ	$\omega_{0,1}$	$\omega_{p,1}$	γ_1
1	4.32	0.84	0.14	1.13	1.49	0.35
2	6.99	0.88	0.14	1.29	1.49	0.93

Table 2: Values of the parameters for the [010] mid-IR dielectric function extracted from the additional fits #2 and #3 in Fig. 5S. All the values are reported in eV, except for ε_∞ which is a dimensionless parameter.

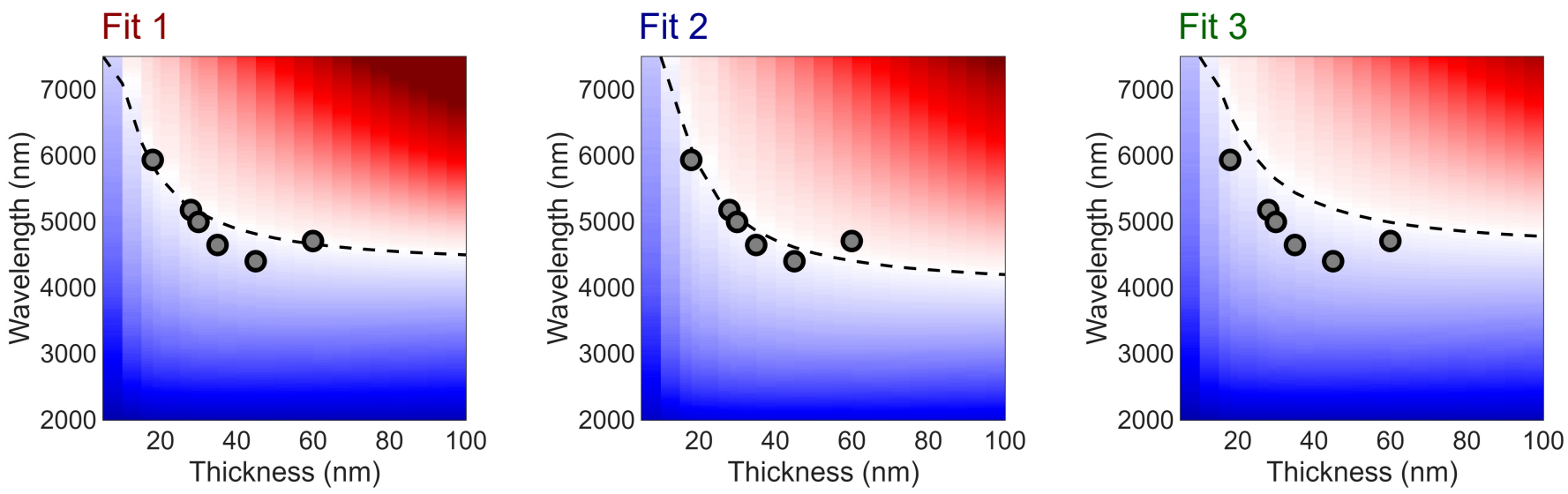

Fig. 6S: Comparison of expected shift of the canalization wavelength with flake thickness for different fitted [010] permittivity. IFCs curvature evolution as a function of wavelength and thickness for the different fits shown in Fig. 3S.

on the fitted $\varepsilon_y(\omega)$, with Fit 3 producing a more pronounced redshift. Thus, the uncertainty in the dielectric-function fitting affects the precise calculated canalization wavelength, but not the main conclusion that intrinsic canalization persists and remains thickness-tunable over the experimentally observed range.

2 Role of the polarization for the efficient excitation of plasmon polaritons

2.1 Simulations of launched polaritons under different polarization and rod orientation

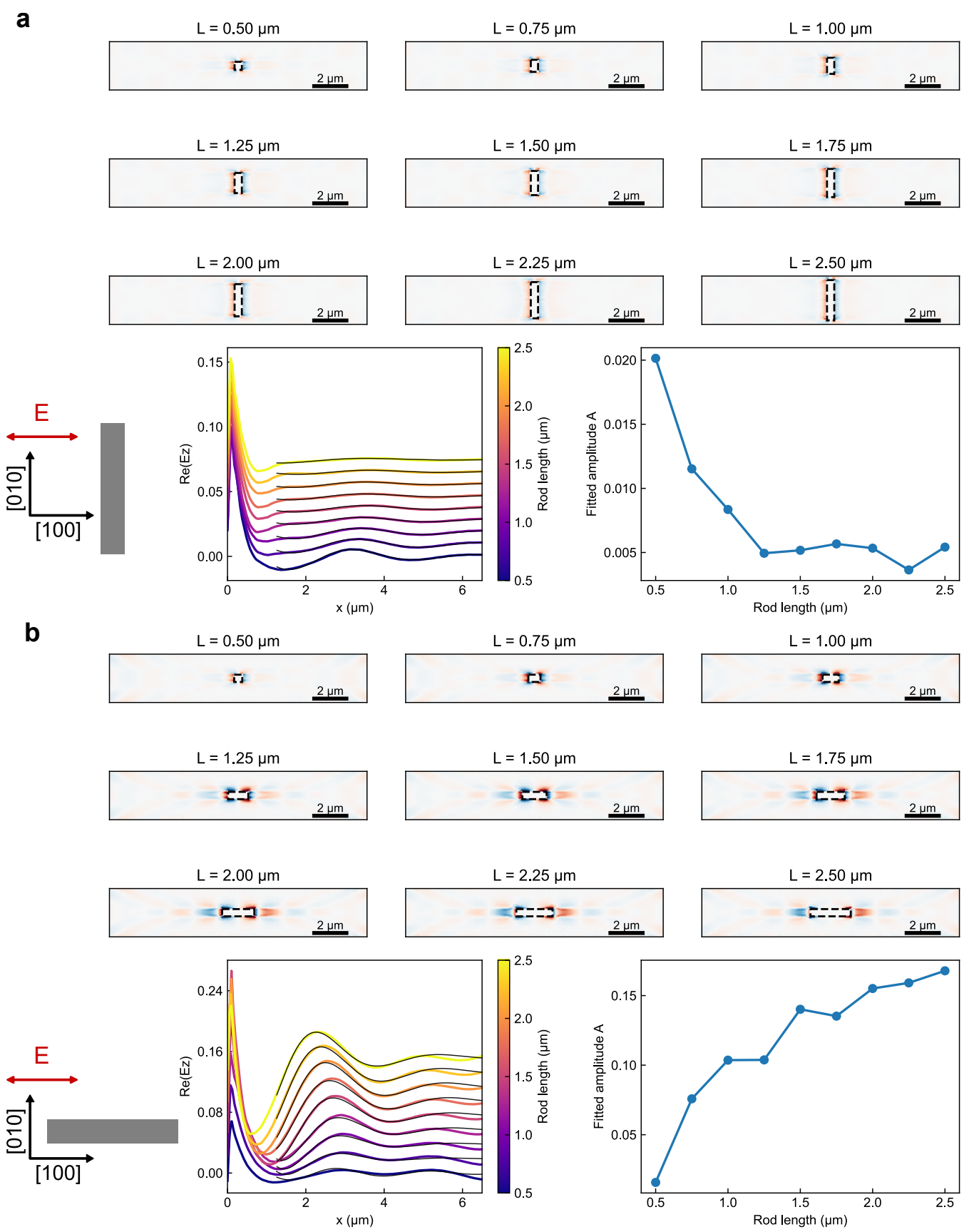

Fig. 7S: Simulated polariton launching in a 45 nm flake at the canalization frequency with polarization along the [100] direction. Simulated E_z maps launched by a rod of various lengths and fixed width. The rods are aligned with the [010] direction in **a** and with the [100] direction in **b**. Electric field profiles are fitted with an oscillating decay curve and the extracted amplitude is reported as a function of the rod length.

To clarify the role of the excitation geometry and the associated launching efficiency, we performed additional simulations at the canalization frequency for a 45 nm thick layer (Fig. 7S and Fig. 7S2). For simplicity, we considered

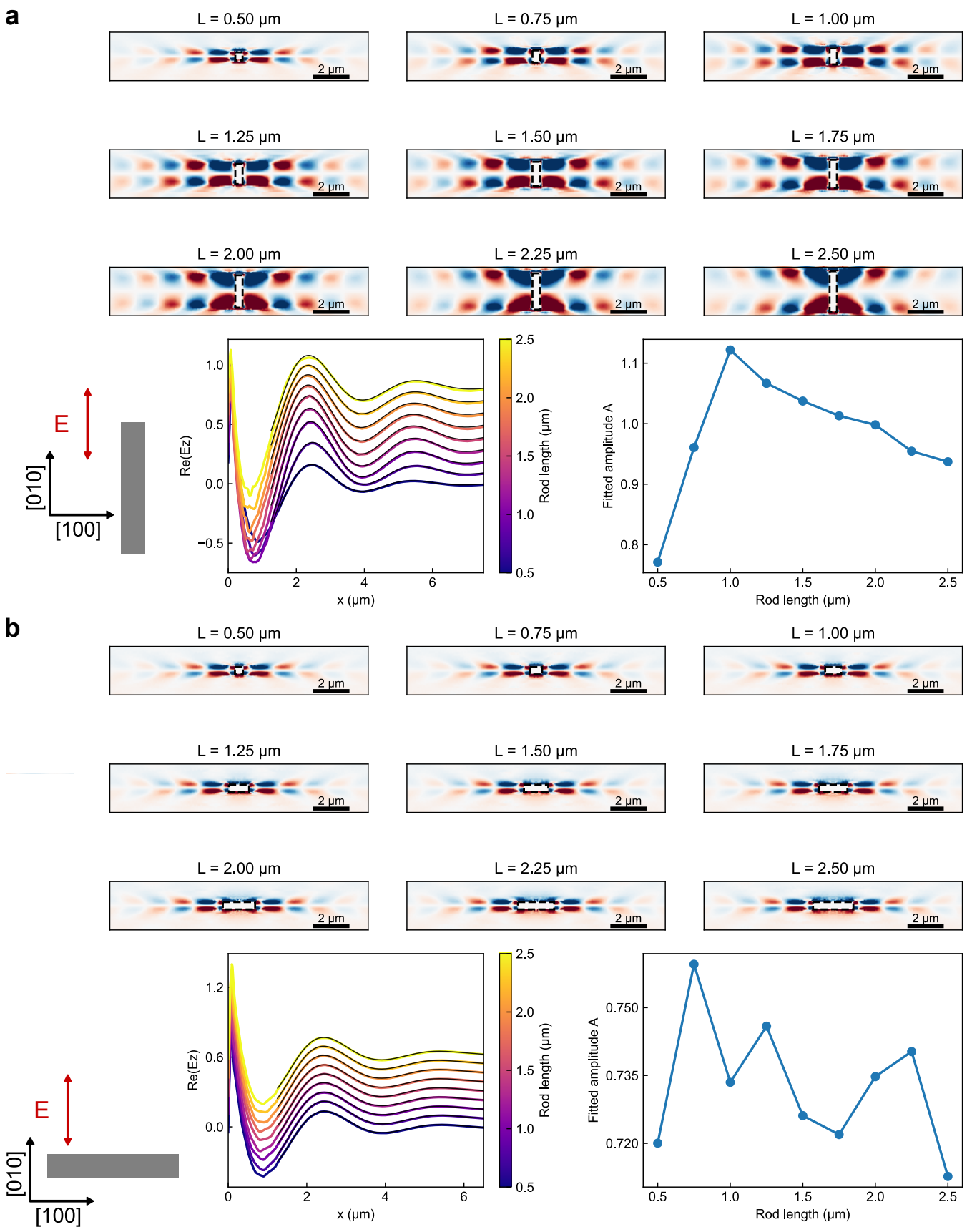

Fig. 8S: **Simulated polariton launching in a 45 nm flake at the canalization frequency with polarization along the [010] direction.** Simulated E_z maps launched by a rod of various lengths and fixed width. The rods are aligned with the [010] direction in **a** and with the [100] direction in **b**. Electric field profiles are fitted with an oscillating decay curve and the extracted amplitude is reported as a function of the rod length.

normal-incidence excitation with a linearly polarized plane wave and analyzed four configurations combining polarization along the [100] and [010] directions with rod antennas (modeled as perfect electric conductors with lengths between 450 nm and 2500 nm and fixed width of 450 nm) oriented along either [100] or [010]. For each configuration, we shows the simulated E_z field at the $MoOCl_2$ surface for each rod length and a sketch of the rod and polarization the exciting plane wave. For each rod length, the polariton profile was extracted from the simulated electric field (curves are vertically offset for clarity) and fitted with a decaying oscillatory exponential ($f(x) = Ae^{-\alpha x}\cos kx + \phi$) to quantify the launching efficiency via the extracted amplitude. We find that when the rod is aligned along [010] and the incident polarization is along

[100], the excitation remains weak for all rod lengths (Fig. 7Sa). When both rod and polarization are aligned along [100], the efficiency increases monotonically with rod length, indicating the onset of a resonance at larger sizes (Fig. 7Sb). The highest launching efficiency is obtained when both rod and polarization are along [010], where a clear maximum is observed around a rod length of ∼1.1 μm, consistent with a resonance-like behavior (Fig. 8Sa). Importantly, the propagation profile remains unchanged aside from intensity variations, indicating that antenna effects do not distort the canalized mode in the simulated conditions. In the experimental configuration (rod along [100], polarization along [010]), we observe robust and efficient excitation that is only slightly weaker than the resonant case and largely independent of rod length (Fig. 8Sb).

2.2 Understanding the advantage of using polarization aligned with the [010] direction

An intuitive understanding of the polarization dependence can be obtained from the overlap between the polariton isofrequency contours (IFCs) and the momentum distribution of an in-plane dipole representing the near-field excitation. The electric field of a dipole with moment $\mathbf{p}$ can be written as $\mathbf{E}(\mathbf{r}) \propto \mathbf{G}(\mathbf{r}) \cdot \mathbf{p}$, where $\mathbf{G}$ is the dyadic Green function $\mathbf{G} = (\mathbf{I} - \nabla\nabla/k_0^2)G_0$ with $G_0 = e^{ik_0 r}/(4\pi r)$ [5]. In momentum space, the dyadic Green function can be expressed using the Weyl identity (angular spectrumrepresentation) as:

$$\mathbf{G}(\mathbf{k}) \propto \left(\mathbf{I} - \frac{\mathbf{k}\mathbf{k}}{k_0^2}\right) G_0(\mathbf{k}), \tag{4}$$

where the scalar Green function in momentum space reads

$$G_0(\mathbf{k}) \propto \frac{e^{ik_z|z_0|}}{k_z}, \tag{5}$$

with z_0 the vertical position of the dipole and $k_z = \sqrt{k_0^2 - k_\parallel^2}$ the out-of-plane wavevector component. In the near-field regime relevant for polariton excitation, the dominant contributions arise from large in-plane momenta $k_\parallel \gg k_0$, for which $k_z \approx ik_\parallel$. In this limit, the second term of the Green tensor dominates, and the in-plane electric field becomes proportional to

$$\mathbf{E}(\mathbf{k}_\parallel) \propto \mathbf{G}(\mathbf{k}_\parallel) \cdot \mathbf{p} \quad \approx \quad \mathbf{k}_\parallel \cdot \mathbf{p}, \tag{6}$$

where $\mathbf{p}$ is the dipole moment. Therefore, the coupling strength at a given in-plane momentum is governed by the projection of the dipole onto the wavevector, and the corresponding emitted power scales as

$$|\mathbf{E}(\mathbf{k}_\parallel)|^2 \propto |\mathbf{k}_\parallel \cdot \mathbf{p}|^2 = k_\parallel^2 p^2 \cos^2\phi, \tag{7}$$

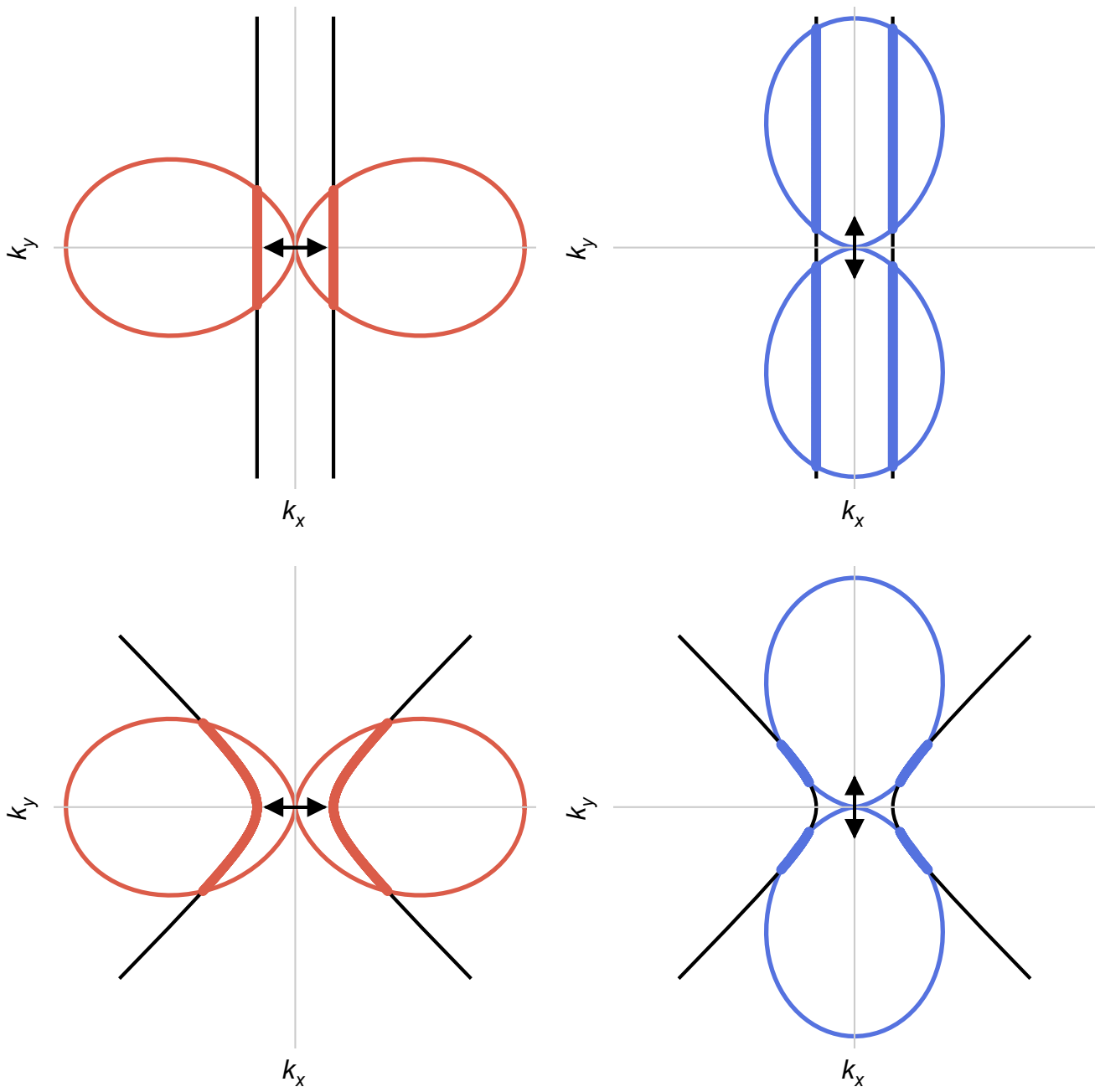

Fig. 9S: **Modelling the rod direction as near-field dipole orientation.** Momentum-space overlap between dipole emission and polariton IFCs. The angular distribution of an in-plane dipole is superimposed on representative IFCs for canalized (top) and hyperbolic (bottom) regimes. For canalization, where the IFCs are nearly vertical, a dipole oriented along [010] provides stronger overlap with the IFCs, leading to more efficient excitation of polaritons propagating along [100]. In contrast, for hyperbolic dispersion with IFCs opening along k_x, a dipole oriented along [100] exhibits improved overlap. Colored segments highlight the portions of the IFCs lying within the dipole emission distribution, indicating regions of enhanced coupling.

where ϕ is the angle between the dipole moment $\mathbf{p}$ and the in-plane wavevector $\mathbf{k}_{\parallel}$. By superimposing the in-plane dipole emission onto the polariton isofrequency contours, one finds that a dipole oriented along the [010] direction provides a stronger overlap with the IFCs at the canalization condition (Fig. 9S). In contrast, for hyperbolic dispersion where the IFCs open along the k_x direction, a dipole oriented along [100] exhibits improved overlap, consistent with previous observations in $MoOCl_2$ at visible and near-infrared frequencies [6, 7]. A more rigorous treatment of the angular emission would require inclusion of substrate-induced modifications of the dipole field and the reflected part of the Green function [8]. However, the simplified picture presented here captures the essential physics underlying the enhanced excitation efficiency observed for polarization along [010] at the canalization wavelength.

2.3 Experimental polariton launching under different polarization excitation

To experimentally verify the change in the polariton launching efficiency with different polarizations, near-field s-SNOM measurements were performed while rotating the in-plane projection of the incident polarization along the two principal crystal axes (Fig. 10S). As expected, when the polarization is aligned along the [010] direction, the resulting maps display more pronounced and extended interference fringes, indicating more efficient polariton launching compared to excitation along the metallic [100] axis.

This behavior is consistently observed for both disk and rod launchers. The rod antenna is elongated along the [100] direction. Additionally, this configuration offers a practical advantage: excitation with polarization along [010] suppresses the generation of edge-launched polaritons from the flake boundaries. This effect can be observed in the rod-based measurements, where residual edge-launched contributions are present for the other polarization configuration. As a result, using polarization along [010] relaxes constraints on device geometry, avoiding the need for large flakes or positioning the launcher far from the edges to minimize unwanted contributions.

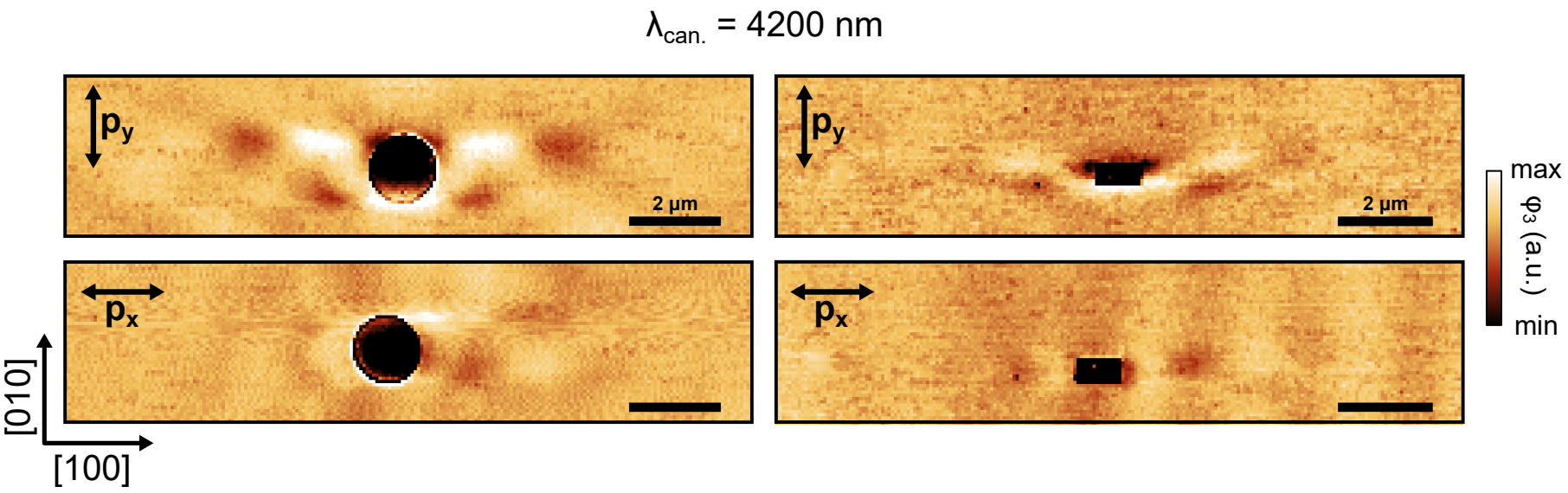

Fig. 10S: **Near-field phase maps for different incident polarization directions.** sSNOM measurement performed at 4200 nm on a 45 nm-thick flake. A gold disk and a rod oriented along the [100] direction were used as plasmonic launchers (scale bars: 2 µm). In both cases, when the polarization is along the [010], more efficient polariton launching is observed.

3 Additional sSNOM phase maps

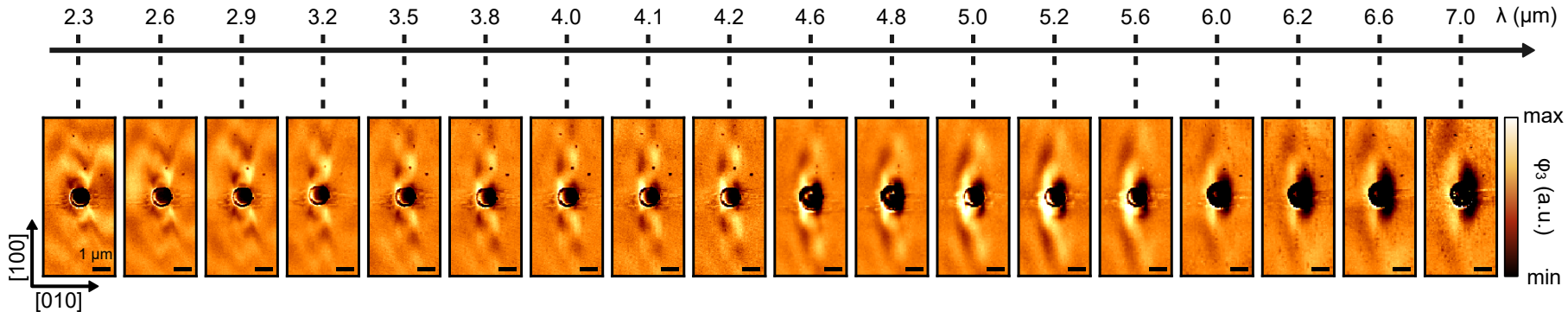

Fig. 11S: **Near-field imaging of the wavelength-driven transition with a disk launcher.** A gold disk is used as the plasmonic launcher while the excitation wavelength is swept across the hyperbolic, canalization, and elliptic regimes (scale bars: 1 µm, flake thickness 45 nm).

Figure 11S presents near-field phase maps of the third demodulation order obtained on a 45 nm-thick $MoOCl_2$ flake using a gold disk as the plasmonic launcher while sweeping the excitation wavelength. The polarization of the incident light is aligned along the dielectric [010] axis of the flake to maximize the excitation efficiency of the plasmon-polariton modes. At shorter wavelengths, the near-field phase maps display strongly diverging hyperbolic wavefronts, characteristic of the hyperbolic dispersion regime, where the in-plane permittivity components have opposite signs. As the wavelength increases, the wavefronts gradually straighten, evolving into a highly directional, non-diverging pattern that signifies the onset of the canalization regime, where optical energy propagates in a nearly diffraction-free manner. Upon further increasing the wavelength, the wavefronts bend and form closed, elliptical shapes, corresponding to the elliptic dispersion regime, where both in-plane permittivity components are of the same sign.

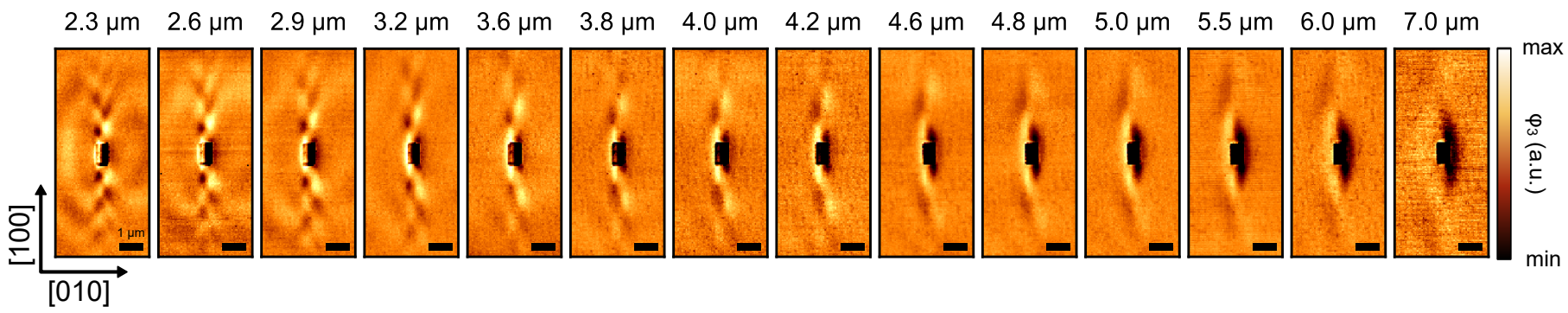

Fig. 12S: **Near-field imaging of the wavelength-driven transition with a rod launcher.** A gold nanorod is used as the plasmonic launcher while the excitation wavelength is swept across the hyperbolic, canalization, and elliptic regimes (scale bars: 1 µm, flake thickness 45 nm).

This continuous evolution of wavefronts provides a direct visualization of the topological transition from hyperbolic to elliptic propagation, with the canalization regime occurring at intermediate wavelengths. The observation highlights the intrinsic tunability of plasmon-polariton modes in $MoOCl_2$

through wavelength variation alone. Importantly, the same qualitative behavior is observed when using a gold nanorod launcher (Fig. 12S), demonstrating that the mode evolution and the emergence of canalization are robust features of the material and do not strongly depend on the specific geometry of the plasmonic launcher (at least in the subwavelength limit here investigates). These results collectively confirm that $MoOCl_2$ flakes support broadband, room-temperature plasmon-polariton canalization, with a mode transition that can be continuously tuned by the excitation wavelength.

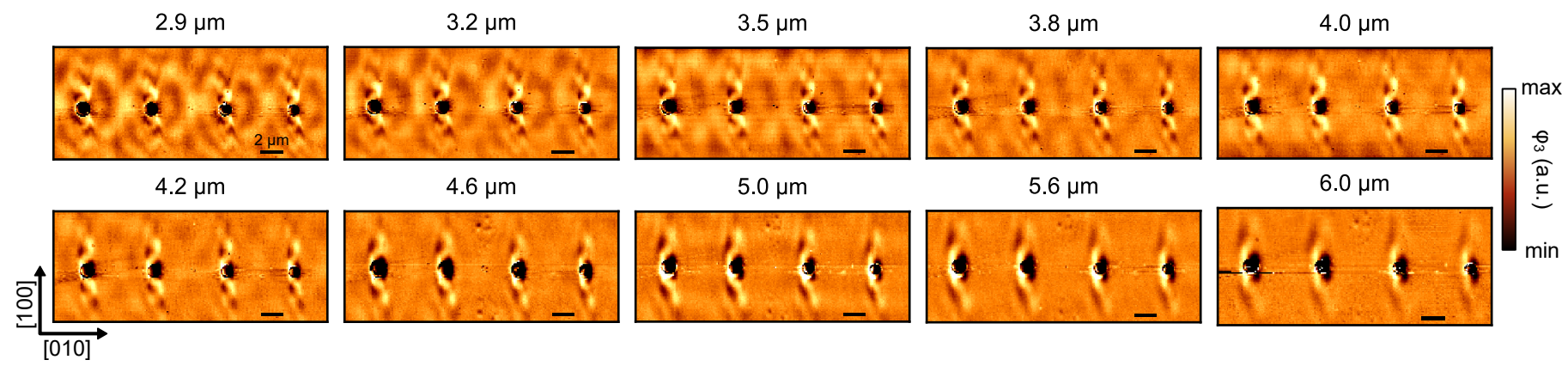

Fig. 13S: **Near-field imaging of the wavelength-driven transition with 4 disks launchers.** Gold disks of different sizes are used as the plasmonic launchers while the excitation wavelength is swept across the hyperbolic, canalization, and elliptic regimes (scale bars: 2 µm, flake thickness 45 nm).

The size of the gold launcher does not significantly affect the overall launching efficiency or the type of plasmonic modes excited (Fig. 13S). However, simulations (Fig. 14S) reveal subtle differences in the spatial distribution of the launched waves depending on the disk radius. For small, point-like disks (R = 100 nm), the launcher behaves effectively as a single point source, producing a concentrated hot spot from which polaritons propagate along the x-axis (metallic [100] crystal direction). As the disk radius increases, the induced oscillating charges driven by the incident polarization along the y-axis ([010] direction) are no longer uniform across the disk surface. This results in stronger near-field amplitudes at the top and bottom edges along the polarization axis, creating two effective excitation regions that act as separate sources for polaritons propagating along x. The appearance of these multiple hot spots can be understood as a combination of geometric extension of the launcher, anisotropic coupling efficiency of the disk edges to the polariton modes, and interference effects between the induced near-field regions. For even larger disks, additional portions of the disk contribute weaker fields, producing more complex near-field patterns and slight distortions of the propagating wavefronts, yet the fundamental mode type and propagation direction remain unchanged. These observations indicate that the multiple launching spots are not indicative of different mode types but rather arise from the spatial distribution of induced charges and the anisotropic coupling between the launcher and the plasmon-polariton mode, as confirmed experimentally (Fig. 13S). This demonstrates that the plasmonic mode structure is primarily governed by the

intrinsic anisotropic properties of $MoOCl_2$, while the launcher geometry mainly modulates the spatial profile of the excitation.

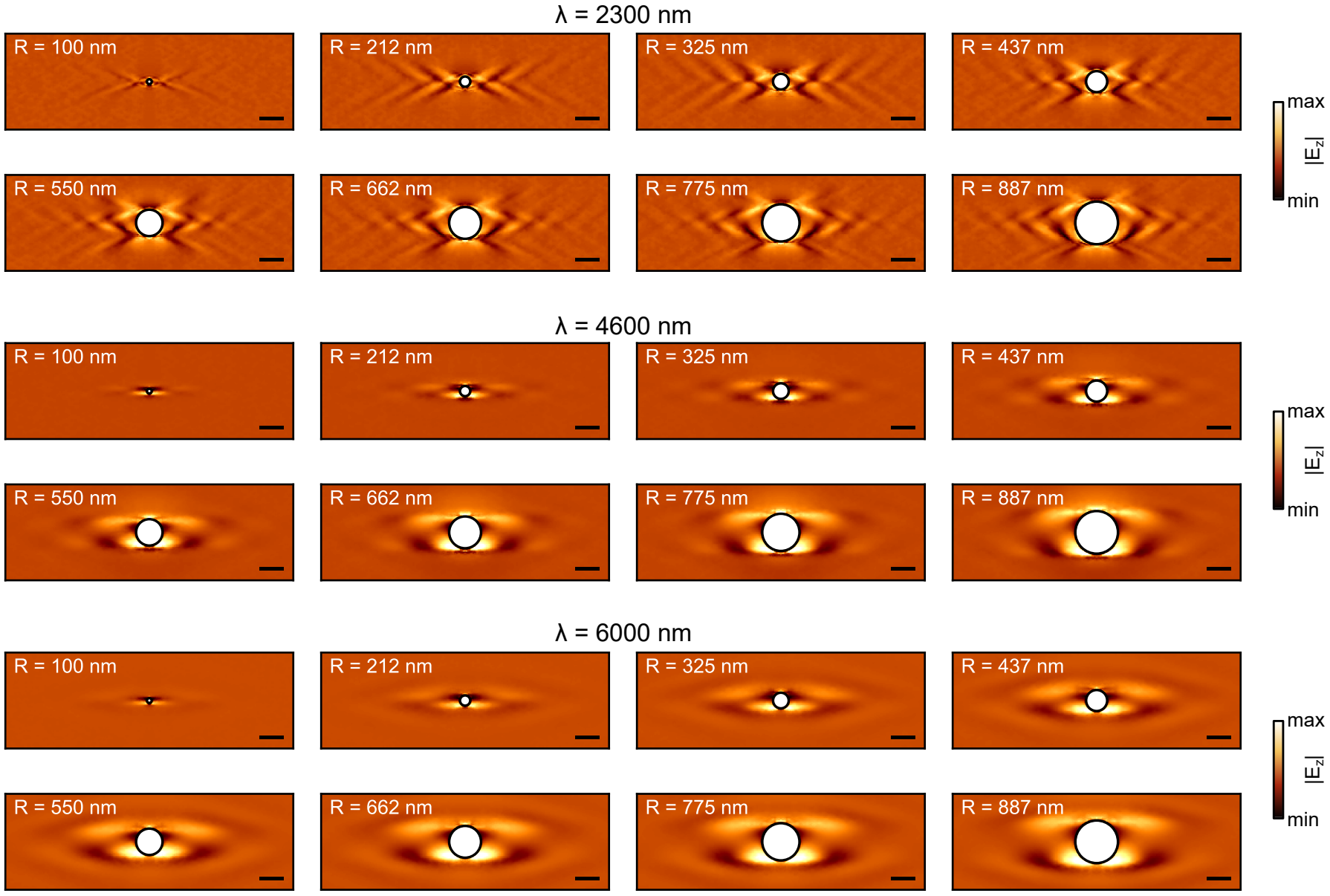

Fig. 14S: **Simulated out-of-plane electric field amplitude maps showing the wavelength-driven transition with a disk launcher.** A gold disk is used as the plasmonic launcher, with the disk radius varied from 100 nm to 887 nm. The flake thickness in the simulation is 45 nm. Simulations were performed for three representative wavelengths corresponding to the hyperbolic, canalization, and elliptical propagation regimes (scale bars: 1 µm).

To illustrate the transition of the plasmon-polariton modes for different flake thicknesses, we plotted a thickness–wavelength dependent matrix (Fig. 15S). This matrix provides a comprehensive visualization of how the wavefronts evolve across the hyperbolic, canalization, and elliptical regimes as a function of both excitation wavelength and flake thickness, clearly showing how the canalization wavelength shifts toward shorter values for increasing flake thickness. It highlights the tunability of the canalization effect through an intrinsic material parameter.

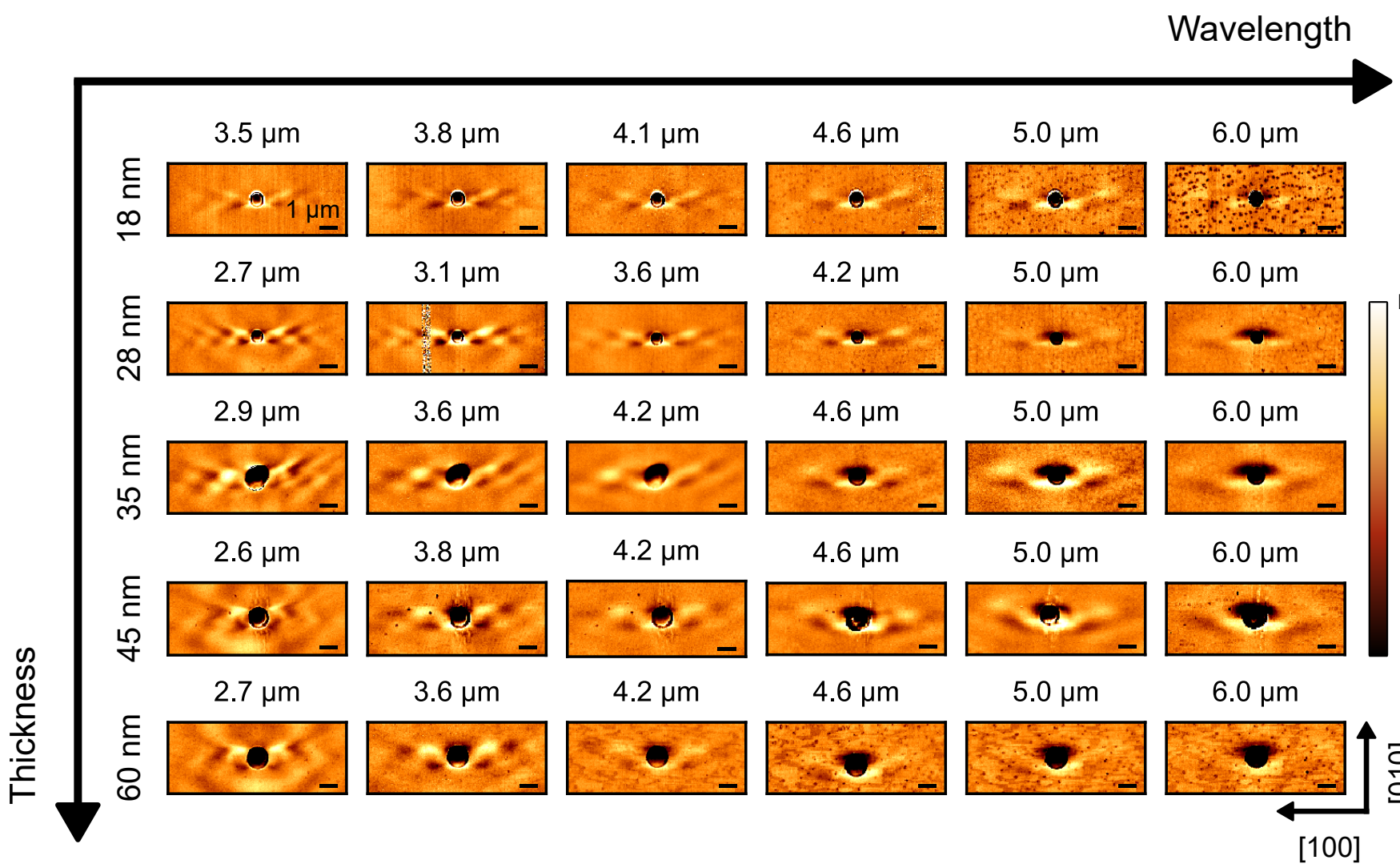

Fig. 15S: **Thickness–wavelength dependent matrix.** Near-field phase maps acquired by sSNOM with the excitation polarization aligned along [010], showing the dependence of plasmon-polariton canalization on incident wavelength and flake thickness.

4 Extraction of experimental IFC curvature

To quantitatively assess the topology of the experimental IFCs, we extract the vertex curvature (at $k_x = 0$) from the FFT maps. The full data-processing workflow is summarized in Fig. 16S. We begin with the raw sSNOM data—topography, amplitude, and phase maps. An example at $\lambda = 3500\,\mathrm{nm}$ for a 18 nm-thick flake is shown in Fig. 16Sa. The launcher edges are extracted as the boundaries of the adequately thresholded topography maps. Using these edges, we define rectangular regions on the left and right sides of each disk (Fig. 16Sb), and compute the FFT of each region separately. We use for the FFT computation only the phase data as they are generally less noisy than the amplitude ones and carry the same information for the purpose of the IFCs extraction. Prior to performing the FFT, we symmetrize the images about the axis where the scan intersects the disk. We additionally apply windowing on both the stitching boundary and the outer x and y edges, followed by zero-padding to increase the FFT resolution (Fig. 16Sc). To extract the IFCs from the FFT amplitude maps, we discretize the resulting images as follows. We first binarize the FFT maps using an intensity threshold and then identify connected components using the `bwconncomp` function in MATLAB. We exclude from this procedure the central region indicated by the dashed yellow line where a strong DC component is sometimes present. Exploiting the expected four-lobe symmetry, we process each connected region row by row. For every row, the center point of a given region is determined via an intensity-weighted

average of all pixels in that row belonging to the region. This yields a set of 2D points that trace the IFC (red points in Fig. 16Sd, the color follows the intensity of the FFT map at that point). These point sets are then fitted to the conic form $k_x^2/a^2 + \mu\, k_y^2/b^2 = 1$, where $\mu = 1$ corresponds to elliptical IFCs and $\mu = -1$ to hyperbolic IFCs. The fitted curves (green lines) accurately follow the theoretically calculated IFCs (blue lines). The vertex curvature is given by $\kappa = \mu\, a/b^2$. The final reported curvature is obtained by averaging the extracted values across all analyzed regions.

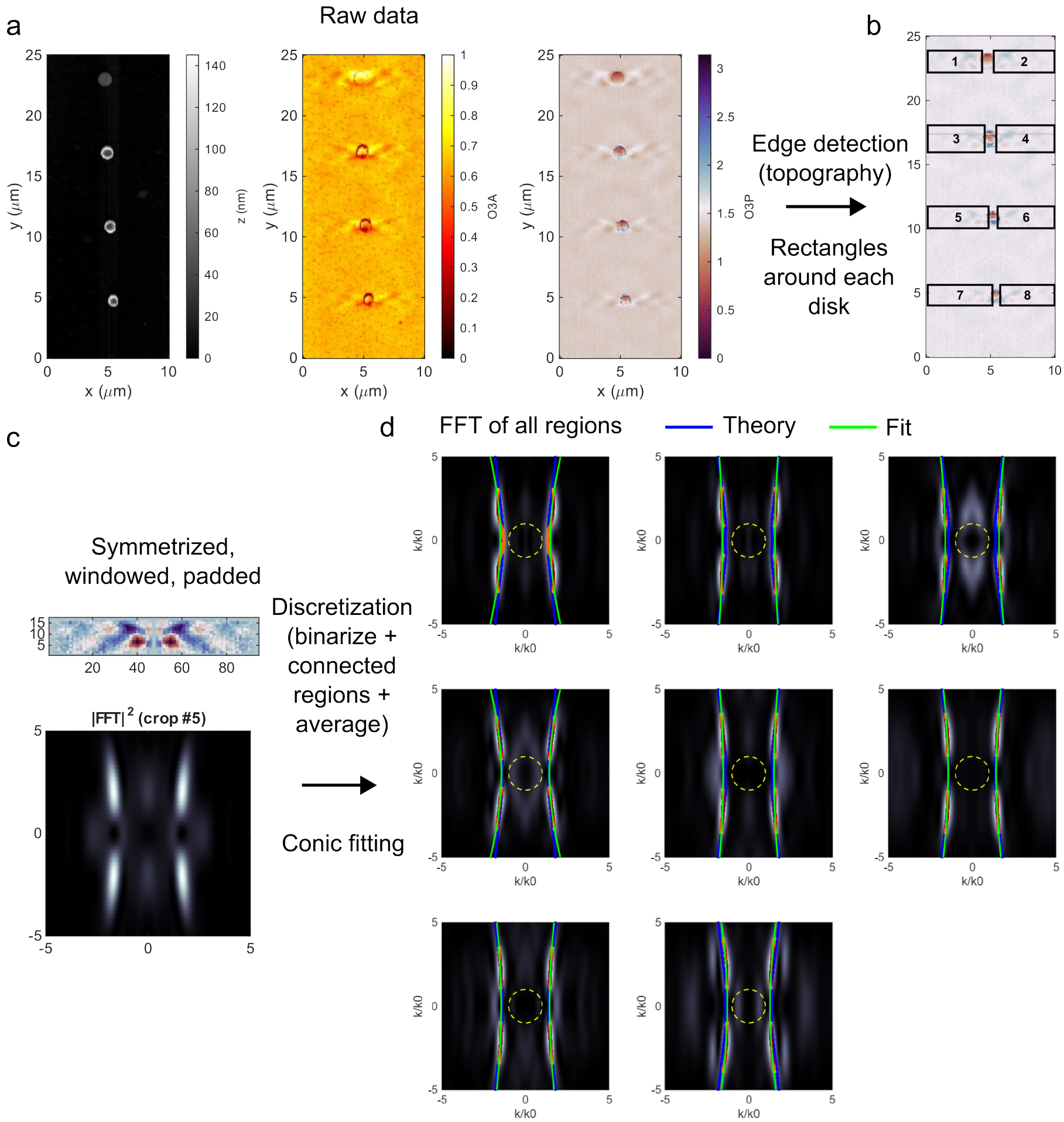

Fig. 16S: Experimental data treatment for the k-space images of polaritons IFCs. a Raw sSNOM data (topography, amplitude and phase) at $\lambda = 3500\,\mathrm{nm}$ for a 18 nm-thick flake. **b** Selection of separate regions to perform FFT obtained from edge detection of the disks from the topography image. **c** Before FFT we prepare the phase images by symmetrization and windowing in the center and at the edges in both directions. We then apply zero padding in order to increase the resolution of the FFT. **d** We discretize the FFT images by binarization and selecting the centerline of each connected region. A set of 2D points is extracted in this way, which we fit with a conic function, which allows the extraction of the IFCs curvature from the experiments.

5 Experimental thickness-dependent canalization frequency

Given the extraction of the experimental IFCs and the corresponding curvature, we can determine the canalization frequency by finding the frequency at which the curvature changes sign. To increase the reliability of the analysis, we use the average curvature extracted from all the different regions of the images (both disk sides and for different disk diameters) as detailed in the previous section. To fully exploit the extracted dataset, we determine the zero-crossing point by fitting the experimental curvature versus wavelength dependence with a third-order polynomial. This phenomenological approach is adopted because the curvature cannot be expressed in closed analytical form as a function of wavelength, given that the IFCs are governed by an implicit dispersion relation (see eq. (8) below). A third-order polynomial was found to be the lowest-order function that consistently provides an accurate fit across all thicknesses. The fitted results for all the thicknesses investigated in this work are reported in Fig. 17S. Gray dots are the single curvature values used to obtain the average (red dots), while black dashed curves are the polynomial fits. The spread of the individual data points mainly reflects variations associated with the excitation geometry and independent analysis of different launcher regions, although small local variations of the flake properties (e.g., residual strain, defects, or thickness inhomogeneities) may also contribute.

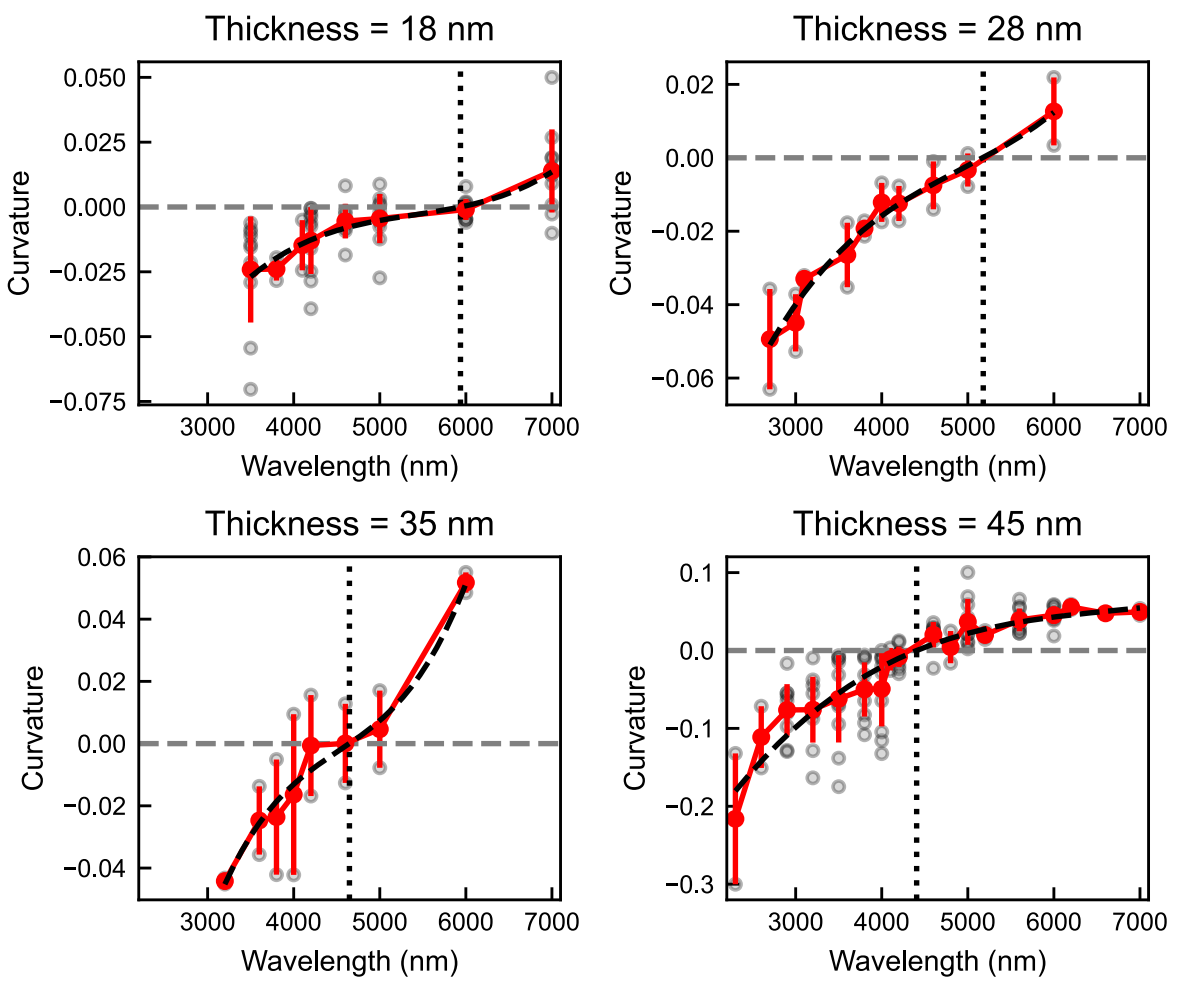

Fig. 17S: **Determination of the experimental thickness-dependent canalization frequency.** Fits of the experimental IFCs curvature against wavelength for all the $MoOCl_2$ flake thicknesses investigated in this work. Vertical dotted lines indicate the zero-crossing point of the IFCs corresponding to the canalization frequency. Individual data points are shown in gray, while averages are represented by red dots ± the standard deviation ($n = 2$ or $n = 8$ depending on the use of single disk or four-disk maps).

6 Theoretical thickness-dependent canalization frequency

In the limit of small thickness compared to the free-space wavelength $k_0 d \ll 1$, the in-plane wavevector of polaritons in a biaxial media can be obtained as the solution of the following equation [6, 9]:

$$\left[\frac{k_0 d\,\varepsilon_x}{2i}\,k_y^2 + \frac{k_0 d\,\varepsilon_y}{2i}\,k_x^2 + \frac{k_x^2+k_y^2}{2}\left(i\sqrt{k_x^2+k_y^2-\varepsilon_1} + i\sqrt{k_x^2+k_y^2-\varepsilon_3}\right)\right]$$
$$\times\left[\frac{k_0 d\,\varepsilon_x}{2i}\,k_x^2 + \frac{k_0 d\,\varepsilon_y}{2i}\,k_y^2 + \frac{k_x^2+k_y^2}{2}\left(\frac{\varepsilon_1}{i\sqrt{k_x^2+k_y^2-\varepsilon_1}} + \frac{\varepsilon_3}{i\sqrt{k_x^2+k_y^2-\varepsilon_3}}\right)\right]$$
$$= -\frac{k_0^2 d^2}{4}\,k_x^2 k_y^2\,(\varepsilon_x - \varepsilon_y)^2 \tag{8}$$

where ε_1 and ε_3 are the permittivities of the media above and below the biaxial film of thickness d. From the numerical solution of this equation, we obtain the theoretical IFCs shown in Fig. 1 and Fig. 2 of the main text. From the analytical IFCs we can also predict the change in canalization frequency

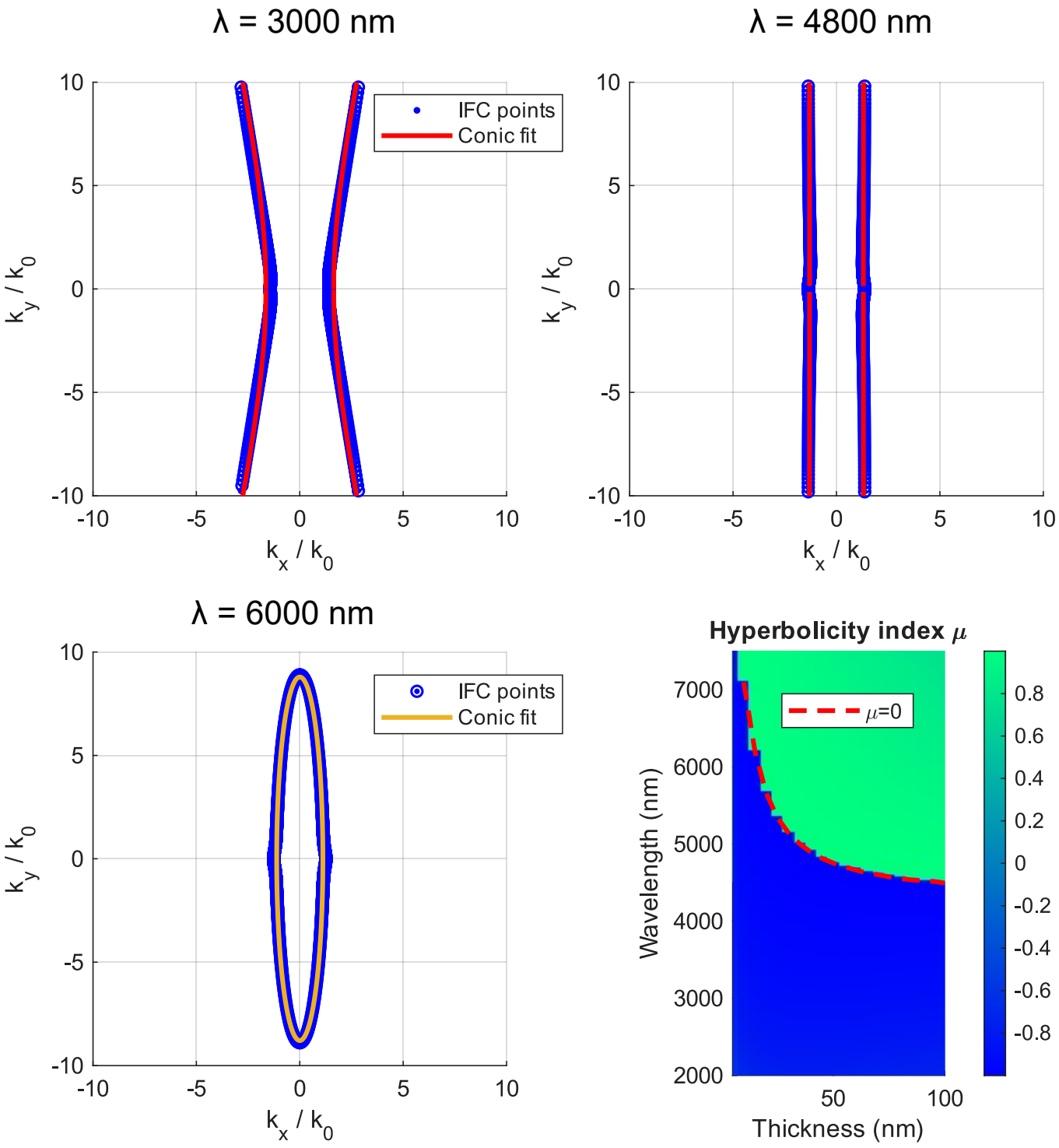

Fig. 18S: **Determination of the theoretical thickness-dependent canalization frequency.** Calculated IFCs for a 45 nm film thickness at various frequencies. The IFCs are fitted with the conic function. The color of the fit changes depending on the value of μ determining the IFCs topology. We also show the value of μ across the wavelength and thickness reported in Fig. 3 of the main text.

with film thickness. We perform a similar procedure to what we apply to the experimental data: we fit the analytical IFCs with the conic equation and extract the vertex curvature. In this way we construct the image shown in Fig. 3e of the main text. Examples of the analytical IFCs and corresponding conic fits with equation $k_x^2/a^2 + \mu\, k_y^2/b^2 = 1$ are displayed in Fig. 18S for a 45 nm thick film. We also report the extracted values of μ from the conic fit, which highlight the switch from the hyperbolic $\mu = -1$ to the elliptical regime $\mu = 1$.

The theoretical dispersion can also be used to gather an intuitive explanation of the redshift of the canalization frequency for decreasing flake thickness. First of all, we make sure that the presence of the substrate does not strongly affect the observed frequency shift (Fig. 19Sa). This is confirmed as we observe an almost identical thickness-evolution of the IFCs for a SiO_2 or vacuum substrates (at $\lambda = 5500$ nm). The only noticeable difference is the appearance of a circular component at the light line of the substrate in the SiO_2 case. Indeed, for substrates with a higher refractive index, the IFC shape can be strongly

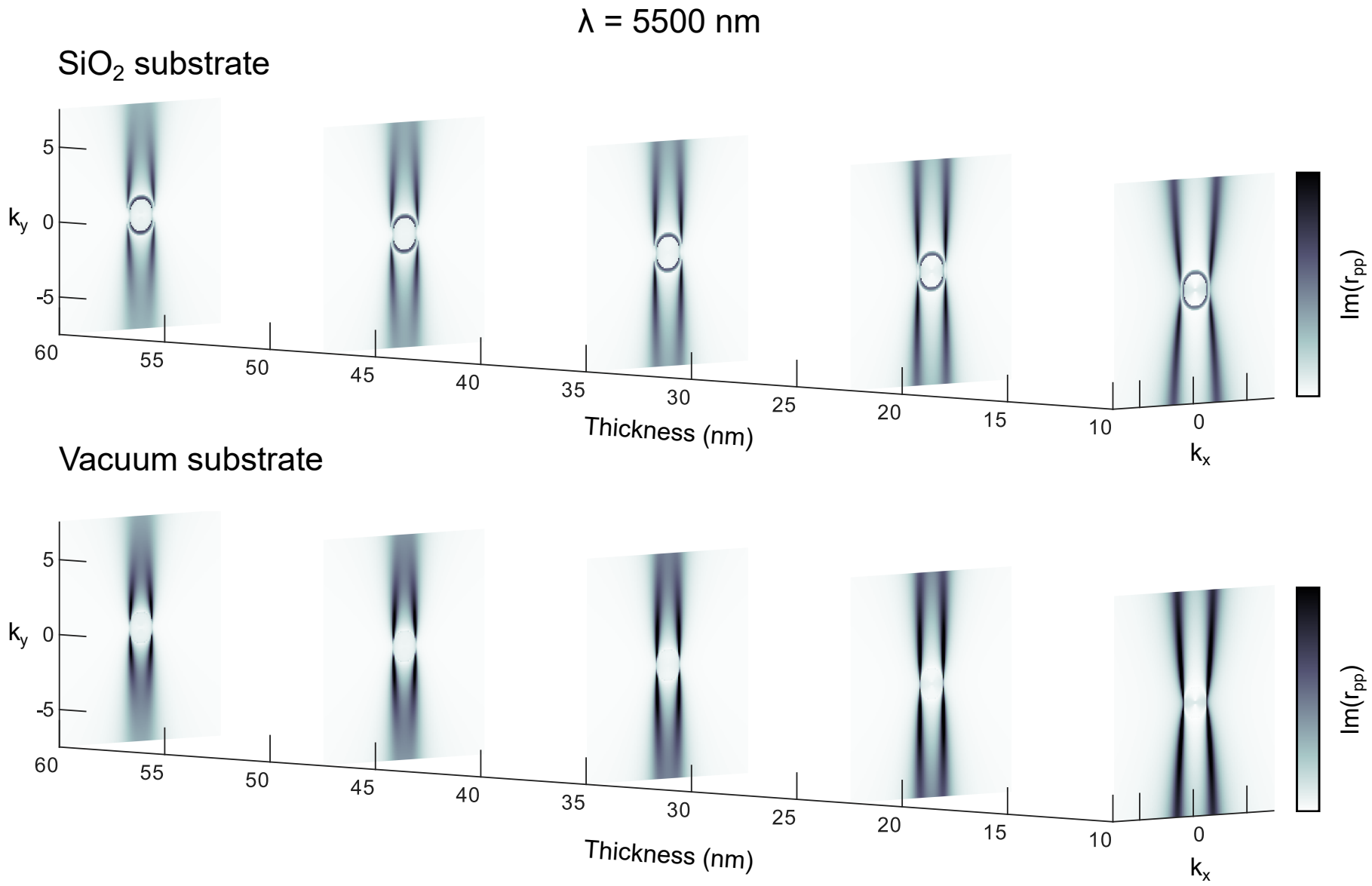

Fig. 19S: **Effect of substrate refractive index and dielectric function on canalization frequency shift.** Transfer matrix calculation of the evolution of the IFCs with film thickness for a SiO_2 substrate (top) or a vacuum substrate (bottom).

modified as they cannot enter the substrate light line and are thus pushed to higher wavevectors in a non-uniform way (they follow the circular shape of the light line).

To further clarify the physical origin of the canalization wavelength shift with thickness, we first consider the simplest possible case of a material with a non-dispersive dielectric response ($\varepsilon_x = -500 + 100i$ and $\varepsilon_y = -2 + 0.1i$, Fig. 21Sa, b), chosen such that canalization occurs at $\lambda = 5500$ nm for a thickness of 35 nm. In this analysis we restrict ourselves to a layer suspended in vacuum in order to exclude any contribution from the substrate refractive index. The corresponding IFC evolution, calculated via transfer-matrix methods, is shown in Fig. 21Sd. Interestingly, even for a completely frequency-independent dielectric function, a clear transition from hyperbolic to elliptical IFCs is observed as the thickness is varied. This demonstrates that the thickness-dependent shift of the topological transition is not solely driven by the dispersion of the dielectric function, but also by the intrinsic scaling between the free space momentum and the film thickness.

This behavior can be understood directly from eq. (8), where the thickness always appears multiplied by the free-space wavevector dk_0. As a consequence, increasing the thickness by a factor x can be compensated by decreasing the free-space wavevector by the same factor, corresponding to a linear increase of the canalization wavelength with thickness. Analytical calculations of the IFC

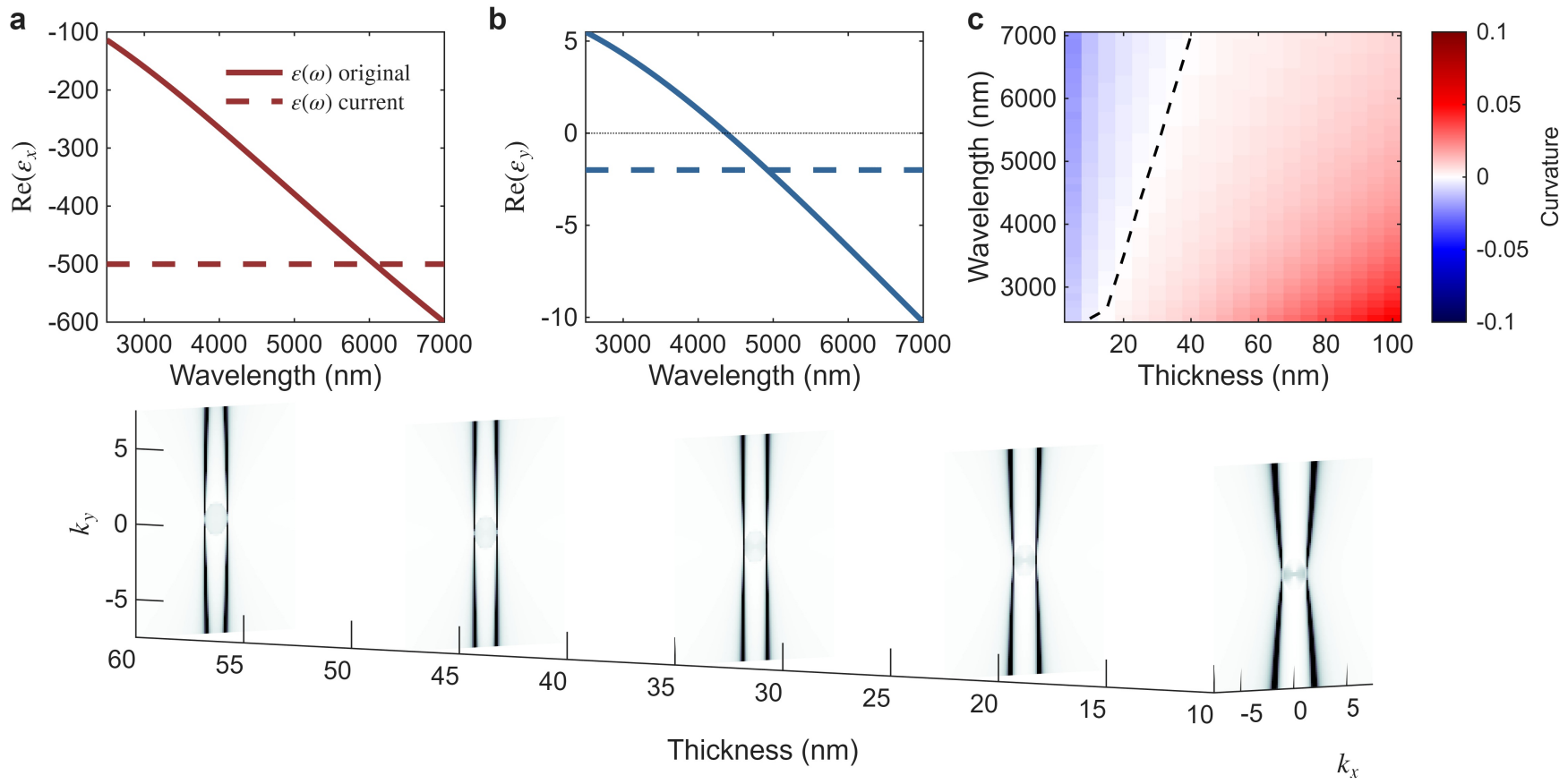

Fig. 20S: **Canalization shift for a material with constant dielectric function.** The used permittivity (dashed lines, $\varepsilon_x = -500 + 100i$ and $\varepsilon_y = -2 + 0.1i$) is shown along the $MoOCl_2$ mid IR dielectric function along the **a** x and **b** y directions. **c** Shift of the canalization frequency computed from the analytical dispersion as inferred from the zero of the IFC vertex curvature. **d** IFCs calculated from the transfer matrix at 5500 nm for flakes of different thicknesses.

evolution with tracking of the canalization wavelength shift with film thickness confirm this trend for the non-dispersive dielectric function (Fig. 21Sc).

This behavior is opposite to what is experimentally observed in $MoOCl_2$, where increasing the thickness produces a redshift of the canalization wavelength. Therefore, the dispersion of the Drude dielectric function must not only compensate but invert the intrinsic geometrical scaling described above. Since the dielectric-dependent terms enter the dispersion in the form $d, \varepsilon(\lambda_0)/\lambda_0$, a dielectric function whose magnitude decreases at longer wavelengths naturally reverses the trend imposed by the geometrical scaling, producing the experimentally observed evolution of the canalization condition.

To further isolate the role of the dielectric dispersion along the two principal axes, we separately analyze the effect of allowing only one component of the permittivity tensor to remain dispersive. We first consider the case where the y-direction is kept constant ($\varepsilon_y = -2 + 0.1i$), while the x-direction follows the dispersive dielectric function of $MoOCl_2$ (Fig. 21Sa, b). The resulting IFC evolution (Fig. 21Sd) is found to be very similar to the case of fully constant dielectric function, with the canalization wavelength still scaling approximately linearly with thickness (Fig. 21Sc). This indicates that the dispersion along the x direction has only a minor influence on the canalization frequency shift. Such behavior is expected, as ε_x remains strongly negative over the investigated spectral range, making the material response along this direction close to that of a perfect electric conductor.

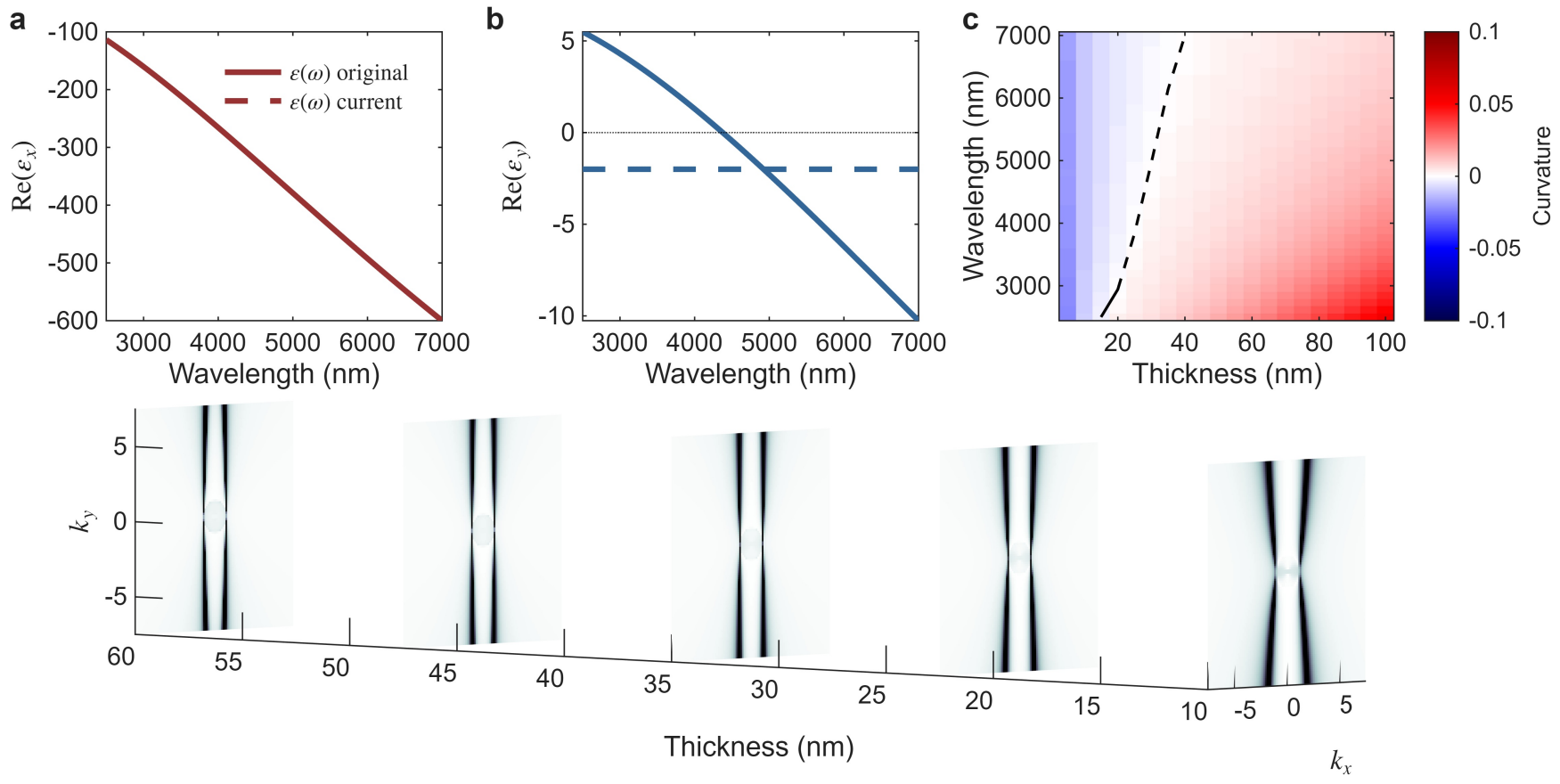

Fig. 21S: **Canalization shift for a material with the mid-IR dielectric function of MoOCl2 along x and a constant permittivity along y .** The used permittivity (dashed lines, $\varepsilon_y = -2 + 0.1i$) is shown along the $MoOCl_2$ mid IR dielectric function along the **a** x and **b** y directions. **c** Shift of the canalization frequency computed from the analytical dispersion as inferred from the zero of the IFC vertex curvature. **d** IFCs calculated from the transfer matrix at 5500 nm for flakes of different thicknesses.

We then consider the opposite limit, where $\varepsilon_x = -500 + 100i$ is kept constant while the dielectric response along the y direction follows the dispersive permittivity of $MoOCl_2$ in the mid-infrared (Fig. 22Sa, b). In this case, the IFCs evolve much more rapidly toward the elliptical regime as the thickness increases (Fig. 22Sd), leading to a qualitatively different behavior from the previous cases. Tracking the canalization wavelength through the analytical IFC calculations reveals a trend that resembles the experimentally observed evolution (Fig. 22Sc). These results demonstrate that the shift of the canalization wavelength is predominantly governed by the dispersion of the dielectric function along the y direction, while the dispersion along x only introduces minor quantitative corrections.

To assess the role of the [010] permittivity in setting the canalization condition, we calculated the IFC curvature evolution while keeping $\varepsilon_x(\omega)$ equal to the fitted $MoOCl_2$ response and fixing ε_y to selected non-dispersive values around zero (Fig. 23S). This allows us to isolate the effect of the [010] dielectric response independently of its dispersion.

For negative values of ε_y, the calculated zero-curvature line remains linear as a function of thickness, with values closer to zero shifting the canalization condition toward larger thicknesses and longer wavelengths. In contrast, when ε_y becomes positive (here we analyse the $Re(\varepsilon_y) = 0$, as the same happens for small positive values of $Re(\varepsilon_y)$), the IFCs remain hyperbolic within the thin-film approximation and the curvature approaches zero only asymptotically,

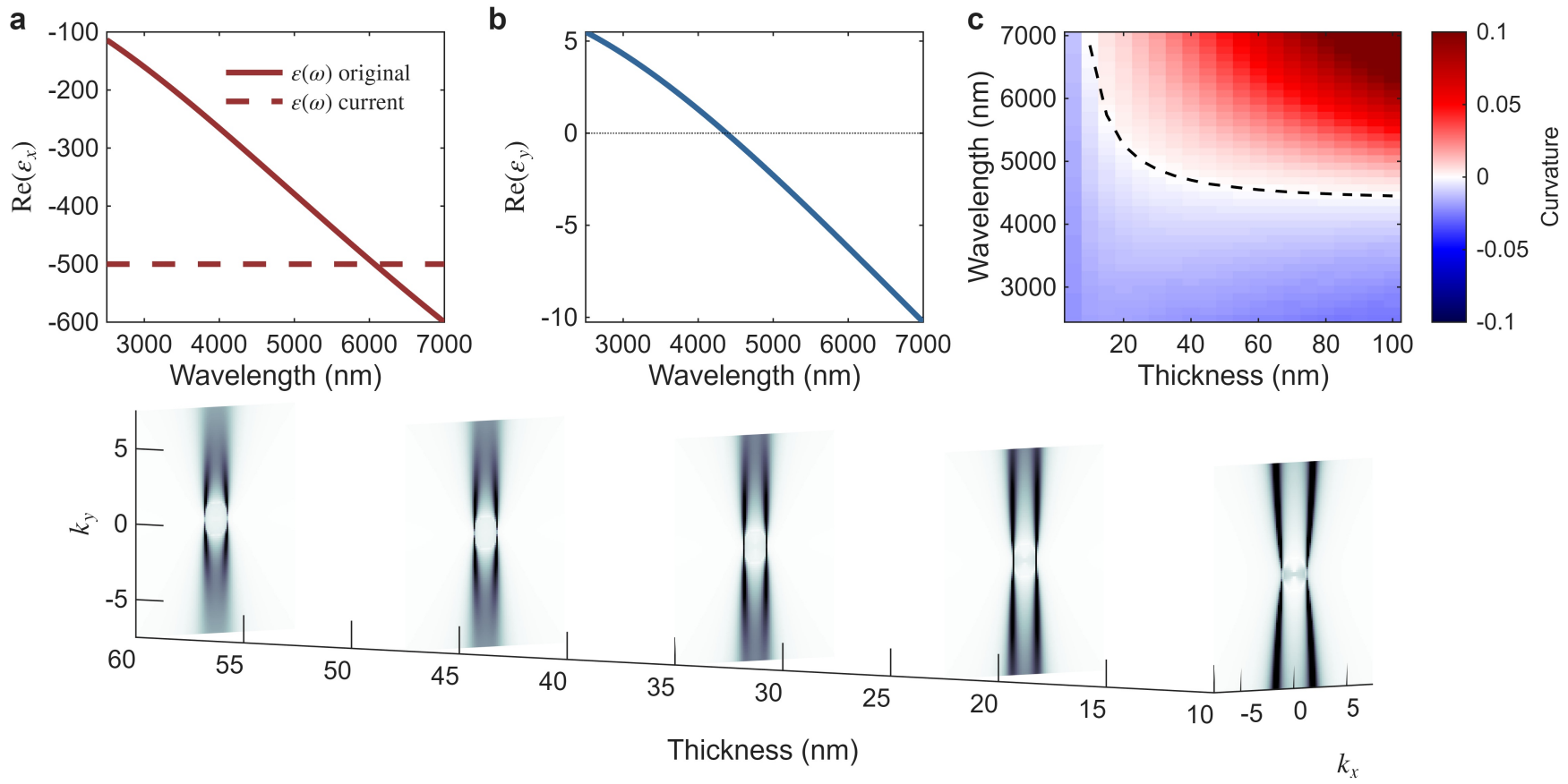

Fig. 22S: **Canalization shift for a material with the mid-IR dielectric function of MoOCl2 along y and a constant permittivity along x.** The used permittivity (dashed lines, $\varepsilon_x = -500 + 100i$) is shown along the $MoOCl_2$ mid IR dielectric function along the **a** x and **b** y directions. **c** Shift of the canalization frequency computed from the analytical dispersion as inferred from the zero of the IFC vertex curvature. **d** IFCs calculated from the transfer matrix at 5500 nm for flakes of different thicknesses.

without crossing into the elliptical regime. This behavior is also observed when extending the calculation to a broader thickness range, where the curvature remains small and negative over a wide spectral region (last panel of Fig. 23S).

These results show that a fixed small positive dielectric response along [010] is not sufficient to obtain a true hyperbolic-to-elliptical topological transition. We note that this analysis is performed within the thin-film approximation. At larger thicknesses, this approximation is expected to gradually break down, and the out-of-plane response ε_z also plays a role in determining the IFC shape. In this regime, the canalization condition is no longer governed solely by the in-plane components ε_x and ε_y, and the topological transition depends on the full biaxial dielectric tensor and its analysis more complicated.

6.1 Canalization wavelength shift in a Lorentz-Lorentz material

To assess whether thickness-dependent tuning of the canalization wavelength is expected in other van der Waals systems, we considered a representative Lorentz–Lorentz dielectric response corresponding to the typical case of phonon-polariton materials with Reststrahlen bands along both in-plane axes. As a model system, we started from the in-plane dielectric function of hBN [10] and rescaled the phonon frequencies such that the longitudinal optical phonon, and therefore the relevant sign-changing region of the permittivity, lies near 4.2 μm, comparable to the intrinsic canalization wavelength of

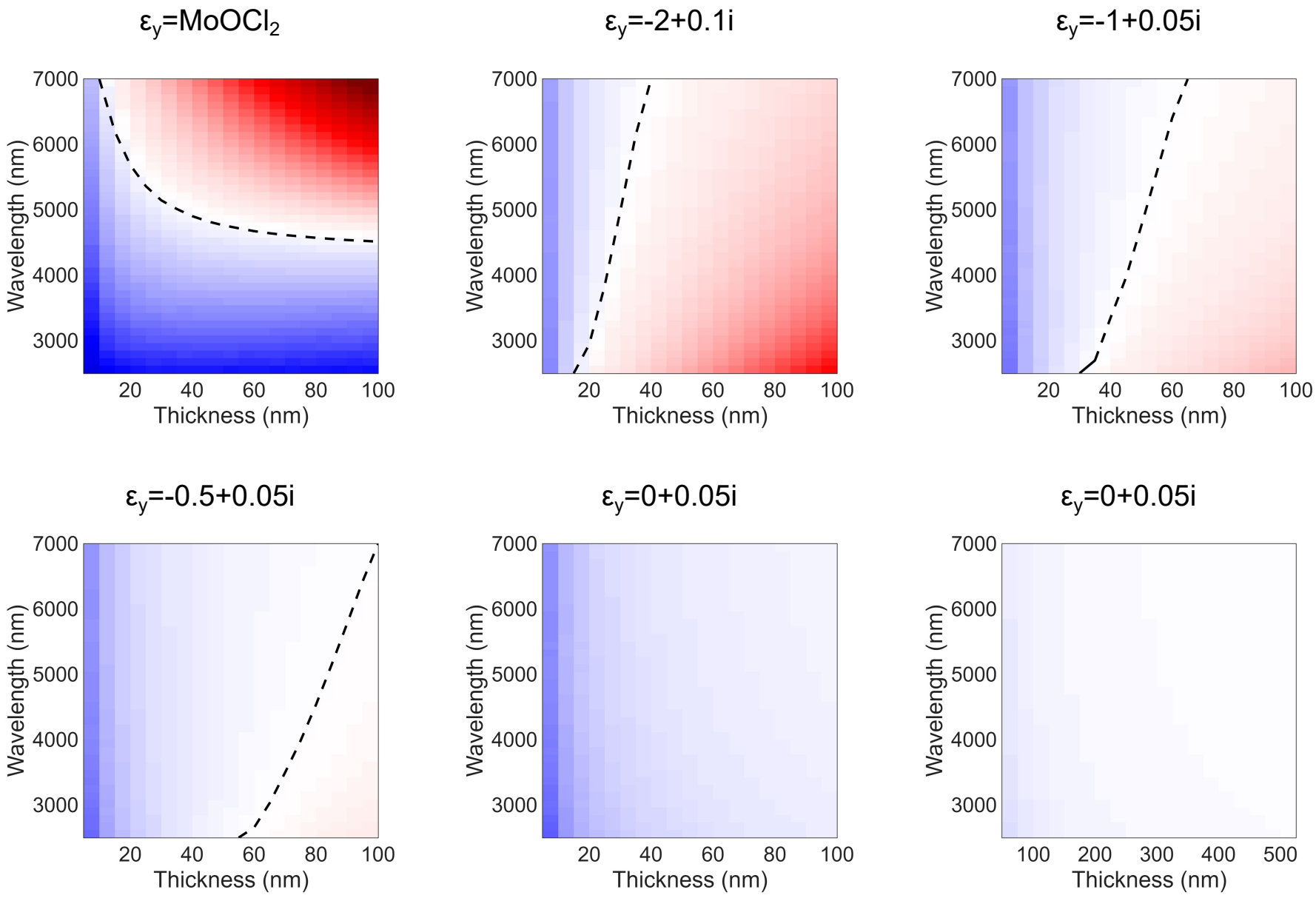

Fig. 23S: **Canalization shift for materials with the mid-IR dielectric function of MoOCl2 along x and a near-zero constant permittivity along y.** Calculated IFCs curvature in the small-thickness limit for different constant values of the [010] permittivity. Dashed black lines indicate the canalization condition.

$MoOCl_2$. The relative oscillator strength was preserved by keeping the quantity $(\omega_{\mathrm{LO}}^2 - \omega_{\mathrm{TO}}^2)/\omega_{\mathrm{TO}}^2$ constant, ensuring that the spectral rescaling does not artificially modify the dipole strength of the phonon resonance. An in-plane anisotropy was then introduced by shifting the second Reststrahlen band by a constant factor, applying the same scaling to ω_{TO}, ω_{LO}, and the damping rate. This generates adjacent hyperbolic and elliptical regions separated by a natural topological transition.

The resulting dielectric functions and corresponding IFCs evolution are shown in Fig. 24S for three anisotropy values (scaling = 1.05, 1.10, and 1.15 in panels a, b and c respectively). The upper panels report the real part of the dielectric function along the two in-plane axes, with the dashed gray line marking the wavelength of 4200 nm corresponding to the zero-crossing of the y-axis permittivity (around which the canalizaiton is expected to be). The permittivity sign change in the x direction is shifted toward shorter wavelengths, reproducing a situation analogous to $MoOCl_2$ where the two axes undergo the sign transition at different positions.

The middle panels show the corresponding isofrequency contours calculated via the transfer-matrix method in the 4400-4000 nm spectral range for a representative 50 nm film. As the separation between the two Reststrahlen bands

increases, the confinement of the modes decreases due to the increasingly negative value of ε_x, accompanied by reduced losses (sharper IFCs) resulting from weaker field penetration into the material. In all cases, the intrinsic topological transition between hyperbolic and elliptical propagation appears in the narrow 4300-4200 nm spectral range.

Fig. 24Sd tracks the evolution of the canalization wavelength with thickness for the three anisotropy values, obtained from analytical IFC calculations and extraction of the curvature through the same conic fitting procedure employed in the main text. In all cases, the canalization wavelength exhibits only a weak dependence on thickness and remains confined within the narrow 4300-4200 nm range. This behavior contrasts strongly with $MoOCl_2$, where over the same thickness range the canalization wavelength can be tuned by more than 1 μm. These results highlight how a Drude-Drude permittivity is substantially more favorable than a Lorentz-Lorentz one for achieving intrinsically broadband and thickness-tunable canalization.

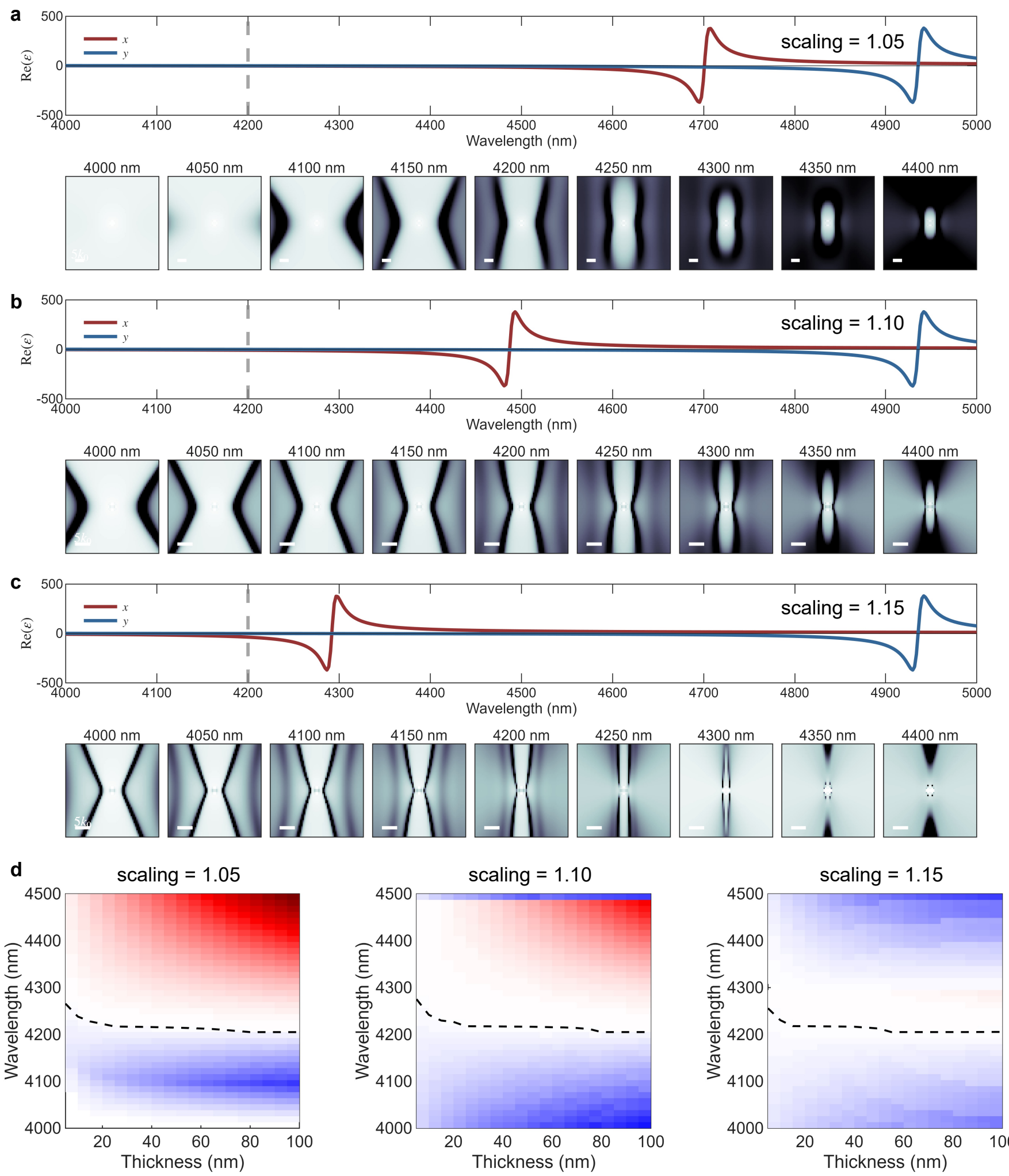

Fig. 24S: **Canalization wavelength shift in a model Lorentz-Lorentz material.** Real part of the in-plane dielectric function, with the dashed gray line marking the 4200 nm wavelength where the y-axis permittivity crosses zero. Each permittivity is characterized by the scaling between the two Lorentz oscillators with values of **a** 1.05, **b** 1.10 and **c** 1.15. For each permittivity, the corresponding isofrequency contours calculated via transfer-matrix methods in the 4000-4500 nm spectral range are shown. **d** Extracted canalization wavelength as a function of thickness, obtained from analytical IFC calculations and conic fitting of the IFC curvature for all three permittivities.

7 Propagation length of anisotropic plasmon polaritons

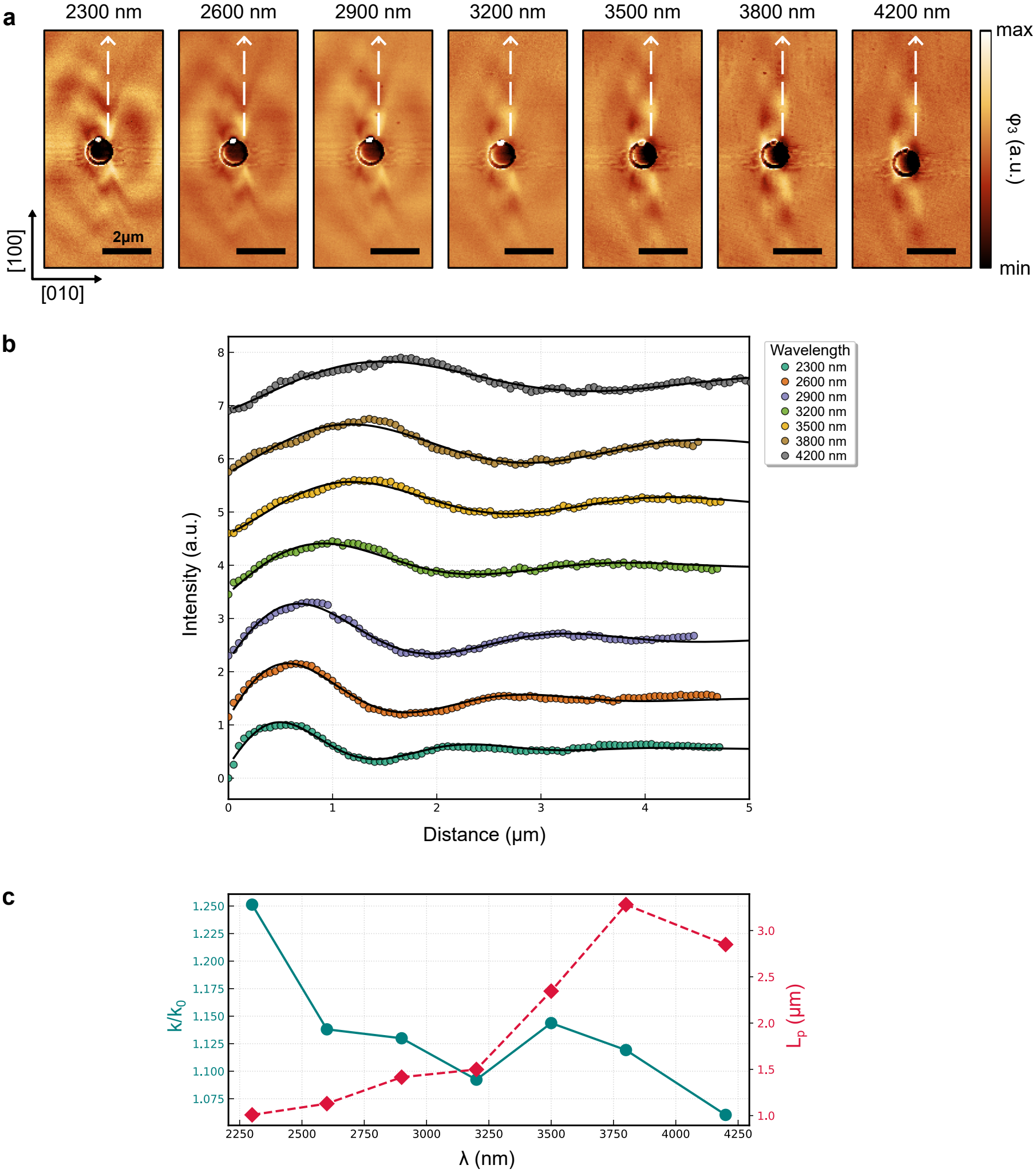

Fig. 25S: **Polariton propagation length on a** 45 nm **flake launched by a disk antenna. a** sSNOM phase maps from which decay profiles are extracted along the white dashed lines. **b** Extracted profiles fitted with an exponentially decaying function. Curves are offset for clarity. **c** Retrieved wavevector and propagation length as a function of wavelength.

To quantify the propagation length of mid-IR polaritons in $MoOCl_2$, we extract decay profiles along the propagation direction for the measurements

taken on the 45 nm flake at different wavelengths. We investigate launching from both a disk antenna (Fig. 25S) and a rod antenna (Fig. 26S).

In panel a of each figure, we report the near-field s-SNOM phase maps at different wavelengths. The white arrow indicates the direction along which the polariton profiles are extracted. Panel b shows the corresponding line profiles (offset for clarity), together with fits (black lines) using a decaying exponential function $f(x) = \exp(-\alpha x)\cos(kx + \phi) + C$. From these fits, we extract both the polariton wavevector k and the propagation length $L_p = 1/\alpha$.

The results are summarized in panel c, where we plot the extracted wavevector normalized to the free-space value, k/k_0, and the propagation length as a function of wavelength. We observe a decrease in the polariton wavevector accompanied by an increase in the propagation length as the wavelength increases. This trend is expected, as higher confinement (larger k/k_0) is generally associated with stronger losses and thus shorter propagation distances.

The analysis is performed up to wavelengths until the canalization condition, as beyond this wavelength polaritons become strongly damped, making it difficult to reliably extract a propagation length from the experimental data.

The increased damping observed near the canalization frequency can be attributed to the vanishing of the ε_y component, which leads to enhanced electric fields in the material. As a result, even moderate intrinsic losses are effectively amplified, yielding a reduction of the propagation length in the vicinity of the canalization condition.

To further investigate the evolution of the polariton propagation, we performed a thickness-dependent analysis of the polariton propagation length at wavelengths just below the canalization condition where it is easier to extract the propagation length (Fig. 27S). We observe that decreasing the flake thickness (thus redshifting the canalization frequency) is accompanied by an increase in the polariton momentum and a corresponding decrease in the propagation length. This behavior is consistent with the fact that achieving canalization at longer wavelengths requires thinner flakes, which results in stronger confinement of the polariton modes. As expected, increased confinement leads to enhanced losses and therefore shorter propagation distances.

8 Increasing canalization mode confinement

The moderate confinement of the canalized modes observed in the present geometry is mainly caused by the large negative permittivity along the [100] direction, which shifts the canalized IFCs toward relatively small in-plane momenta. To evaluate possible routes toward stronger compression, we analyze the confinement of the IFCs as a function of film thickness and wavelength. From the conic fit of the analytical IFCs, the value of the in-plane momentum at the $k_y = 0$ intercept can be extracted. Since the IFCs are nearly straight at the canalization wavelength, this value also represents the characteristic momentum of the whole canalized mode.

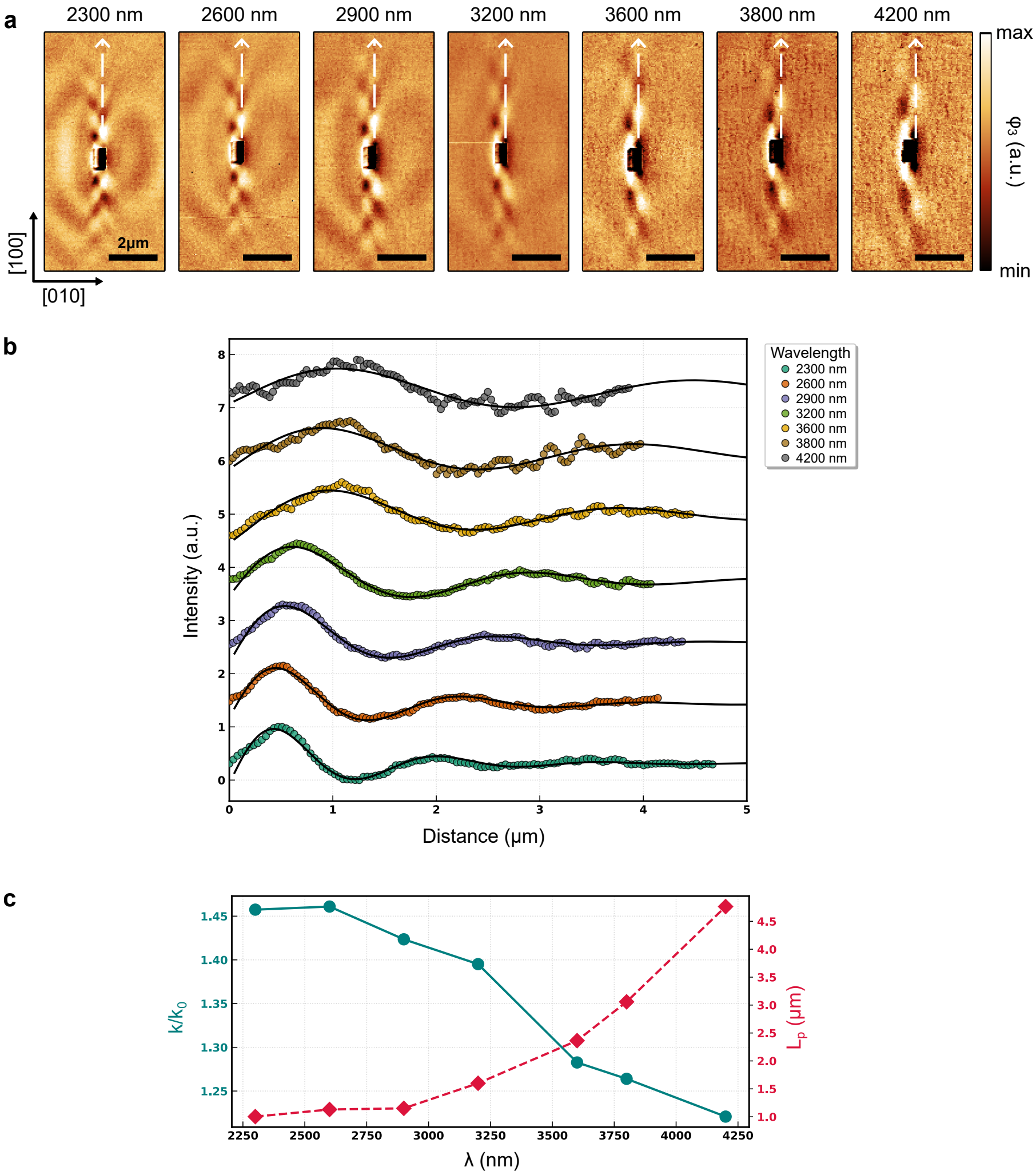

Fig. 26S: **Polariton propagation length on a** 45 nm **flake launched by a rod antenna. a** sSNOM phase maps from which decay profiles are extracted along the white dashed lines. **b** Extracted profiles fitted with an exponentially decaying function. Curves are offset for clarity. **c** Retrieved wavevector and propagation length as a function of wavelength.

Fig. 28S shows the extracted $k_x(k_y = 0)$ as a function of film thickness and wavelength. The white dashed line marks the canalization wavelength. The corresponding confinement factor extracted along this line is also shown. The result demonstrates that thinner flakes shift the canalized response toward larger in-plane momentum, and therefore provide a direct route to stronger modal compression.

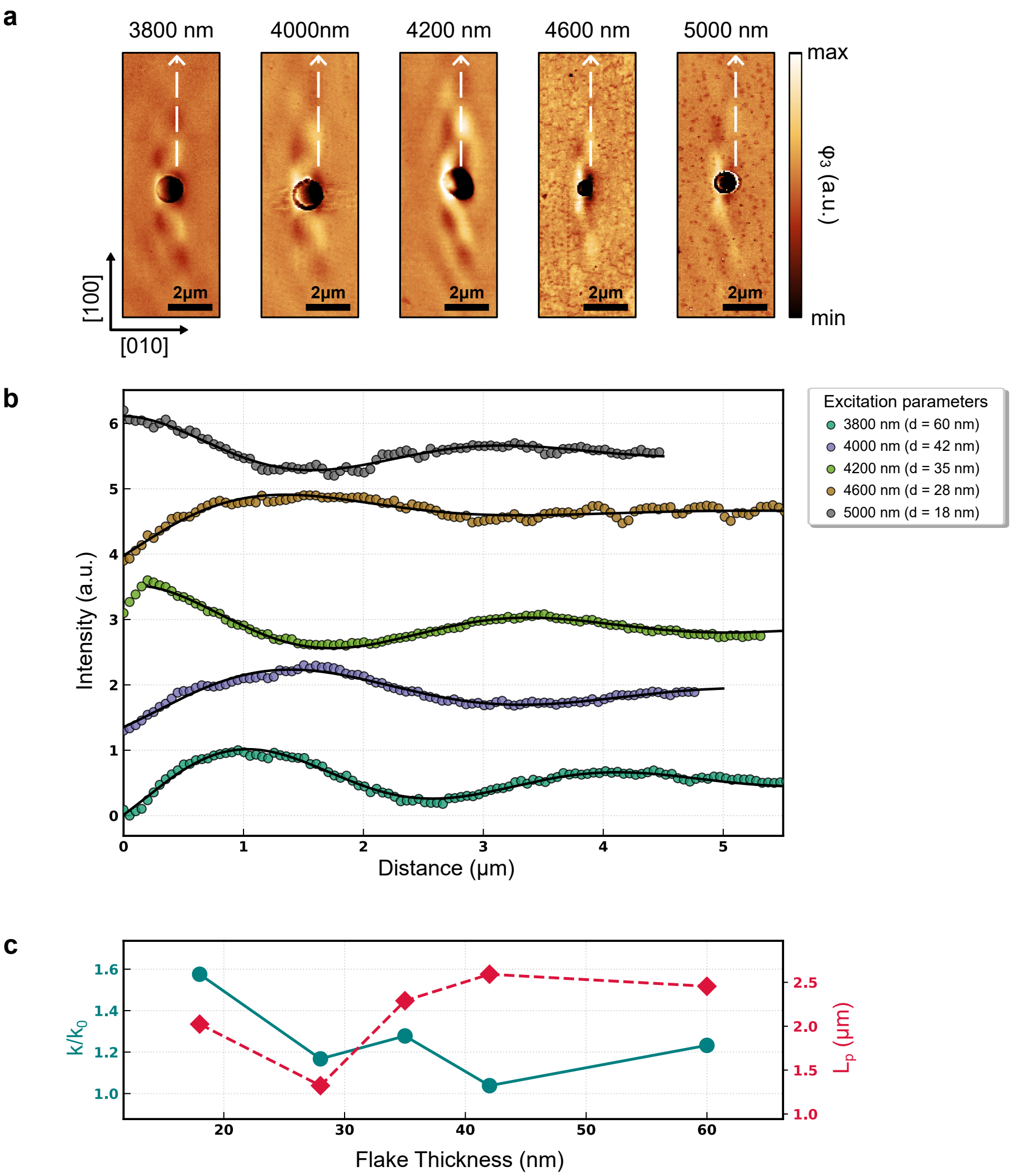

Fig. 27S: **Polariton propagation length at different film thicknesses.** **a** sSNOM phase maps from which decay profiles are extracted along the white dashed lines. Each map is taken at a different wavelength (just before the canalization condition) and corresponds to a different flake thickness (in order of decreasing thickness, with same values as in Fig. 17S.). **b** Extracted profiles fitted with an exponentially decaying function. Curves are offset for clarity. **c** Retrieved wavevector and propagation length for the different flake thicknesses (and thus different canalization frequencies).

A second route for enhanced confinemnt is substrate engineering. Fig. 29S compares two-dimensional transfer-matrix calculations for the same film placed

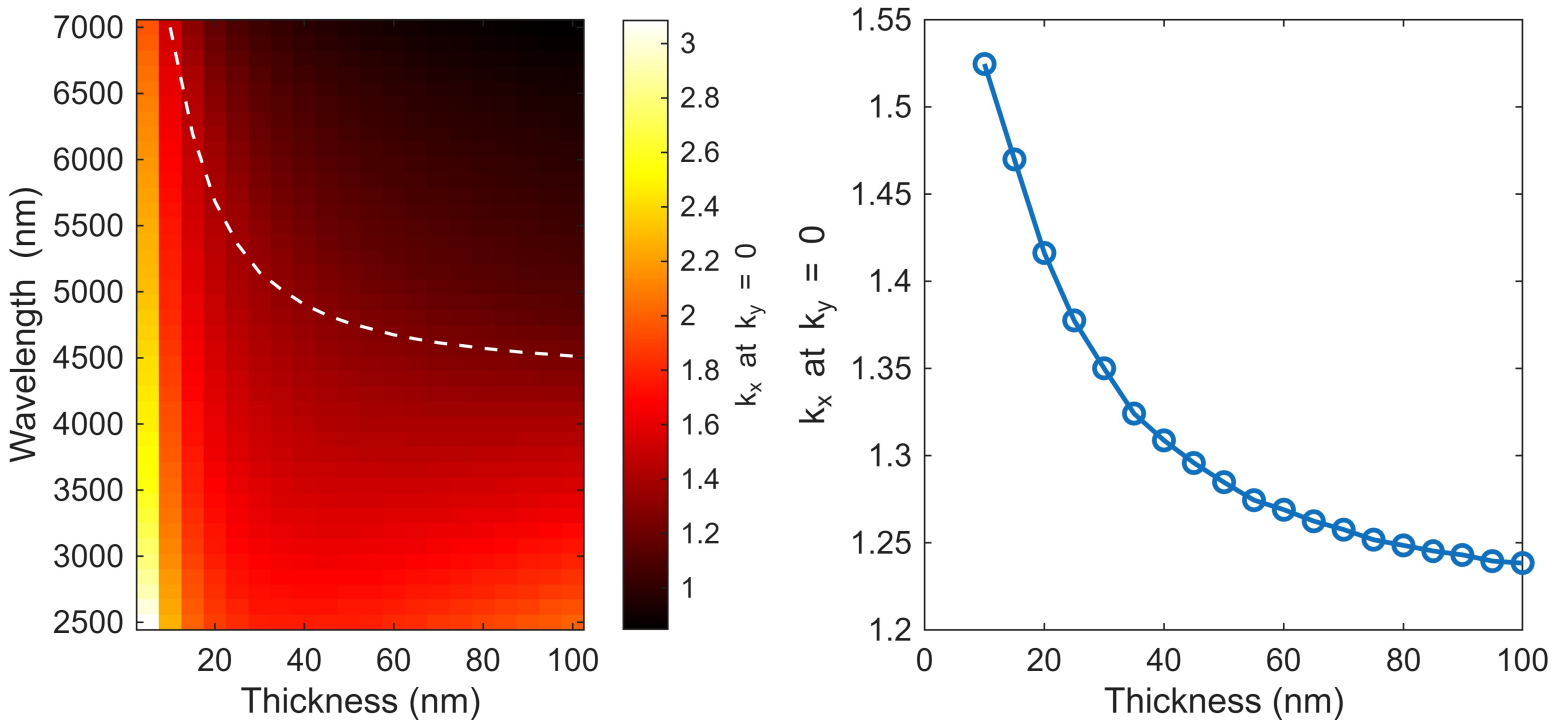

Fig. 28S: **Polariton confinement as a function of flake thickness.** Map of the extracted in-plane momentum $k_x(k_y = 0)$ as a function of film thickness and wavelength (left). The white dashed line indicates the canalization wavelength extracted for each thickness. Confinement factor extracted along the canalization line (right).

on a SiO_2 substrate and on an Au substrate. In the SiO_2-supported geometry, the lowest-order mode dominates the response and canalization occurs at relatively small momentum. In contrast, for the Au-supported geometry, the metallic boundary modifies the modal symmetry and suppresses the first mode through symmetry and screening at the metal interface [11]. Canalization can therefore occur on a higher order branch located at larger in-plane momenta, leading to stronger confinement.

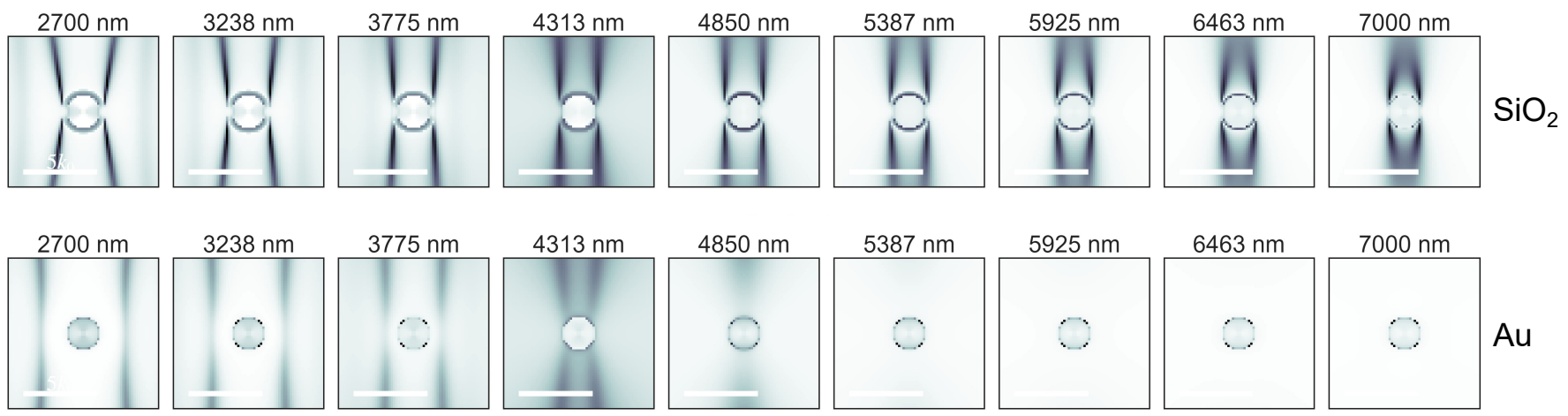

Fig. 29S: **Polariton dispersion on SiO_2 and Au substrates.** Two-dimensional transfer-matrix calculations of the polaritonic response for a $MoOCl_2$ film placed on a SiO_2 substrate (top) and an Au substrate (bot).

Furthermore, we performed measurements at a fixed wavelength of 2300 nm on ultrathin films with thicknesses of 3 nm and 6.6 nm to demonstrate that SPP modes can still be sustained in this extreme thickness regime. The results were compared with those obtained for a 42 nm-thick film, and the extracted polariton momentum and propagation length as functions of flake thickness

are summarized in Fig. 30S. As the film thickness decreases to 3 nm, the plasmon confinement increases dramatically, reaching values up to eight times larger than those observed in the thicker films, albeit at the expense of reduced propagation length. We also confirm (Fig. 31S) that the experimental momenta for the two thinner flakes are compatible with being tip-launched polaritons ($k_{exp} = 2k_{SPP}$ [12]) along the k_x direction reflected by the flake edge.

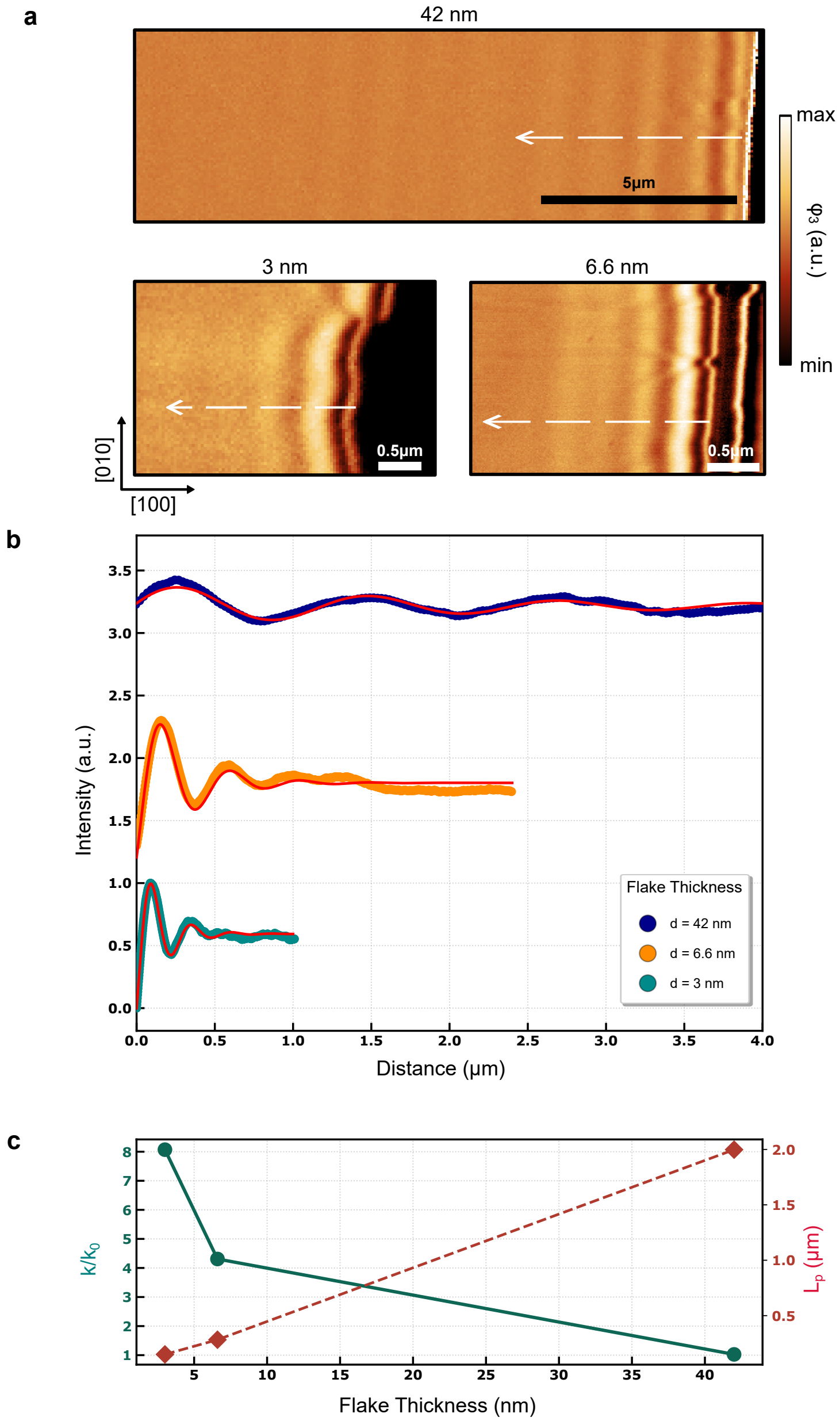

Fig. 30S: **Polariton propagation length at different film thicknesses.** **a** sSNOM phase maps from which decay profiles are extracted along the white dashed lines. Each map is taken at the same wavelength 2300 nm and corresponds to a different flake thickness. **b** Extracted profiles fitted with an exponentially decaying function. Curves are offset for clarity. **c** Retrieved wavevector and propagation length for the different flake thicknesses.

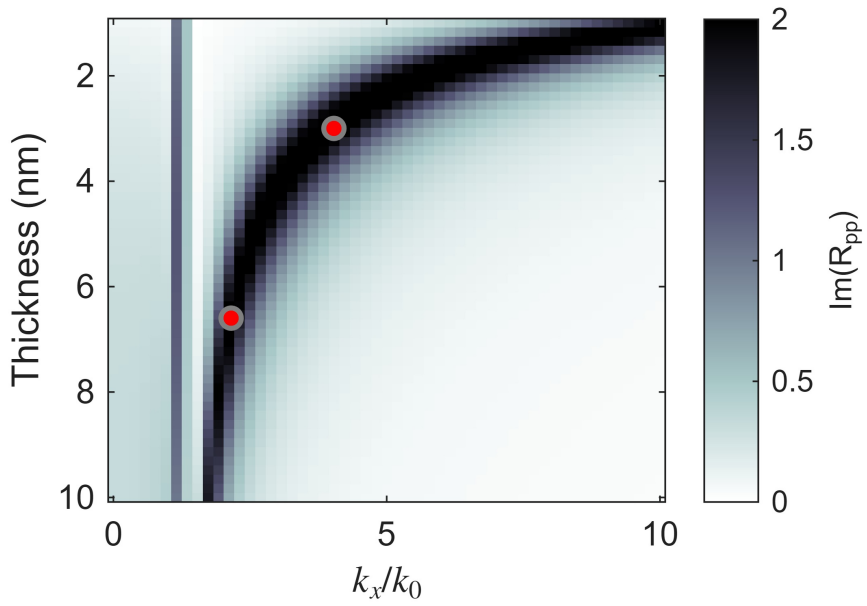

Fig. 31S: **Comparison of experimental momenta with transfer-matrix theory**. Experimental tip-launched momenta ($k_{exp} = 2k_{SPP}$) from the data in Fig. 30S overlayed on transfer matrix calculations for $MoOCl_2$ SPPs along the k_x direction at $\lambda = 2300\,\text{nm}$.

References

[1] Melchioni, N., Mancini, A., Nan, L., Efimova, A., Venturi, G., Ambrosio, A.: Giant optical anisotropy in a natural van der waals hyperbolic crystal for visible light low-loss polarization control. ACS Nano **19**(27), 25413–25421 (2025). https://doi.org/10.1021/acsnano.5c07323

[2] Dressel, M., Grüner, G.: Electrodynamics of Solids: Optical Properties of Electrons in Matter. Cambridge university press, Cambridge (2002)

[3] Zhao, J., Wu, W., Zhu, J., Lu, Y., Xiang, B., Yang, S.A.: Highly anisotropic two-dimensional metal in monolayer moocl 2. Physical Review B **102**(24), 245419 (2020)

[4] Ruta, F.L., Shao, Y., Acharya, S., Mu, A., Jo, N.H., Ryu, S.H., Balatsky, D., Su, Y., Pashov, D., Kim, B.S., *et al.*: Good plasmons in a bad metal. Science **387**(6735), 786–791 (2025)

[5] Novotny, L., Hecht, B.: Principles of Nano-optics. Cambridge university press, Cambridge (2012)

[6] Venturi, G., Mancini, A., Melchioni, N., Chiodini, S., Ambrosio, A.: Visible-frequency hyperbolic plasmon polaritons in a natural van der waals crystal. Nature Communications **15**(1), 9727 (2024). https://doi.org/10.1038/s41467-024-53988-7

[7] Li, Y., Zhang, Y., Zhang, W., Li, X., Tang, J., Xiao, J., Zhang, G., Liao, X., Jiang, P., Liu, Q., *et al.*: Broadband near-infrared hyperbolic polaritons in moocl2. Nature Communications **16**(1), 6172 (2025)

[8] Hu, C., Sun, T., Zeng, Y., Ma, W., Dai, Z., Yang, X., Zhang, X., Li, P.: Source-configured symmetry-broken hyperbolic polaritons. Elight **3**(1), 14 (2023)

[9] Álvarez-Pérez, G., Voronin, K.V., Volkov, V.S., Alonso-González, P., Nikitin, A.Y.: Analytical approximations for the dispersion of electromagnetic modes in slabs of biaxial crystals. Physical Review B **100**(23), 235408 (2019)

[10] Ambrosio, A., Tamagnone, M., Chaudhary, K., Jauregui, L.A., Kim, P., Wilson, W.L., Capasso, F.: Selective excitation and imaging of ultraslow phonon polaritons in thin hexagonal boron nitride crystals. Light: Science & Applications **7**(1), 27 (2018)

[11] Menabde, S.G., Boroviks, S., Ahn, J., Heiden, J.T., Watanabe, K., Taniguchi, T., Low, T., Hwang, D.K., Mortensen, N.A., Jang, M.S.: Near-field probing of image phonon-polaritons in hexagonal boron nitride on

gold crystals. Science advances **8**(28), 0627 (2022)

[12] Mancini, A., Nan, L., Wendisch, F.J., Berté, R., Ren, H., Cortés, E., Maier, S.A.: Near-field retrieval of the surface phonon polariton dispersion in free-standing silicon carbide thin films. ACS Photonics **9**(11), 3696–3704 (2022)
