# Supplementary material for: Intrinsic plasmon canalization in the biaxial van der Waals crystal MoOCl2
Source: Nat Nanotechnol. 2026 Aug 3;21(9):1238–44. doi: 10.1038/s41565-026-02243-9 (PMC13569449; doi:10.1038/s41565-026-02243-9)
Supplement: Supplementary file 2 [file 41565_2026_2243_MOESM2_ESM.pdf]

nature portfolio

Peer Review File

Intrinsic plasmon canalization in the biaxial van der Waals crystal $MoOCl_2$

Corresponding Author: Dr Andrea Mancini

This file contains all reviewer reports in order by version, followed by all author rebuttals in order by version.

Version 0:

Reviewer comments:

Reviewer #1

(Comments for the Author)
Intrinsic plasmon canalization in the biaxial van der Waals crystal MoOCl2
By Farid Aghashirinov and co-authors. This an interesting and timely work. Experiments are carefully performed. The manuscript is easy to read and easy to follow. Experimental results are well illustrated. In my opinion, this article will be of interest for a broader readership of Nature Nanotechnology.

Reviewer #2

(Comments for the Author)
The manuscript by Farid Aghashirinov et al. reports an intriguing observation of intrinsic plasmon-polariton canalization in $MoOCl_2$, supported by near-field imaging, numerical simulations, and analytical calculations. In particular, the authors demonstrate a thickness-dependent plasmon canalization frequency. The concept is of clear interest to the nanophotonics community. However, in its current form the manuscript lacks several further characterizations and clarifications that are necessary to justify publication in Nature Nanotechnology. A substantial revision addressing the points below is required.
1. The paper demonstrates directional confinement visually but does not report derived, quantitative metrics such as the propagation length as a function of wavelength within the canalization window.
2. The authors state that "we determine the zero crossing point by fitting the experimental curvature vs wavelength data with a third-order polynomial." The rationale for using a third-order polynomial should be explained.
3. I suggest the authors state, in the Abstract and Introduction, the physical reason why film thickness controls the canalization frequency. In the Conclusion, discuss whether this thickness dependence is expected to be universal for other biaxial or low-symmetry van der Waals crystals and under what conditions.
4. For figure 5S, please report reproducibility statistics: how reproducible is the canalization frequency (or curvature zero crossing) for flakes of nominally identical thickness? The current data show relatively large discreteness—explain the likely sources (thickness measurement uncertainty, flake-to-flake variability, local strain, orientation, defects, substrate effects, etc.).
5. For figure 1 S, the authors should show detailed fitting components for representative samples. Additionally, provide a sensitivity analysis: how do uncertainties in the fitted $\varepsilon(\omega)$ parameters affect the predicted IFC shapes and the canalization condition?

Reviewer #3

(Comments for the Author)
Aghashirinov et al. report on canalization at the topological transition between hyperbolic and elliptic polaritons in the van der Waals metal MoOCl2. They observe this transition in the mid-infrared around 4-5 microns, around the point of zero-crossing of the permittivity along the [010] direction. They show experimentally and in simulations that the canalization wavelength can be tuned by ~25% with the layer thickness of the MoOCl2 flake.

Overall, the work is very impressive, timely, and well executed, not only by demonstrating canalization in a single layer of a natural crystal, but also because by opening up a new but very interesting wavelength range in the mid-infrared which thus

far has been hardly accessible on van der Waals platforms. Thereby, the study certainly presents enough novelty to be published in Nature Nanotechnology.

I do have a few (not too major) concerns I would ask the authors to address before I can recommend the publication of their manuscript:

1) The arguments for choosing this specific geometry with a rod-like launcher illuminated perpendicular to the propagation direction are a bit unclear. In explaining Fig. 2a,b, the authors argue that the efficiency for polarization along the long antenna axis (Fig. 2a) is less efficient than for along the short axis (Fig. 2b). This suggests that there is some antenna resonance involved in the excitation which in fact one would typically try to avoid in order not to distort the near-field propagation with the spatial profile of the antenna resonance. It is strange that a geometry that specifically requires polarization transformation (Px -> Py in Fig. 2b), in particular where the authors argue earlier in the text on page 6 that "the in-plane px polarization direction play a crucial role in the excitation efficiency of the plasmon polaritons". The use of this experimental geometry seems to directly contradict this statement since here the excitation specifically does NOT contain this crucial polarization component, which is only provided indirectly through the antenna.

To be clear, I do not criticize this approach necessarily, but solely the argument why this is used. On the plus side, the vertical phase flip of the mode due to this geometry may in fact be helpful in the analysis (it's not a bug, it's a feature!) due to more distinct features in the propagation pattern, and potentially the authors could raise this point.

As for the geometry, I would have expected the authors to rotate the antenna such that the long axis is parallel to the propagation direction which would, following their arguments, allow to excite canalized polaritons with high efficiency and with an incident polarization matched to the propagation. I would argue that the modification of fringe spacing from collinear illumination (for small confinements), as argued by the authors being a problem, is not really a problem but rather an opportunity to have two complementary (forward and backward) representations of the canalized mode in one image.

I would kindly ask the authors to either do that experiment (rotated antenna, collinear p-pol excitation), or at least compare the respective simulations, i.e., same as Fig. 2a,b but with additionally rotating the rod antenna by 90°. I would also be useful to show the antenna resonance effect in more detail that is argued to be responsible for the different efficiencies in Fig. 2a,b.

2) The authors barely discuss propagation losses for these canalized modes. Overall, there is barely more than one fringe visible which would ask for – at least – a critical discussion of losses. Moreover, following the behavior of the permittivities, one would expect a trend for the propagation losses with wavelength. I would have guessed that with imaginary parts in both in-plane permittivity components going up with increasing wavelength, that also the propagation losses increase with wavelength. In fact, I would qualitatively even see this in Fig. 3b, where the quality of the 4.2 micron mode seems significantly better than the 6 micron measurement. I would kindly ask the authors to

a) elaborate on propagation losses in general for their work

b) discuss expected or even observed changes in the losses for canalization wavelength tuning

3) In Fig. 3e, the authors show numerical results for the thickness dependence of the topological transition. I was surprised to find in the SI, Fig. 7Sc, a very intuitive plot that would actually (potentially) explain the tuning mechanism, but which is not mentioned in the main text. The argument/observation that canalization emerges at a constant value of d*eps[100] is actually very interesting, and should be discussed in the main text.

In particular, it also means that the zero-crossing or "near-zero" behavior of the [010] permittivity is almost irrelevant in the given regime since the "other" direction seems to dominate the behavior. Is it solely a coincidence the [010] permittivity happens to also cross zero in this spectral region? Would the concept also work with a small-index dielectric (and non-dispersive) behavior along the [010] direction? I would love to see a more in-depth discussion of these questions in the revised manuscript, since this would allow to also understand the experiments in a more general way, beyond simply observing something "cool".

4) The canalized modes observed here overall have a rather limited level of confinement, likely related to the rather large negative permittivity along [100]. Could the authors sketch any credible scenario on how to further compress the canalized modes?

A few minor issues:

• Ref. 5 is not studying a vdW system as suggested in the place where it is cited. A more fitting reference specifically for that sentence could be: Kowalski et al., Nat. Mater. 24, 1735 (2025).

• Ref. 41. This is an interesting work, but I think the original study for substrate-induced topological transitions is more appropriate, or should at least be also cited: Duan et al., Sci. Adv. 7, eabf2690(2021).

• Considering that $MoOCl_2$ is a monoclinic crystal, it would be appropriate to also cite the first work (before ref. 48) on highly directional shear polaritons in a monoclinic system in the introduction, amongst what is now refs. 10-18: Passler et al., Nature 602, 595 (2022).

• The statement "[…], losses due [to] overlapping phonon resonances, […]" on page 3 is slightly misleading in my view. For one, the losses in most plasmonic systems are much greater than in most phononic systems. But more importantly, the authors use a Drude-Lorentz oscillator model to describe the material, so also here there is a phenomenological Drude oscillator involved. I believe, in particular considering the actual losses involved in the current work, this statement should be reframed/changed. It is really not a good one.

• Conclusion, page 12, "simple yet powerful", I would ask the authors to refrain from bolstering statements like this. How "powerful" or not the current work and concept will be remains to be seen. I am not questioning the work, only that specific piece of language.

• Ref. 21 appears to be incorrect. It should read: Nature Nanotechnology 21, 23 (2026).

Version 1:

Reviewer comments:

Reviewer #2

(Comments for the Author)
The paper is fine now and it can be accepted.

Reviewer #3

(Comments for the Author)
I am happy with the response of the authors and their revisions, and recommend publication of their manuscript in Nature Nanotechnology.

A. Paarmann

Open Access This Peer Review File is licensed under a Creative Commons Attribution 4.0 International License, which permits use, sharing, adaptation, distribution and reproduction in any medium or format, as long as you give appropriate credit to the original author(s) and the source, provide a link to the Creative Commons license, and indicate if changes were made.
In cases where reviewers are anonymous, credit should be given to 'Anonymous Referee' and the source.
The images or other third party material in this Peer Review File are included in the article's Creative Commons license, unless indicated otherwise in a credit line to the material. If material is not included in the article's Creative Commons license and your intended use is not permitted by statutory regulation or exceeds the permitted use, you will need to obtain permission directly from the copyright holder.
To view a copy of this license, visit https://creativecommons.org/licenses/by/4.0/

Revision for the manuscript "Intrinsic plasmon canalization in the biaxial van der Waals crystal $MoOCl_2$".

Reviewer comments are reported in black, our responses in blue, and the corresponding changes in the manuscript are highlighted in red.

Reviewer #1:

Intrinsic plasmon canalization in the biaxial van der Waals crystal MoOCl2 By Farid Aghashirinov and co-authors. This an interesting and timely work. Experiments are carefully performed. The manuscript is easy to read and easy to follow. Experimental results are well illustrated. In my opinion, this article will be of interest for a broader readership of Nature Nanotechnology.

Our answer:

We thank the reviewer for their positive and encouraging assessment of our work. We appreciate their recognition of the timeliness of the study, the clarity of presentation, and the quality of the experimental results. We are grateful for their support and for noting the potential interest of our manuscript to the broader Nature Nanotechnology readership.

Reviewer #2:

The manuscript by Farid Aghashirinov et al. reports an intriguing observation of intrinsic plasmon-polariton canalization in $MoOCl_2$, supported by near-field imaging, numerical simulations, and analytical calculations. In particular, the authors demonstrate a thickness-dependent plasmon canalization frequency. The concept is of clear interest to the nanophotonics community.

Our answer:

We thank the reviewer for their thoughtful and positive evaluation of our manuscript. We particularly appreciate their recognition of the thickness-dependent tuning of the plasmon canalization frequency, as well as the significance of our observation of intrinsic plasmon–polariton canalization in $MoOCl_2$. We are grateful for their acknowledgment of the combined experimental and theoretical approach and for noting the relevance of this concept to the nanophotonics community.

However, in its current form the manuscript lacks several further characterizations and clarifications that are necessary to justify publication in Nature Nanotechnology. A substantial revision addressing the points below is required.

Reviewer #2:

1. The paper demonstrates directional confinement visually but does not report derived, quantitative metrics such as the propagation length as a function of wavelength within the canalization window.

Our answer:

We thank the reviewer for this important suggestion. In response, we have added a new section in the Supplementary Information where we report the experimentally extracted propagation length as a function of wavelength:

Action taken: We added the following new section in the SI:

Propagation length of anisotropic plasmon polaritons

To quantify the propagation length of mid-IR polaritons in $MoOCl_2$, we extract decay profiles along the propagation direction for the measurements taken on the 45 nm flake at different wavelengths. We investigate launching from both a disk antenna (Fig. 25S) and a rod antenna (Fig 26S).

In panel a of each figure, we report the near-field s-SNOM phase maps at different wavelengths. The white arrow indicates the direction along which the polariton profiles are extracted. Panel b shows the corresponding line profiles (offset for clarity), together with fits (black lines) using a decaying exponential function $f(x) = exp(-\alpha x)cos(kx + \phi) + C$. From these fits, we extract both the polariton wavevector k and the propagation length $L_p = 1/\alpha$.

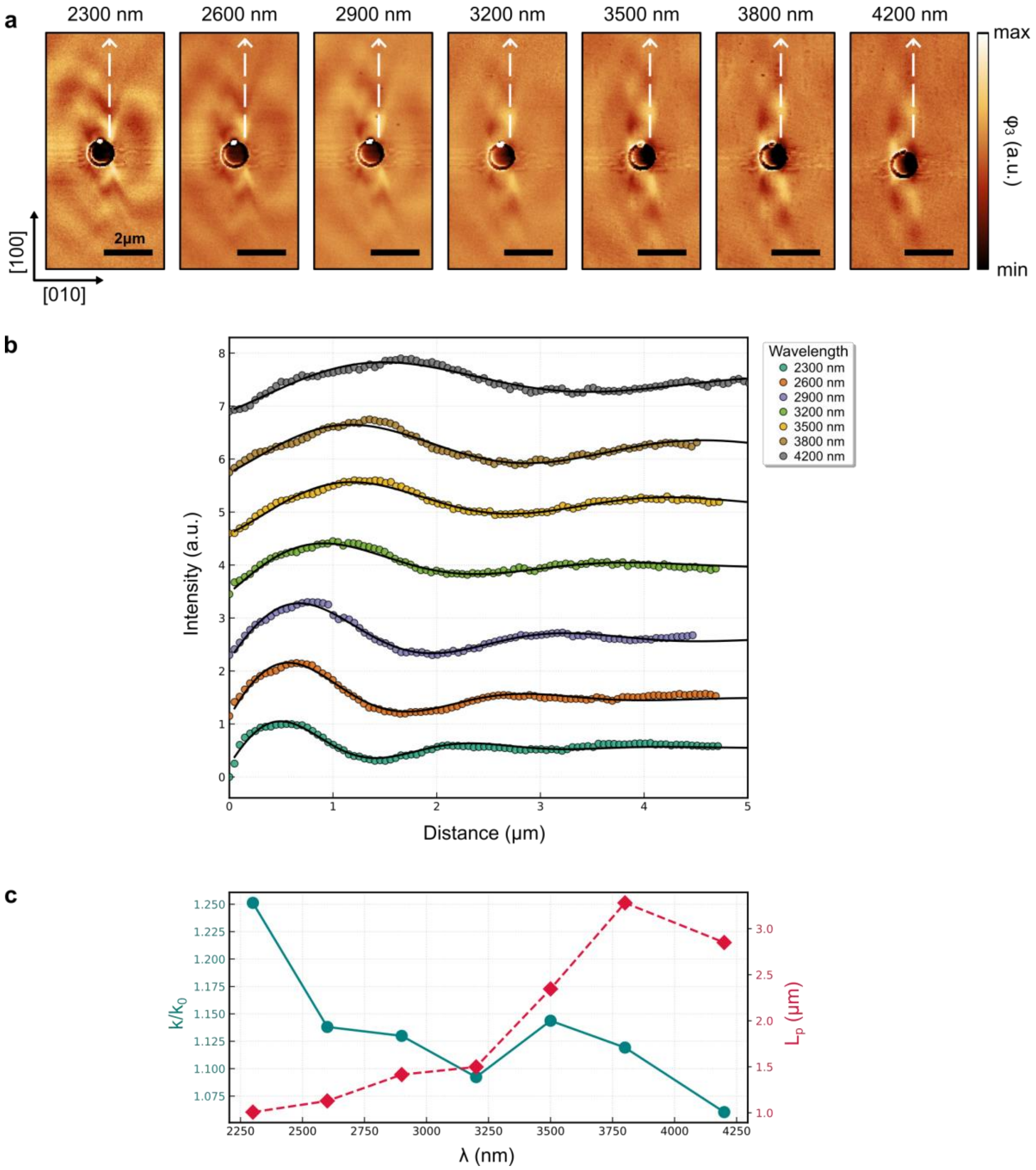

Figure 25S. Polariton propagation length on a 45 nm flake launched by a disk antenna. a sSNOM phase maps from which decay profiles are extracted along the white dashed lines. **b** Extracted profiles fitted with an exponentially decaying function. Curves are offset for clarity. **c** Retrieved wavevector and propagation length as a function of wavelength.

The results are summarized in panel c, where we plot the extracted wavevector normalized to the free-space value, k/k_0, and the propagation length as a function of wavelength. We observe a decrease in the polariton wavevector accompanied by an increase in the propagation length as the wavelength increases.

This trend is expected, as higher confinement (larger k/k_0) is generally associated with stronger losses and thus shorter propagation distances. The analysis is performed up to wavelengths until the canalization condition, as beyond this wavelength polaritons become strongly damped, making it difficult to reliably extract a propagation length from the experimental data.

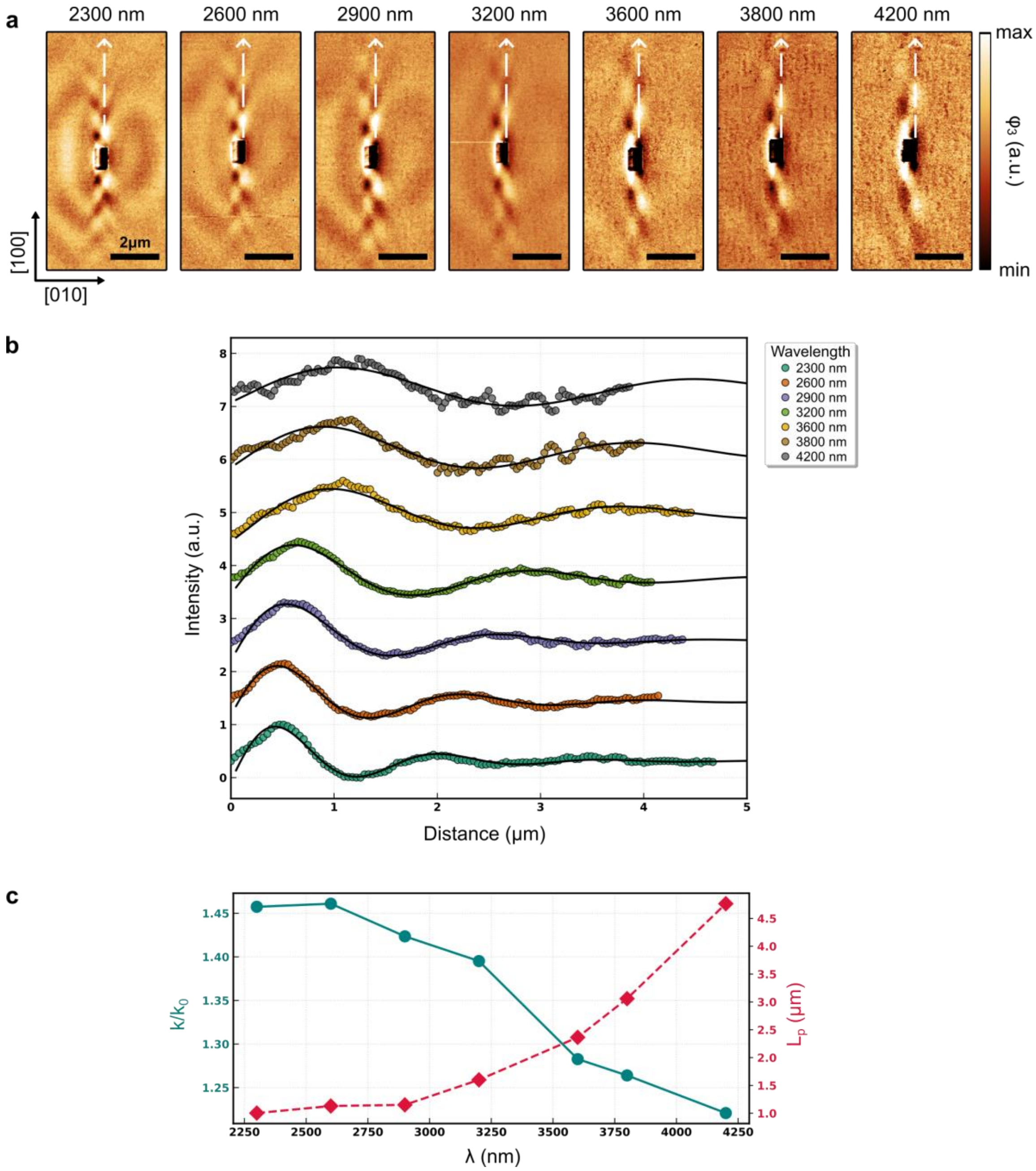

Figure 26S. Polariton propagation length on a 45 nm flake launched by a rod antenna. a sSNOM phase maps from which decay profiles are extracted along the white dashed lines. **b** Extracted profiles fitted with an exponentially decaying function. Curves are offset for clarity. **c** Retrieved wavevector and propagation length as a function of wavelength.

The increased damping observed near the canalization frequency can be attributed to the vanishing of the ε_y component, which leads to enhanced electric fields in the material. As a result, even moderate intrinsic losses are effectively amplified, yielding a reduction of the propagation length in the vicinity of the canalization condition.

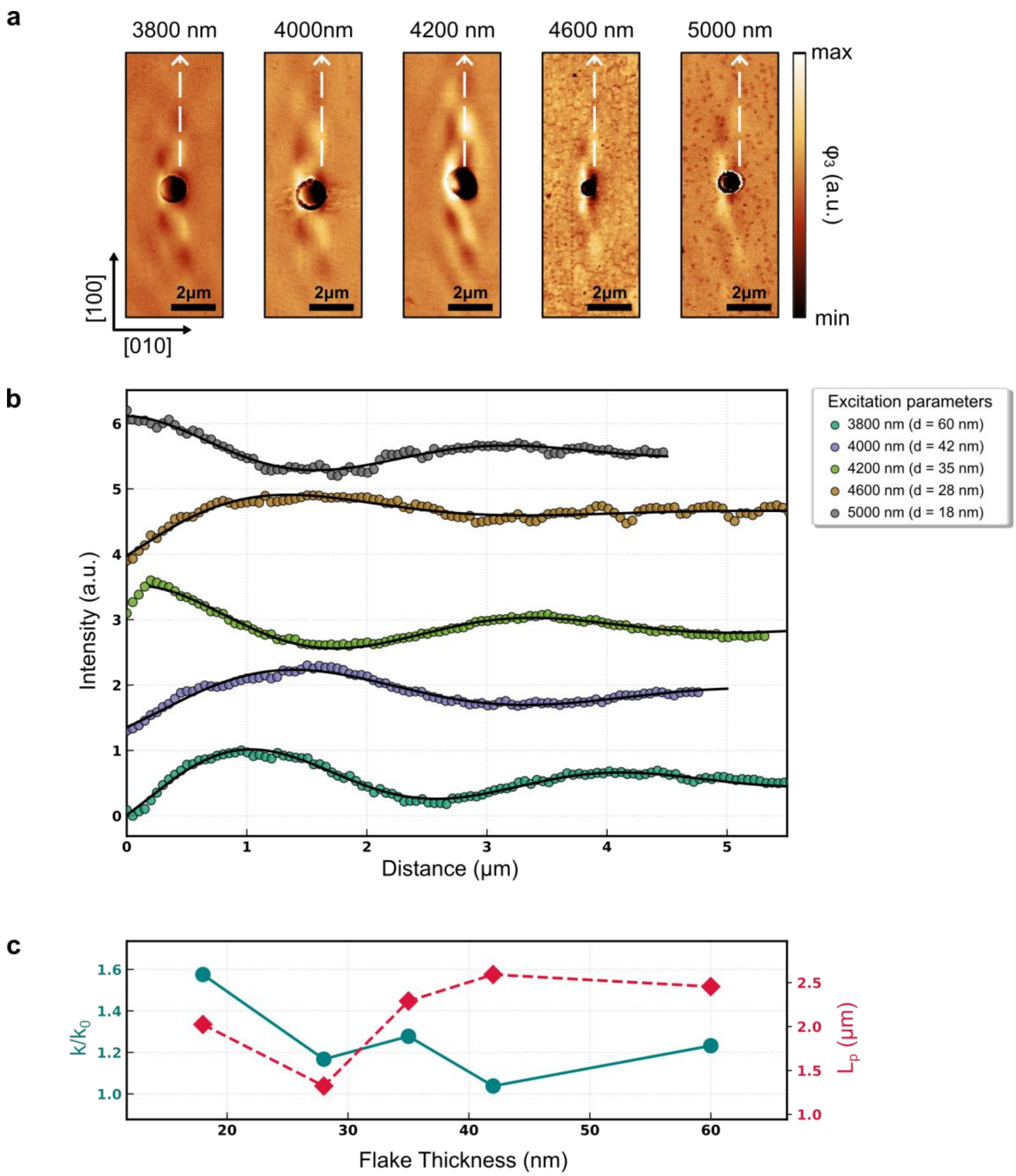

Figure 27S. **Polariton propagation length at different film thicknesses. a** sSNOM phase maps from which decay profiles are extracted along the white dashed lines. Each map is taken at a different wavelength (just before the canalization condition) and corresponds to a different flake thickness (in order of decreasing thickness, with same values as in Fig. 12S. **b** Extracted profiles fitted with an exponentially decaying function. Curves are offset for clarity. **c** Retrieved wavevector and propagation length for the different flake thicknesses (and thus different canalization frequencies).

To further investigate the evolution of the polariton propagation, we performed a thickness-dependent analysis of the polariton propagation length at wavelengths just below the canalization condition where it is easier to extract the propagation length (Fig. 27S). We observe that decreasing the flake thickness (thus redshifting the canalization frequency) is accompanied by an increase in the polariton momentum and a corresponding decrease in the propagation length. This behavior is consistent with the fact that achieving canalization at longer wavelengths requires thinner flakes, which results in stronger confinement of the polariton modes. As expected, increased confinement leads to enhanced losses and therefore shorter propagation distances.

Additional remark:

We would like to clarify that, beyond the visual observation of the canalization condition, the manuscript already includes a quantitative analysis of the polariton dispersion through the extraction of the vertex curvature from the experimental 2D FFT maps. This approach enables us to track the evolution of the isofrequency contours as a function of wavelength and to precisely identify the canalization condition via the curvature zero-crossing. To the best of our knowledge, this represents the first experimental implementation of such a method to quantitatively follow the isofrequency contours evolution and precisely determine the canalization frequency in anisotropic polariton systems.

Reviewer #2:

2. The authors state that "we determine the zero crossing point by fitting the experimental curvature vs wavelength data with a third-order polynomial." The rationale for using a third-order polynomial should be explained.

Our answer:

We thank the reviewer for this helpful comment. The precise analytical dependence of the isofrequency contours curvature on wavelength is not known in closed form because the thin-film polariton dispersion is governed by the implicit equation 8 of the SI. Although the isofrequency contours can be obtained numerically from this equation at each wavelength, extracting a simple explicit expression for the curvature at the vertex is not straightforward, as the equation is implicit and contains nonlinear dependences on the dielectric tensor components, film thickness, and surrounding media.

We therefore used a third-order polynomial as a phenomenological interpolation of the experimentally extracted curvature-wavelength data. This form provides sufficient flexibility to capture the smooth curvature evolution and zero-crossing behaviour across all measured thicknesses. We now clarify this point in the SI:

Action taken: We added the following paragraph to the SI:

To fully exploit the extracted dataset, we determine the zero-crossing point by fitting the experimental curvature versus wavelength dependence with a third-order polynomial. This phenomenological approach is adopted because the curvature cannot be expressed in closed analytical form as a function of wavelength, given that the isofrequency contours are governed by an implicit dispersion relation (see eq. 8 below).

Additional action taken: We added the following paragraph to the main text:
From measurements taken on a wide range of wavelengths we can produce a curvature vs. wavelength curve (Fig. 3d), which we fit with a third-order polynomial to extract the zero crossing point, representing our retrieved canalization wavelength (see Supplementary Information 3).

Reviewer #2:

3. I suggest the authors state, in the Abstract and Introduction, the physical reason why film thickness controls the canalization frequency. In the Conclusion, discuss whether this thickness dependence is expected to be universal for other biaxial or low-symmetry van der Waals crystals and under what conditions.

Our answer:

We thank the reviewer for this helpful suggestion. While revising the argument for the canalization frequency shift with flake thickness we realized our argument was too unpolished and did not stand further inquiry. We revise here the principle underlying the topological transition shift. First of all, we can consider the simplest case: a material with constant dielectric function ($\varepsilon_x = -500 + 100i$; $\varepsilon_y = -2 + 0.1i$) showing canalization at 5500 nm at a thickness of 35 nm. In this section we restrict ourselves to the case of a layer in vacuum to avoid any effect that could be related to the substrate refractive index. The evolution of the isofrequency contours calculated from the transfer matrix is shown in **Figure R1**. A transition from hyperbolic (at low thickness) to elliptic (high thickness) can be seen **even with a constant dielectric function**. This necessarily means that the frequency shift is not driven only by a dispersive dielectric function, but also by the relative change between the free-space momentum and

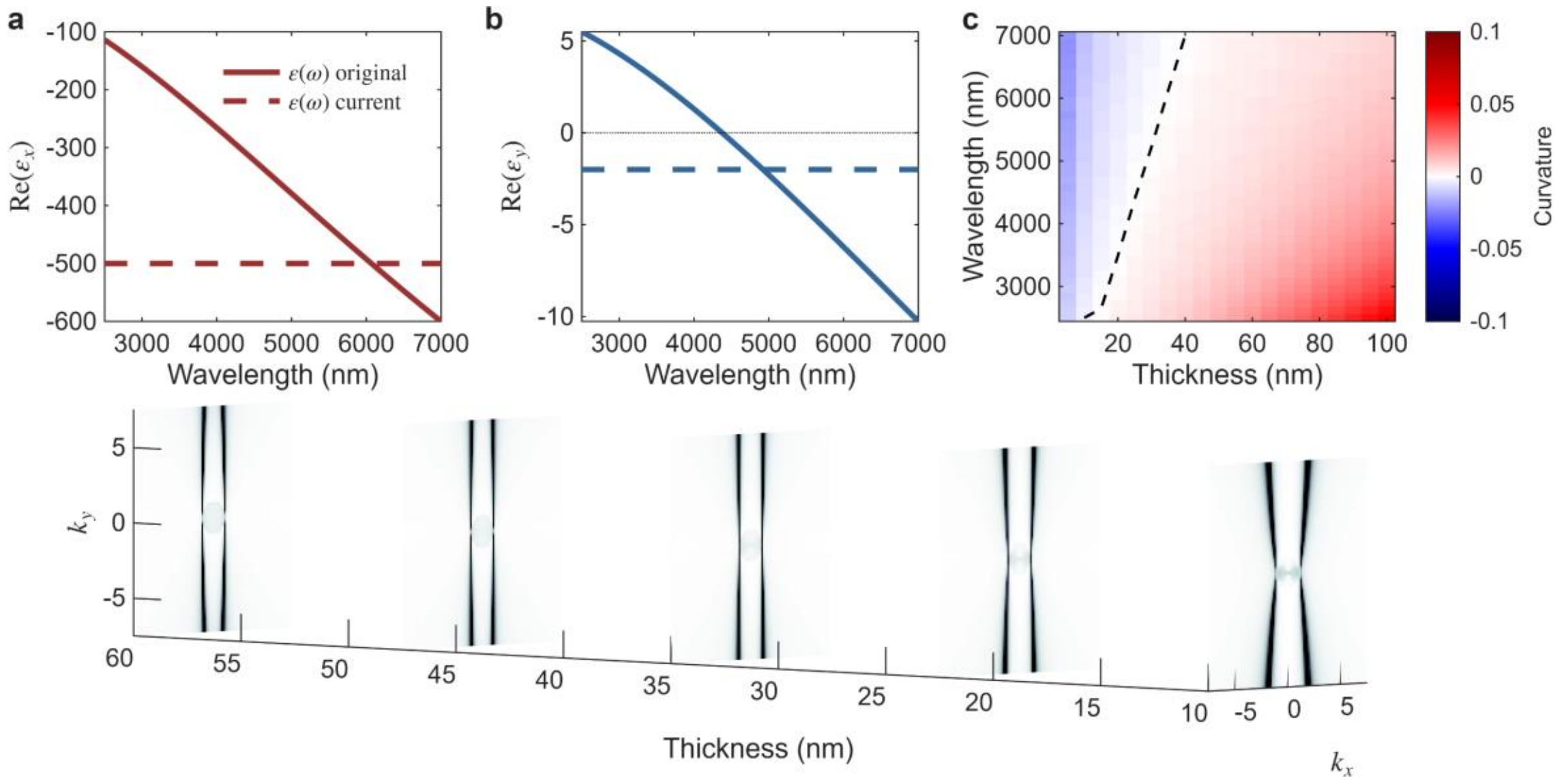

Figure R1. Canalization shift for a material with constant dielectric function (dashed lines, $\varepsilon_x = -500 + 100i$; $\varepsilon_y = -2 + 0.1i$). The used permittivity is shown along the MoOCl2 mid IR dielectric function along the **a** x and **b** y directions. **c** Shift of the canalization frequency computed from the analytical dispersion as inferred from the zero of the isofrequency contours vertex curvature. **d** Isofrequency contours calculated from the transfer matrix at 5500 nm for flakes of different thicknesses.

the flake thickness (here fixed). Indeed, by looking at eq. 8 of the SI (dispersion for film much thinner than the wavelength), the thickness always appears multiplied by k_0 in all terms. This means that, regardless of the specific isofrequency contours shape, changing the thickness by a factor xd can be compensated by varying at the same time the free space momentum k_0/x. This basically means that an **increase** in thickness leads to an **increase** in the canalization wavelength in a linear way. This is opposite to what we observe in the experiments and simulations, meaning that the Drude dispersion must overcompensate this effect to produce the observed behaviour (**increased** thickness leads to a **decreased** canalization wavelength in the experiments). As the terms containing the dielectric function are of the type $d\varepsilon(\lambda_0)/\lambda_0$, it is not surprising that a dielectric function that has a decreasing value (negative, so increasing absolute value) at longer wavelengths can compensate the trend from λ_0. From the analytical calculation of the isofrequency contours evolution, we can confirm that indeed a constant dielectric function leads to a linear shift of the canalization wavelength with flake thickness (Fig. R1c).

To better understand the role played by the dispersive nature of the permittivity along the two different directions, we can analyse what happens when one axis permittivity is kept constant while the other is dispersive. We first keep the y ([010]) direction to be $\varepsilon_y = -2 + 0.1i$, while we use the permittivity of $MoOCl_2$ for the x ([100])-direction (**Figure R2**). The resulting isofrequency contours are almost unchanged compared to the previous case (Fig. R1) of constant dielectric function, indicating that the dispersion along the x-direction does not influence much the shift of the canalization frequency.

Tracking the shift of the canalization wavelength through the analytical isofrequency contours also indicates that the trend is very similar to the one of constant permittivity as the canalization wavelength scales linearly with the flake thickness. The small role played by ε_x in this range is to be expected as the permittivity is very negative and the material closely resembles a perfect electric conductor. Thus, we can conclude that the dispersion along x is not the one responsible for the observed (inverted) canalization frequency shift.

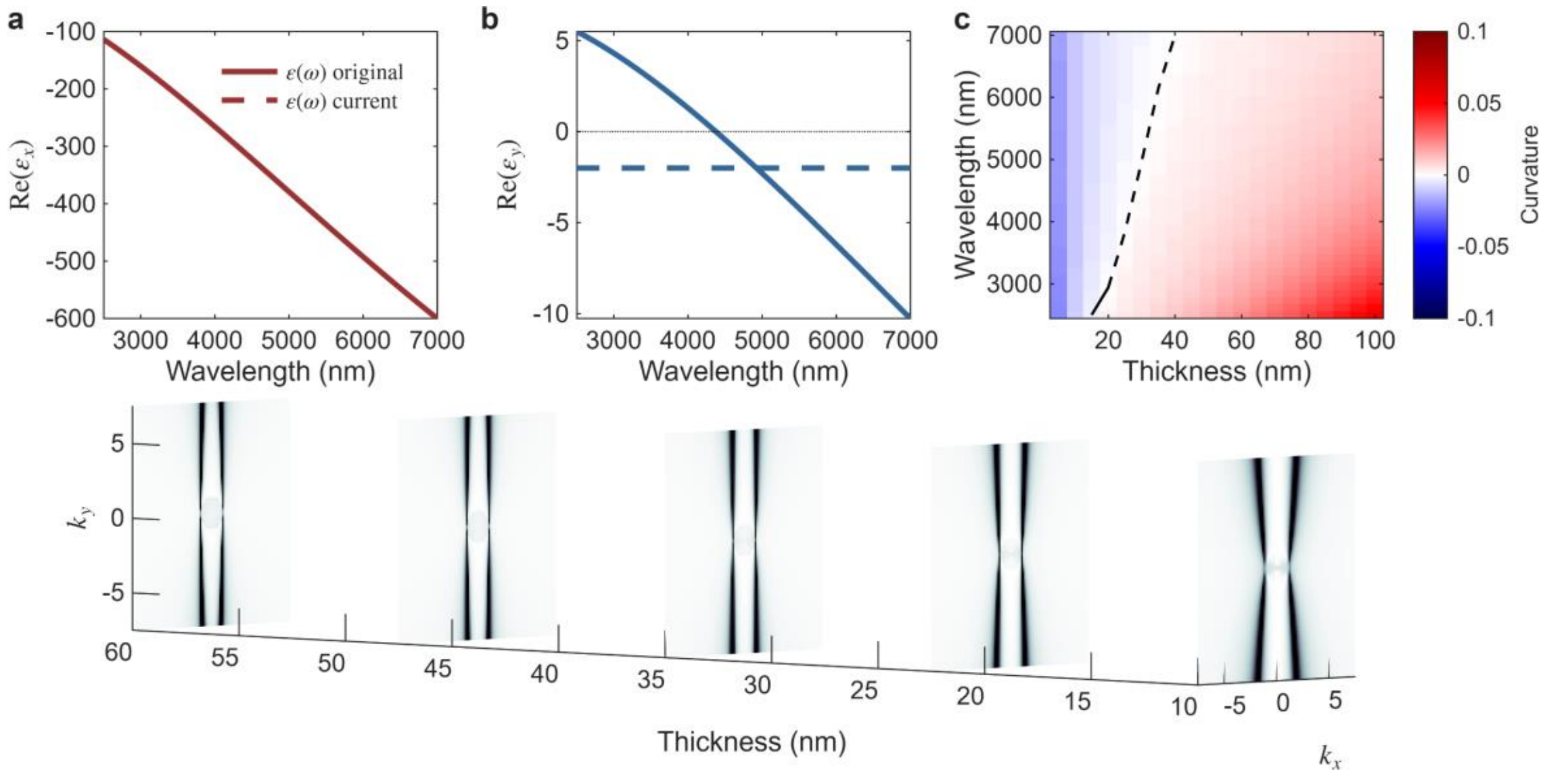

Figure R2. Canalization shift for a material with the mid-IR dielectric function of MoOCl2 along x and a constant permittivity along y (dashed lines $\varepsilon_y = -2 + 0.1i$). The used permittivity is shown along the MoOCl2 mid IR dielectric function along the **a** x and **b** y directions. **c** Shift of the canalization frequency computed from the analytical dispersion as inferred from the zero of the isofrequency contour vertex curvature. **d** Isofrequency contours calculated from the transfer matrix at 5500 nm for flakes of different thicknesses.

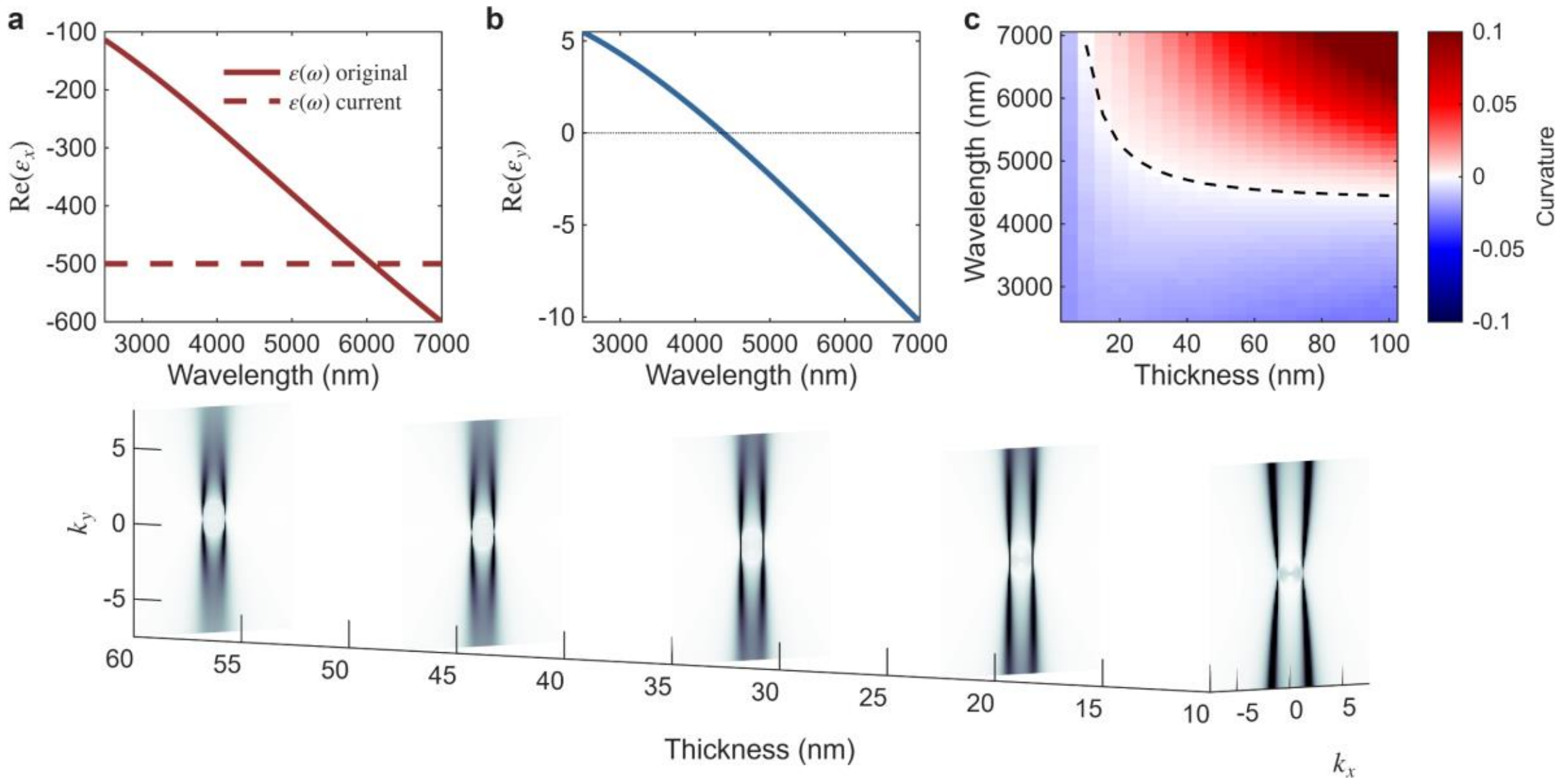

Figure R3. Canalization shift for a material with the mid-IR dielectric function of MoOCl2 along y and a constant permittivity along x (dashed lines $\varepsilon_x = -500 + 100i$). The used permittivity is shown along the MoOCl2 mid IR dielectric function along the **a** x and **b** y directions. **c** Shift of the canalization frequency computed from the analytical dispersion as inferred from the zero of the isofrequency contour vertex curvature. **d** Isofrequency contours calculated from the transfer matrix at 5500 nm for flakes of different thicknesses.

At the same time, we can investigate the opposite case, where $\varepsilon_x = -500 + 100i$ is kept constant while the permittivity along y is the one of $MoOCl_2$ in the mid-IR (**Figure R3**). The isofrequency contours exhibit a much quicker transition towards the elliptical regime at high flake thicknesses, signalling a different qualitative trend compared to the previous ones. Indeed, tracking the calculated thickness induced shift of the canalization wavelength yields results that are very similar to the one experimentally obtained. This observation confirms that the shift of the canalization wavelength is mainly driven by the permittivity dispersion along the y direction, while the dispersion in x plays a secondary role with only a small quantitative difference from the constant (very negative permittivity) case.

To assess whether thickness-dependent tuning of the canalization wavelength is expected in other van der Waals systems, we considered a representative Lorentz–Lorentz dielectric response corresponding to the typical case of phonon-polariton materials with Reststrahlen bands along both in-plane axes. As a model system, we started from the in-plane dielectric function of hBN and rescaled the phonon frequencies such that the longitudinal optical phonon, and therefore the relevant sign-changing region of the permittivity, lies near 4.2 µm, comparable to the intrinsic canalization wavelength of $MoOCl_2$.The resulting dielectric functions and corresponding isofrequency contours evolution are plotted for three different anisotropy values (scaling = 1.05, 1.10 and 1.15, applied as a multiplicative factor to ω_{LO}, ω_{TO} and the damping γ) in **Figure R4**. For each case, the upper panel reports the real part of the dielectric function along the two in-plane axes. A dashed gray line marks the wavelength of 4200 nm, corresponding to the zero-crossing of the y-axis permittivity. The dielectric response along the x direction is shifted toward shorter wavelengths, reproducing a situation analogous to $MoOCl_2$ where the two axes undergo the sign transition at different spectral positions.

The lower panels depict the corresponding isofrequency contours calculated via the transfer-matrix method in the wavelength range between 4400 nm and 4000 nm for a representative 50 nm film. As the separation between the two Reststrahlen bands increases, the confinement of the modes decreases (because ε_x becomes more negative) together with the mode losses due to smaller field penetration. In all cases, we observe the intrinsic topological transition between elliptical to hyperbolic propagation in the 4300-4200 nm range. The final row tracks the evolution of the canalization wavelength for the three anisotropy values by calculating the analytical isofrequency contours and extracting the curvature through the same conic fitting procedure employed in the main text. In all cases, the canalization wavelength remains confined within the relatively narrow 4300–4200 nm spectral range and exhibits only a weak dependence on thickness. This behavior contrasts strongly with $MoOCl_2$, where over the same thickness range the canalization wavelength can be tuned by more than 1 µm. These results highlight how a Drude–Drude dielectric response is significantly more favourable than a Lorentz–Lorentz one for achieving intrinsically broadband and thickness-tunable canalization.

Actions taken:

Section 5 (previous section 4) of the SI has been completely reworked to include this analysis of the canalization wavelength shift origin and how it is related to the material in-plane dielectric function.

For the sake of brevity, in the Abstract we simply mention that the slope of the Drude term is what allows broadband canalization and thickness-tuning:

Owing to the moderate slope of the Drude permittivity, the resulting polaritons remain highly directional across a broad spectral window. This weak dispersion also enables robust thickness-dependent tuning, and we demonstrate, both experimentally and theoretically, that the canalization wavelength can be adjusted by more than 1 µm simply by varying the flake thickness.

At the end of the introduction we now added:

In contrast to conventional phonon-polaritonic platforms governed by Lorentz-type dispersions, the Drude-like response of $MoOCl_2$ enables broadband canalization over an extended spectral range while simultaneously providing efficient thickness-dependent tunability. The observed shift of the canalization wavelength is primarily driven by the dispersion along the [010] axis, whereas the strongly metallic response along [100] remains deeply negative and therefore only weakly influences the evolution of the isofrequency contours curvature with thickness.

In the description of Fig. 3 of the main text we now write:

By looking at the equation for the theoretical isofrequency contours (Supplementary Information 5), additional insight into the thickness dependence of the canalization condition can be obtained. Assuming a frequency-independent dielectric response, with one in-plane permittivity component remaining deeply negative and the other negative but closer to zero, the canalization wavelength is expected to increase linearly with thickness. This follows from the analytical expression, where the slab thickness and free-space wavevector appear only through their product, so that the canalization condition is preserved by scaling thickness and wavelength by the same factor (see Supplementary Information 5). In $MoOCl_2$, however, the opposite trend is observed experimentally and theoretically: increasing the flake thickness leads to a reduction of the canalization wavelength. This behavior is mainly driven by the dispersion of the [010] permittivity, which governs the evolution of the isofrequency contours shape across the topological transition. Since the permittivity enters the canalization condition multiplied by the flake thickness d, an increase in the absolute value of ε_y (i.e., a more negative response) requires a smaller thickness to satisfy the condition. As a result, the thickness dependence of the canalization wavelength qualitatively follows the Drude-like dispersion of the [010] axis, explaining the trend reported in Fig. 3e. In contrast, the permittivity along the [100] direction remains strongly negative throughout the relevant spectral range and therefore behaves almost as a perfect electric conductor, contributing only weakly to the thickness-induced shift of the canalization condition.

In the concluding part of the discussion we now also comment on the possibility of tuning the intrinsic canalization wavelength via flake thickness in a model Lorentz-Lorentz material:

From sSNOM experiments on flakes between 18 nm and 60 nm, we obtain the experimental data plotted as dots in Fig. 3e, confirming that the canalization wavelength can be tuned over more than 1 µm, corresponding to a variation > 25% of the central wavelength by adjusting the flake thickness. We also show that in a model Lorentz-Lorentz phonon-polariton material, canalization naturally emerges only within a narrow spectral. As a consequence, the achievable thickness-dependent tuning remains intrinsically limited by the restricted bandwidth over which canalization exists (see Supplementary Information 5). In contrast, the Drude-like response of MoOCl2 enables canalization across a much broader spectral range, allowing substantially larger shifts of the canalization wavelength with thickness. Importantly, such a high tuning bandwidth goes well beyond what has been achieved in phonon-polariton systems, where tuning via twist angle in bilayer [34–36] and trilayer [38] heterostructures or by substrate engineering [41, 42] was constrained to < 9% of the central wavelength. Larger tunability has been reported for PhPs at THz frequencies [37], yet it is still limited to less than 14% of the central energy.

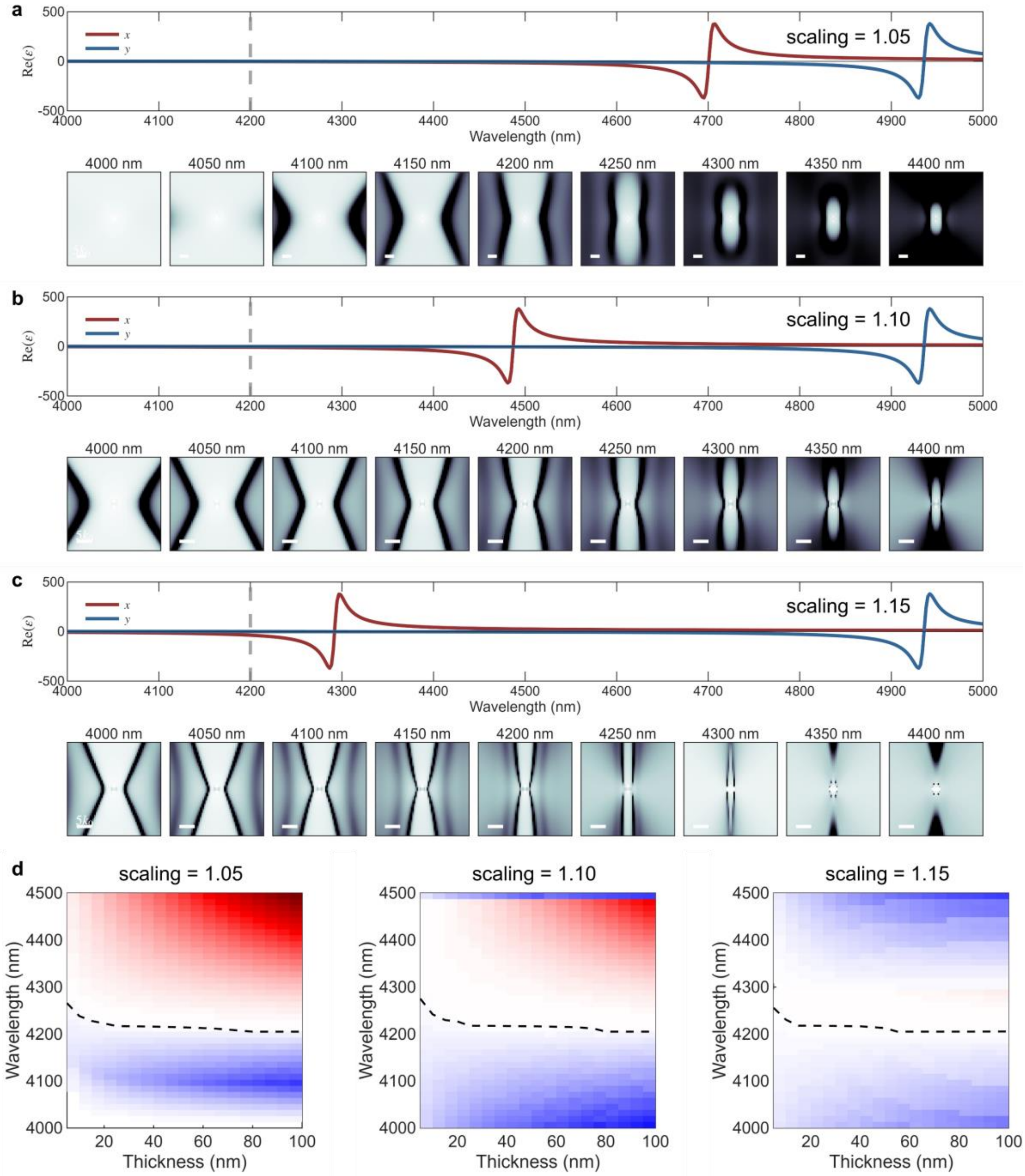

Figure R4. **Canalization wavelength shift in a model Lorentz–Lorentz van der Waals polaritonic system**. Real part of the in-plane dielectric function, with the dashed gray line marking the 4200 nm wavelength where the y-axis permittivity crosses zero. Each permittivity is characterized by the scaling between the two Lorentz oscillators with values of **a** 1.05, **b** 1.10 and **c** 1.15. For each permittivity, the corresponding isofrequency contours calculated via transfer-matrix methods in the 4000–4500 nm spectral range are shown. **d** Extracted canalization wavelength as a function of thickness, obtained from analytical isofrequency contour calculations and conic fitting of the isofrequency contour curvature for all three permittivities.

Reviewer #2:

4. For figure 5S, please report reproducibility statistics: how reproducible is the canalization frequency (or curvature zero crossing) for flakes of nominally identical thickness? The current data show relatively large discreteness—explain the likely sources (thickness measurement uncertainty, flake-to-flake variability, local strain, orientation, defects, substrate effects, etc.).

Our answer:

We clarify that the statistical spread reported in Fig. 5S (now Fig. 12S and Fig. 3d) does not arise from measurements on different flakes of nominally identical thickness, but rather from multiple independent polariton excitations within the same flake. Specifically, the dataset includes measurements from single-disk launchers as well as from sets of four disks with different radii. For each launcher, the analysis is performed independently on fringes from both sides of the disk, resulting in multiple (at least two) determinations of the canalization condition.

The observed spread therefore mainly reflects variations associated with the excitation geometry (incident light direction and launcher asymmetry) and analysis procedure, rather than intrinsic flake-to-flake variability. In addition, since the disks probe different local regions of the same flake, small variations in the local environment, such as residual strain, defects, slight thickness inhomogeneities, or substrate-induced effects, may also contribute to the dispersion of the extracted canalization frequencies. In the SI section describing the Fig. 5S (now Fig. 12S) mentioned by the reviewer we now added:

Action taken: We added the following paragraph to SI:

Gray dots are the single curvature values used to obtain the average (red dots), while black dashed curves are the polynomial fits. The spread of the individual data points mainly reflects variations associated with the excitation geometry and independent analysis of different launcher regions, although small local variations of the flake properties (e.g., residual strain, defects, or thickness inhomogeneities) may also contribute.

Reviewer #2:

5. For figure 1S, the authors should show detailed fitting components for representative samples. Additionally, provide a sensitivity analysis: how do uncertainties in the fitted ε(ω) parameters affect the predicted isofrequency contours shapes and the canalization condition?

Our answer:

We thank the reviewer for this suggestion. Upon closer inspection, we identified an inconsistency between the dielectric function parameters reported in the table and those used to generate the fitted curves shown in the figure and the whole theoretical analysis. We have corrected this issue in the revised manuscript (see updated table below).

In addition, by explicitly analysing the individual contributions from the Drude and Lorentz components, we found that the inclusion of Lorentz oscillators in the [100]-direction was not necessary, as the Lorentz peak was located at much higher energies than the ones investigated here (but was reauired to model the optical response of $MoOCl_2$ in the visible in our previous work [10.1021/acsnano.5c07323]). The [100] dielectric function can be accurately reproduced using a single Drude term, allowing us to reduce the number of fitting parameters and reduce overparameterization. Permittivity parameters in the table have been updated accordingly.

Axis	ε_∞	ω_p	γ	$\omega_{0,1}$	$\omega_{p,1}$	γ_1
[100]	0	5.63	0.15	0	0	0
[010]	7.85	0.91	0.11	1.15	1.30	1.99

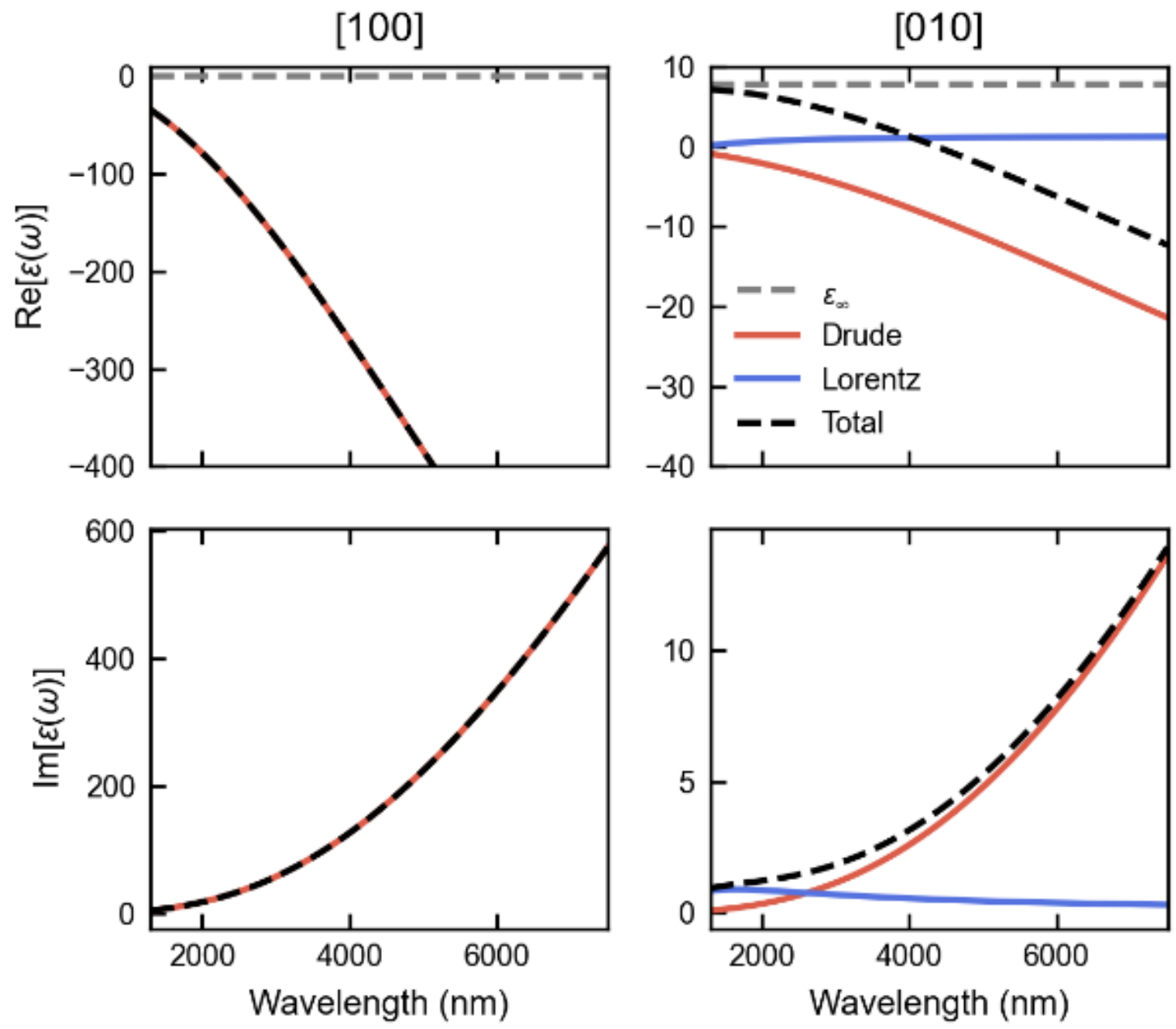

Figure 4S: Mid-IR dielectric function decomposition. Contribution from the Drude and Lorentz terms to the extracted dielectric function of $MoOCl_2$ in the IR range.

Following the referee suggestion we also added a new figure plotting the decomposition of the final fitted permittivity into its individual contributions. While the [100] direction can be described by a single Drude contribution, the [010] one needs the inclusion of a Lorentz term to account for reduced transmission at lower wavelengths. In the SI we added the following:

Action taken: We added the following paragraph to SI:

The decomposition of the extracted dielectric function into its constituent contributions (Fig. 4S) highlights the distinct physical origins of the optical response along the two crystallographic directions. In the [100]-direction, the permittivity is well described by a single Drude term, indicating a predominantly free-carrier response with no significant interband contributions in the spectral range of interest. In contrast, the [010]-direction requires the inclusion of an additional Lorentz oscillator to accurately capture the low transmission at lower wavelengths in the investigated range.

Additional answer:

Moreover, we have performed a sensitivity analysis of the dielectric function fitting. For the [100]-direction, the real part of ε_x is strongly negative over the spectral range of interest, so the response effectively approaches that of a perfect electric conductor and small changes in the fitted values do not appreciably change the polariton isofrequency contours. Moreover, the fit is well constrained by a single Drude term with limited parameter freedom. In contrast, the y-direction exhibits a greater degree of

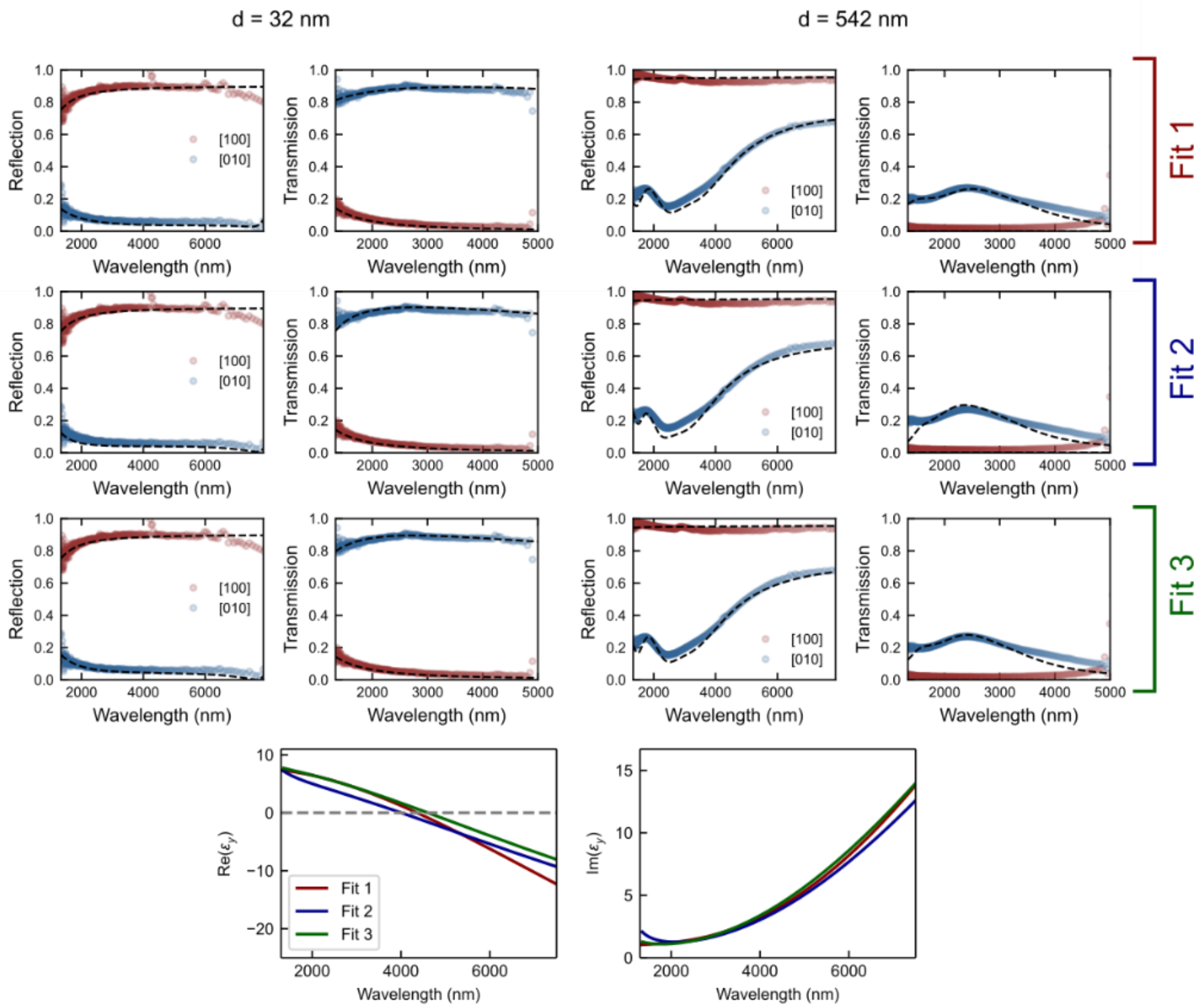

Figure 5S: Sensitivity of the [010] dielectric function fitting. Reflection and transmission spectra along the [100] and [010] directions for two representative flake thicknesses (32 and 542 nm) are shown together with three fits obtained from different starting parameters. Bottom panels show the corresponding extracted real and imaginary parts (obtained from the average of all the flakes thicknesses as in Fig. 1S) of ε along the [010] axis, illustrating the variability arising from fitting degeneracy.

flexibility in the fitting. Different combinations of parameters can reproduce the reflection and transmission data with similar accuracy. We find that variations in the high-frequency permittivity ε_∞ can, to some extent, be compensated by a redistribution between the Drude and Lorentz contributions. To assess the impact of this degeneracy, we performed multiple fits (on the same spectra shown in Fig 1S) starting from different ε_∞ values and extracted the corresponding dielectric functions. In a new figure (Fig. 5S), we show representative fits to the reflection and transmission spectra for two flakes of different thicknesses. The corresponding extracted dielectric functions are reported in the lower panels, illustrating the range of variation arising from different fitting parameterizations. The parameters of the Drude-Lorentz model for the two additional fits are reported below:

Fit # ([010])	ε_∞	ω_p	γ	$\omega_{0,1}$	$\omega_{p,1}$	γ_1
2	4.32	0.84	0.14	1.13	1.49	0.35
3	6.99	0.88	0.14	1.29	1.49	0.93

Action taken: In the SI we added the following description:

We also performed a sensitivity analysis of the dielectric function fitting. For the [100] direction, Re(ε_x) is strongly negative in the spectral range of interest, so the response is effectively metallic and the fit is well constrained by a single Drude term, leading to negligible impact on the extracted isofrequency contours . In contrast, the [010] direction allows for greater flexibility, as different parameter combinations can reproduce the reflection and transmission data with comparable accuracy (Fig. 5S, Fit 1 corresponds to the one used in the manuscript with parameters reported above). In particular, variations in ε_∞ can be partially compensated by a redistribution between the Drude and Lorentz contributions. To assess this, we performed additional fits starting from different ε_∞ values and extracted the corresponding dielectric functions. The resulting variation is illustrated in the new figure, where representative fits and the corresponding extracted permittivities are shown for two flake thicknesses, with the associated fit parameters (for Fit #2 and #3) reported in Table 3.

Additional answer:

Now we consider the effect of these different dielectric-function fits on the polariton isofrequency contours and on the curvature-zero-crossing trend as a function of thickness. For each fit, we recalculated the isofrequency contours , extracted their curvature using the same conic fitting procedure employed in the main text, and determined the corresponding canalization wavelength.

This analysis shows that the qualitative behaviour is robust across the different parameterizations: all fits reproduce a thickness-dependent redshift of the canalization wavelength and the same hyperbolic-to-elliptical evolution of the isofrequency contours . However, the absolute position of the calculated canalization curve is sensitive to the fitted $\varepsilon_y(\omega)$, with Fit 3 producing a more pronounced redshift. Overall, the uncertainty in the dielectric-function fitting affects the precise spectral position of the calculated canalization wavelength, but not the main conclusion: intrinsic canalization persists and remains broadband and thickness-tunable over the experimentally observed range.

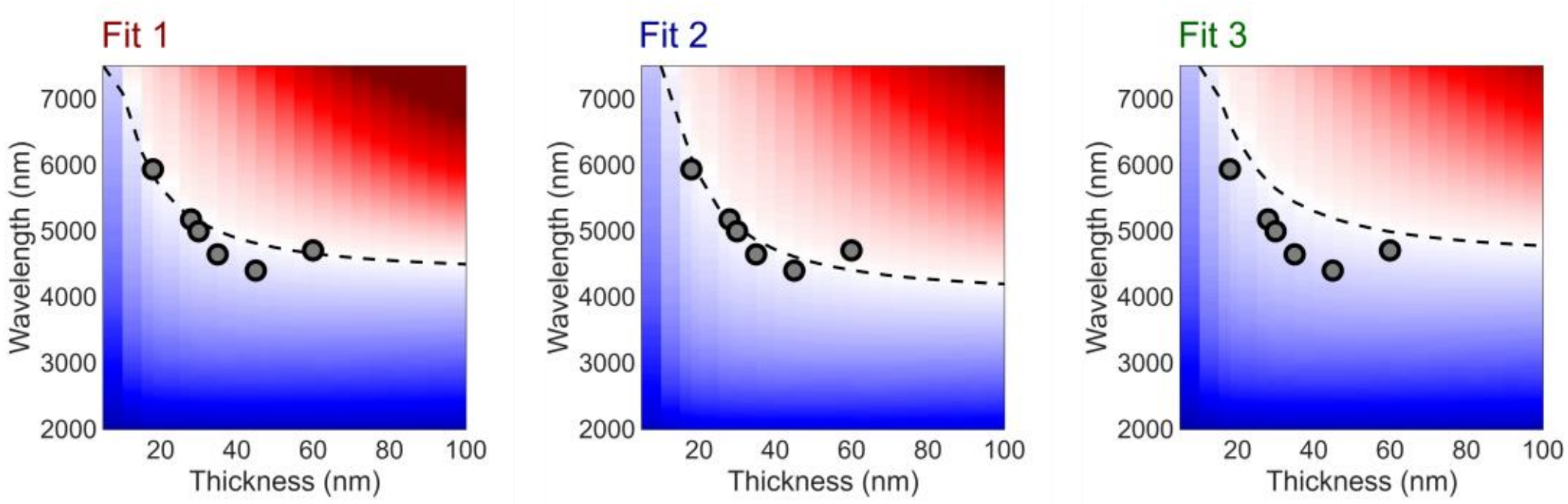

Figure 6S: Comparison of expected shift of the canalization wavelength with flake thickness for different fitted [010] permittivity. Isofrequency contours curvature evolution as a function of wavelength and thickness for the different fits shown in Fig. 5S.

Additional action taken: In the SI we added the following:

The sensitivity of the polariton isofrequency contours to the different dielectric-function fits is shown in Fig. 6S. For each fitted permittivity, we recalculated the isofrequency contours , extracted the curvature using the same conic fitting procedure employed in the main text, and determined the curvature zero-crossing wavelength as a function of thickness. The calculated curvature maps and zero-crossing lines show that the qualitative behavior is robust across the different parameterizations: all fits reproduce a thickness-dependent redshift of the canalization wavelength with a similar trend of the hyperbolic-to-elliptical evolution of the isofrequency contours . However, the absolute spectral position of the canalization curve depends on the fitted $\varepsilon_y(\omega)$, with Fit 3 producing a more pronounced redshift.

Thus, the uncertainty in the dielectric-function fitting affects the precise calculated canalization wavelength, but not the main conclusion that intrinsic canalization persists and remains thickness-tunable over the experimentally observed range.

Reviewer #3:

Aghashirinov et al. report on canalization at the topological transition between hyperbolic and elliptic polaritons in the van der Waals metal MoOCl2. They observe this transition in the mid-infrared around 4-5 microns, around the point of zero-crossing of the permittivity along the [010] direction. They show experimentally and in simulations that the canalization wavelength can be tuned by ~25% with the layer thickness of the MoOCl2 flake. Overall, the work is very impressive, timely, and well executed, not only by demonstrating canalization in a single layer of a natural crystal, but also because by opening up a new but very interesting wavelength range in the mid-infrared which thus far has been hardly accessible on van der Waals platforms. Thereby, the study certainly presents enough novelty to be published in Nature Nanotechnology.

Our answer:

We thank the reviewer for their detailed and highly positive assessment of our work. We appreciate their recognition of the thickness-dependent tuning of the canalization wavelength, enabled by the Drude response of the material. We are grateful for their acknowledgment of the timeliness, novelty, and experimental execution of our study, and for highlighting its relevance in opening a new mid-infrared regime for van der Waals nanophotonics.

Reviewer #3:

I do have a few (not too major) concerns I would ask the authors to address before I can recommend the publication of their manuscript:

1) The arguments for choosing this specific geometry with a rod-like launcher illuminated perpendicular to the propagation direction are a bit unclear. In explaining Fig. 2a,b, the authors argue that the efficiency for polarization along the long antenna axis (Fig. 2a) is less efficient than for along the short axis (Fig. 2b). This suggests that there is some antenna resonance involved in the excitation which in fact one would typically try to avoid in order not to distort the near-field propagation with the spatial profile of the antenna resonance. It is strange that a geometry that specifically requires polarization transformation (Px -> Py in Fig. 2b), in particular where the authors argue earlier in the text on page 6 that "the in-plane px polarization direction play a crucial role in the excitation efficiency of the plasmon polaritons". The use of this experimental geometry seems to directly contradict this statement since here the excitation specifically does NOT contain this crucial polarization component, which is only provided indirectly through the antenna.

To be clear, I do not criticize this approach necessarily, but solely the argument why this is used. On the plus side, the vertical phase flip of the mode due to this geometry may in fact be helpful in the analysis (it's not a bug, it's a feature!) due to more distinct features in the propagation pattern, and potentially the authors could raise this point.

As for the geometry, I would have expected the authors to rotate the antenna such that the long axis is parallel to the propagation direction which would, following their arguments, allow to excite canalized polaritons with high efficiency and with an incident polarization matched to the propagation. I would argue that the modification of fringe spacing from collinear illumination (for small confinements), as argued by the authors being a problem, is not really a problem but rather an opportunity to have two complementary (forward and backward) representations of the canalized mode in one image.

I would kindly ask the authors to either do that experiment (rotated antenna, collinear p-pol excitation), or at least compare the respective simulations, i.e., same as Fig. 2a,b but with additionally rotating the rod antenna by 90°. I would also be useful to show the antenna resonance effect in more detail that is argued to be responsible for the different efficiencies in Fig. 2a,b.

Our answer:

We thank the reviewer for the in-depth analysis of the launcher geometry and launching efficiency. To clarify the role of the excitation geometry and the associated launching efficiency, we have performed additional simulations where we systematically analyse the coupling between the incident field, the antenna geometry, and the resulting polariton excitation at the canalization frequency for a 45 nm thick layer. For simplicity and clarity we consider normal incidence excitation from a linearly polarized plane and investigate four configurations (scale bar of the E_z field is constant for all simulations for a fair visual comparison): polarization along the [100] and [010] directions, combined with rod antennas (modelled as perfect electric conducting elements with lengths from 450 nm to 2500 nm) elongated either along the [100] or [010] direction (while keeping the width fixed at 450). For each configuration, we extract the launched polariton profile via the simulated E_z field at the $MoOCl_2$ surface. We fit the spatial decay with a decaying oscillating exponential profile, allowing us to quantify the launching efficiency in a consistent manner by reporting the profile amplitude (see Figure R5 below).

We observe that when the rod is aligned along [010] and the incident polarization is along [100], the launching efficiency remains weak for all rod lengths. In contrast, when both the rod and the polarization are aligned along [100], the launching efficiency increases steadily with rod length, suggesting the onset of a resonance condition at longer antennas. As correctly guessed by the reviewer, the most efficient excitation is obtained when both the rod and the polarization are aligned along [010], where we observe a clear maximum around a rod length of ~1.1 µm, consistent with a resonance-like behaviour. Importantly, aside from the increase in intensity, we do not observe any significant distortion of the polariton propagation profile in these simulations, although we note that in experiments such effects could arise due to interactions with the s-SNOM tip. Finally, in the configuration employed in the experiments (rod along [100] and polarization along [010]), we find a robust and efficient excitation that is only slightly weaker than the previous case and remains largely independent of the rod length.

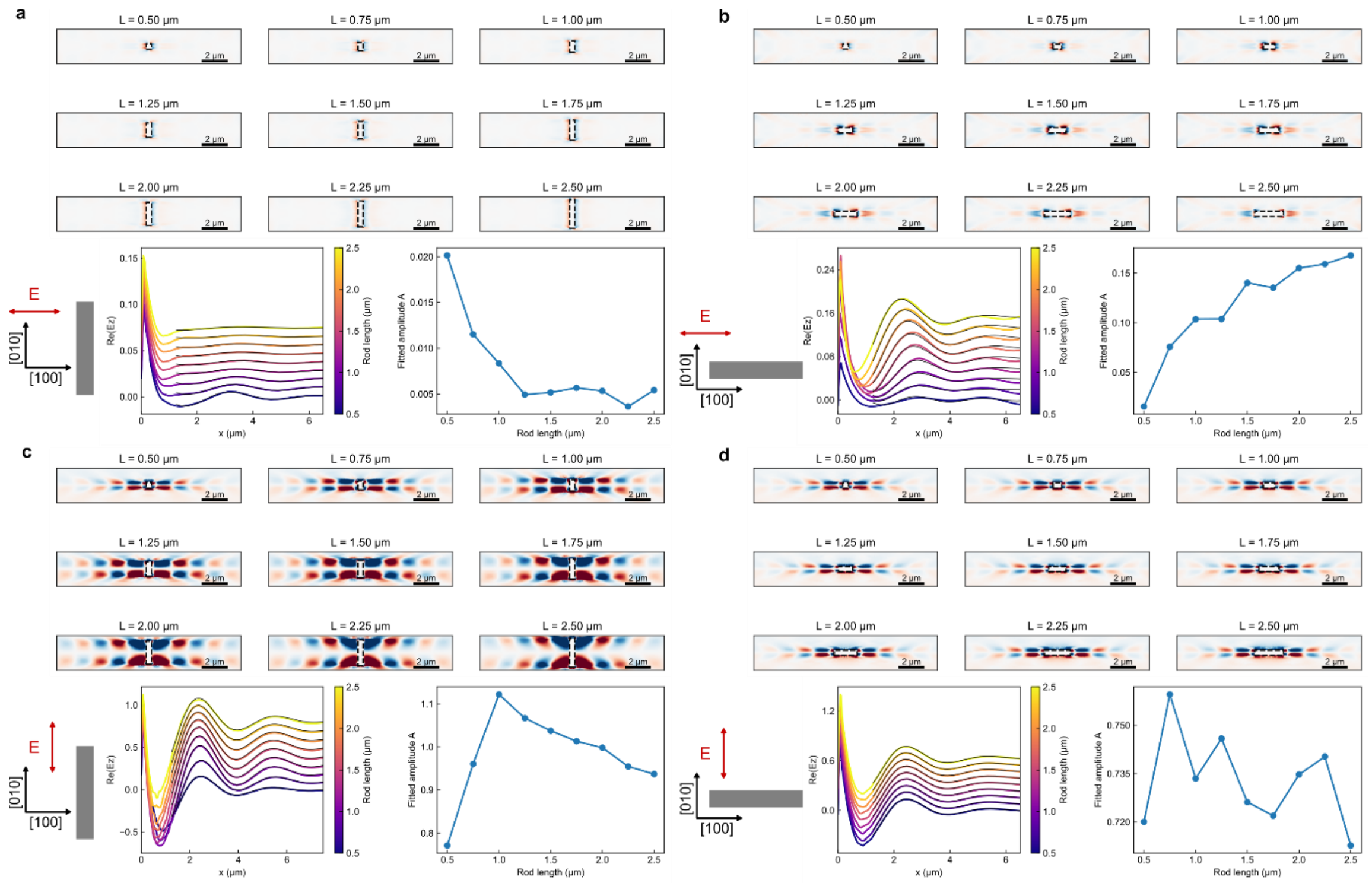

Figure R5. **Simulated polariton launching in a 45 nm flake at the canalization frequency**. Simulated E_z maps launched by a rod of various lengths and fixed width. The rods are aligned with the [010] direction in **a, c** and with the [100] direction in **b, d**. The polarization of the exciting field is along [100] in **a, b** and along [010] in **c, d**. Electric field profiles are fitted with an oscillating decay curve and the extracted amplitude is reported as a function of the rod length.

An intuitive understanding of why excitation along the [010] direction is more efficient for the launching of canalized polaritons propagating along the [100] direction can be extracted from the overlap of the polariton isofrequency contours and the momentum distribution of an in-plane dipole. If we imagine the dipole to be representing the effect of a small launcher excited by a plane wave, the dipole direction is then determined by the polarization of the excitation field. The electric field of a dipole with dipole moment $\boldsymbol{p}$ can be written as [Novotny, Hecht. *Principles of Nano-Optics*, 2012.]:

$$\boldsymbol{E}(\boldsymbol{r}) \propto \boldsymbol{G}(\boldsymbol{r}) \cdot \boldsymbol{p}$$

where the Dyadic Greens function is

$$\boldsymbol{G}(\boldsymbol{r}) = \left(\boldsymbol{I} - \frac{\nabla\nabla}{k_0^2}\right) G_0(r) \text{ with } G_0(r) = \frac{e^{ik_0 \cdot r}}{4\pi r}$$

In momentum space this can be rewritten as (using Weyl identity):

$$\boldsymbol{G}(\boldsymbol{k}) \propto \left(\boldsymbol{I} - \frac{\mathbf{kk}}{k_0^2}\right) G_0 \text{ with } G_0 \propto e^{ik_z|z_0|}/k_z$$

where z_0 is the vertical position of the dipole. As we are interested in the near-field interaction, we can consider for simplicity the large momentum limit $k_\parallel \gg k_0$ and $k_z = \sqrt{k_0^2 - k_\parallel^2} \sim ik_\parallel$, and only the second term is retained in $\boldsymbol{G}(\boldsymbol{k})$. The in-plane momentum dependency of the field is then proportional to:

$$E(\boldsymbol{k}_\parallel) = \boldsymbol{G}(\boldsymbol{k}_\parallel) \cdot \boldsymbol{p} \propto \boldsymbol{k}_\parallel \cdot \boldsymbol{p}$$

It follows that the power coupled at different momenta is:

$$\left|E(\boldsymbol{k}_{\|})\right|^2 \propto \left|\boldsymbol{k}_{\|} \cdot \boldsymbol{p}\right|^2 = (k_{\|} p \cos\phi)^2$$

By plotting the in-plane dipole emission over the isofrequency contours (Fig. 9S) it is straightforward to observe that a dipole oriented along the [010] direction is better coupled to the isofrequency contours at the canalization condition. Conversely, in the hyperbolic case a dipole oriented in the [100] direction shows better overlap, consistent with what was reported previously for $MoOCl_2$ at visible and near-IR frequencies [s41467-024-53988-7].

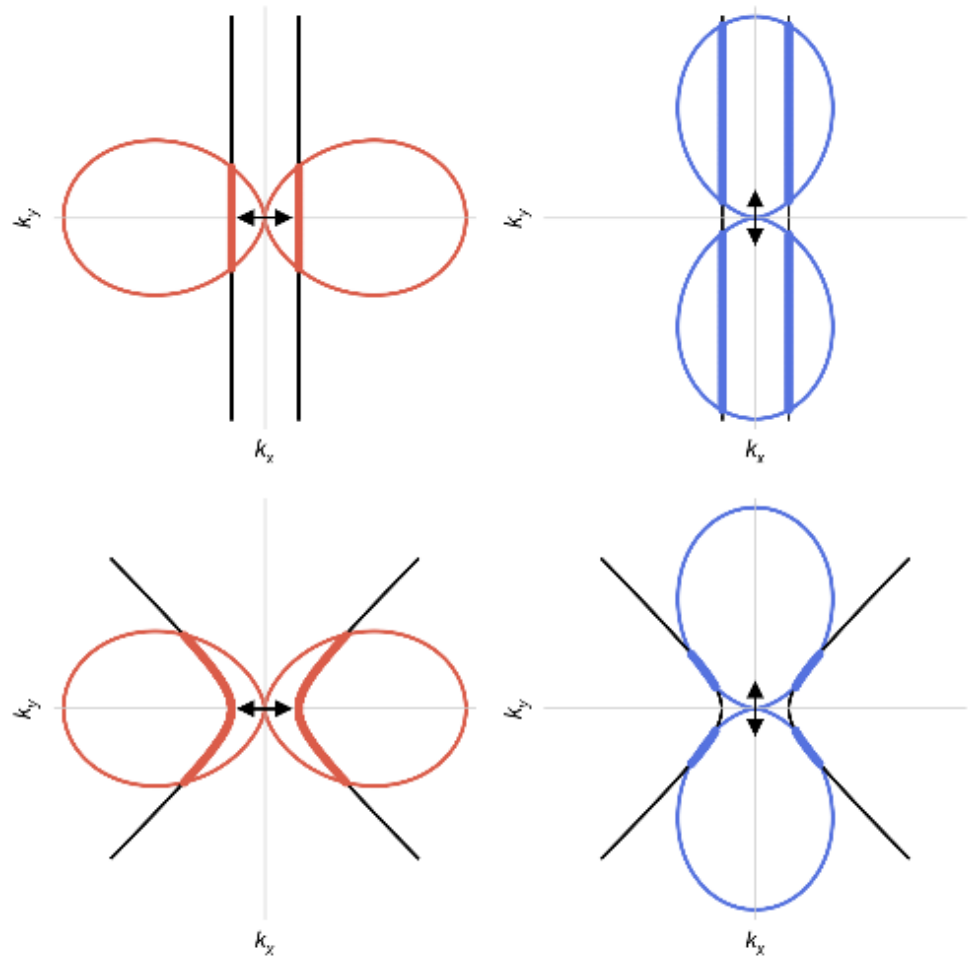

Figure 9S. **Modelling the rod direction as near-field dipole orientation**. Momentum-space overlap between dipole emission and polariton isofrequency contours. The angular distribution of an in-plane dipole is superimposed on representative Isofrequency contours for canalized (top) and hyperbolic (bottom) regimes. For canalization, where the Isofrequency contours are nearly vertical, a dipole oriented along [010] provides stronger overlap with the Isofrequency contours, leading to more efficient excitation of polaritons propagating along [100]. In contrast, for hyperbolic dispersion with Isofrequency contours opening along k_x, a dipole oriented along [100] exhibits improved overlap. Colored segments highlight the portions of the Isofrequency contours lying within the dipole emission distribution, indicating regions of enhanced coupling.

A more precise evaluation of the angular distribution of the dipole emission should involve the effect of the substrate on the dipole and thus the reflection part of the Greens function [doi10.1186/s43593-023-00047-1]. However, we believe that the simple case of a free-space dipole is enough for a qualitative understanding of the advantage of exciting the polaritons at the canalization wavelength with a plane wave polarized along the [010] direction rather than the [100] one.

Action taken:

We also performed again experiments with the different incident polarizations with both disks and rod, which now better show the different excitation efficiency. The Figure below (Fig. 10S) replaces the old Figure 8S:

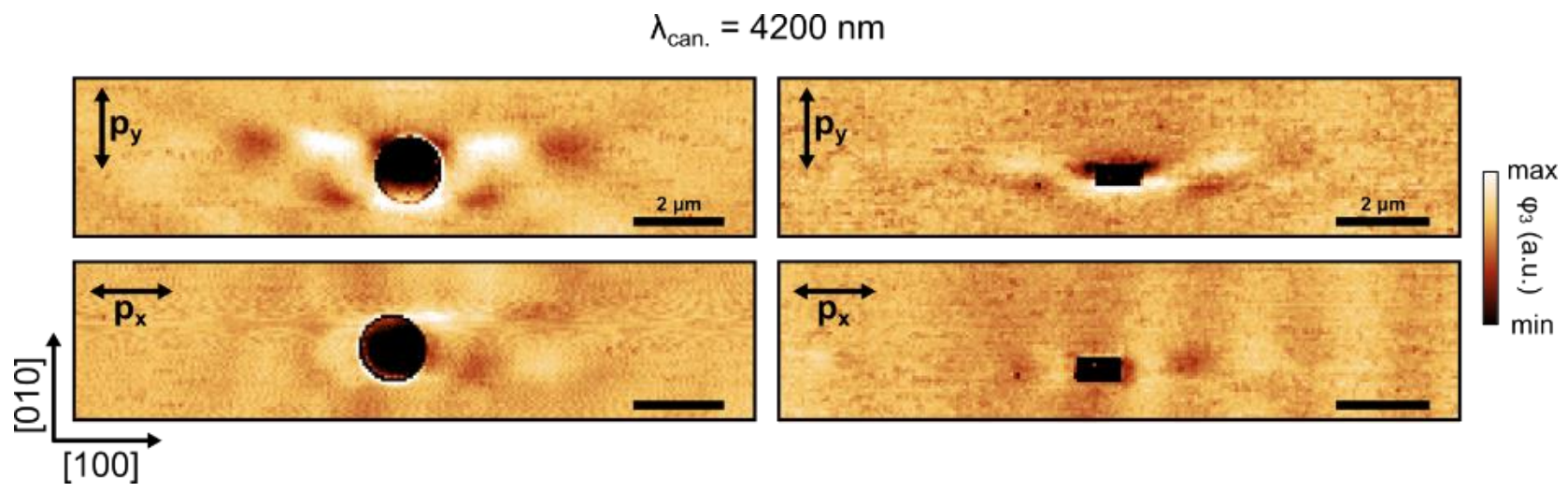

Figure 10S. Near-field phase maps for different incident polarization directions. sSNOM measurement performed at 4200 nm on a 45 nm-thick flake. A gold disk and a rod oriented along the [100] direction were used as plasmonic launchers (scale bars: 2 µm). In both cases, when the polarization is along the [010], more efficient polariton launching is observed.

A new Section 2 of the Supplementary Information has been added discussing in detail the effect of the incident polarization discussed above.

We also have slightly changed the part describing the polarization effect in the main text in relation to Figure 2:

In the experiments, the incident light is p-polarized to ensure efficient coupling to the s-SNOM tip. When the in-plane electric field component p_x is aligned with the metallic crystallographic axis [100], the launching efficiency of the gold nanoantenna is strongly suppressed, as illustrated in Fig. 2a. Corresponding simulations at λ_{can} = 4200 nm for a 45 nm-thick flake show rapidly decaying polaritons with only a few visible interference fringes. In contrast, aligning the in-plane polarization along the dielectric [010] axis significantly enhances the launching efficiency, resulting in more extended interference patterns (Fig. 2b). For this reason, all subsequent measurements were performed with the in-plane polarization aligned along [010]. This behavior can be qualitatively understood in terms of a better overlap between the angular distribution of the in-plane dipole induced in the launcher and the shape of the polariton isofrequency contours at the canalization condition (see Supplementary Information 2 for more details and experimental verification).

Our additional answer:

We agree with the reviewer that collinear illumination provides a complementary approach, as the resulting interference pattern contains contributions from both forward and backward propagating polaritons. In this sense, the modification of the fringe spacing can indeed be viewed as an additional source of information rather than a limitation. However, this comes at the cost of a more complex interpretation, as the measured periodicity includes contributions from both the polariton wavevector and the projection of the free-space wavevector, and therefore depends on the illumination angle. In practice, uncertainties in the exact excitation geometry can translate into uncertainties in the extracted polariton wavelength, particularly in the regime of modest confinement.

In the present work, our primary objective is the direct and quantitative extraction of the polariton wavelength and its evolution across the canalization condition. We therefore employ an excitation geometry that enables a more straightforward and robust analysis. At the same time, as discussed above, the choice of the present geometrical configuration is primarily determined by the higher launching efficiency discussed above. We have clarified this point in the revised manuscript and note that both configurations offer complementary advantages depending on the measurement goal.

Action taken: Regarding this point, we now write in the main text:

An additional advantage of this arrangement is that no correction of the isofrequency contours obtained from the experimental FFTs is required. This is because the light wavevector is nearly orthogonal to the polariton propagation direction, so the measured fringe periodicity directly corresponds to the polariton wavelength. In contrast, for collinear illumination geometries, the measured periodicity includes an

additional contribution from the projection of the free-space wavevector, which depends on the illumination angle [59, 60]. While this can be advantageous for accessing additional information by simultaneously observing both forward and backward-propagating polaritons with respect to the incident light momentum, it also introduces additional uncertainty in the quantitative extraction of the polariton wavevector due to the angle-dependent momentum contribution.

Reviewer #3:

2) The authors barely discuss propagation losses for these canalized modes. Overall, there is barely more than one fringe visible which would ask for – at least – a critical discussion of losses. Moreover, following the behavior of the permittivities, one would expect a trend for the propagation losses with wavelength. I would have guessed that with imaginary parts in both in-plane permittivity components going up with increasing wavelength, that also the propagation losses increase with wavelength. In fact, I would qualitatively even see this in Fig. 3b, where the quality of the 4.2 micron mode seems significantly better than the 6 micron measurement. I would kindly ask the authors to a) elaborate on propagation losses in general for their work

b) discuss expected or even observed changes in the losses for canalization wavelength tuning

Our answer:

We have added a new section of the SI dedicated to the discussion of losses and polariton propagation length. We now extract the propagation length from the experimental near-field maps by analysing line profiles taken along the polariton propagation direction and fitting them with a decaying oscillatory function. In this way we extract both the polariton wavevector and the propagation length. This analysis was performed for both disk and rod launchers as a function of wavelength, as well as for different flake thicknesses near the canalization condition. The extracted trends confirm that the propagation length is on the order of few microns, consistent with the modest number of visible fringes in the near-field images.

Regarding the wavelength dependence, our measurements show that the propagation length generally decreases as the polariton momentum (normalized to free-space) increases. This is consistent with the expected trade-off between confinement and loss: modes with larger normalized wavevector have stronger field confinement associated with stronger field penetration in the material and thus losses, leading to shorter propagation distances. In the thickness-dependent measurements just below the canalization wavelength, we find that thinner flakes, in which the isofrequency contours lie at larger polariton momenta, show reduced propagation lengths. This is, we believe, the main underlying reason for the observation of reduced propagation length for the redshifted canalization wavelengths, and not the increase of the increased imaginary part of $Im(\varepsilon_y)$. Going to longer wavelengths in an isotropic Drude material (i.e. an isotropic metal) generally leads to lower losses even tough $Im(\varepsilon)$ is increasing, because the real part is also becoming more negative, leading to lower field penetration and smaller overall losses.

Action taken: The new section of the SI reads (section 7):

Propagation length of anisotropic plasmon polaritons

To quantify the propagation length of mid-IR polaritons in $MoOCl_2$, we extract decay profiles along the propagation direction for the measurements taken on the 45 nm flake at different wavelengths. We investigate launching from both a disk antenna (Fig. 25S) and a rod antenna (Fig 26S).

In panel a of each figure, we report the near-field s-SNOM phase maps at different wavelengths. The white arrow indicates the direction along which the polariton profiles are extracted. Panel b depicts the corresponding line profiles (offset for clarity), together with fits (black lines) using a decaying

exponential function $f(x) = exp(-\alpha x)cos(kx + \phi) + C$. From these fits, we extract both the polariton wavevector k and the propagation length $L_p = 1/\alpha$.

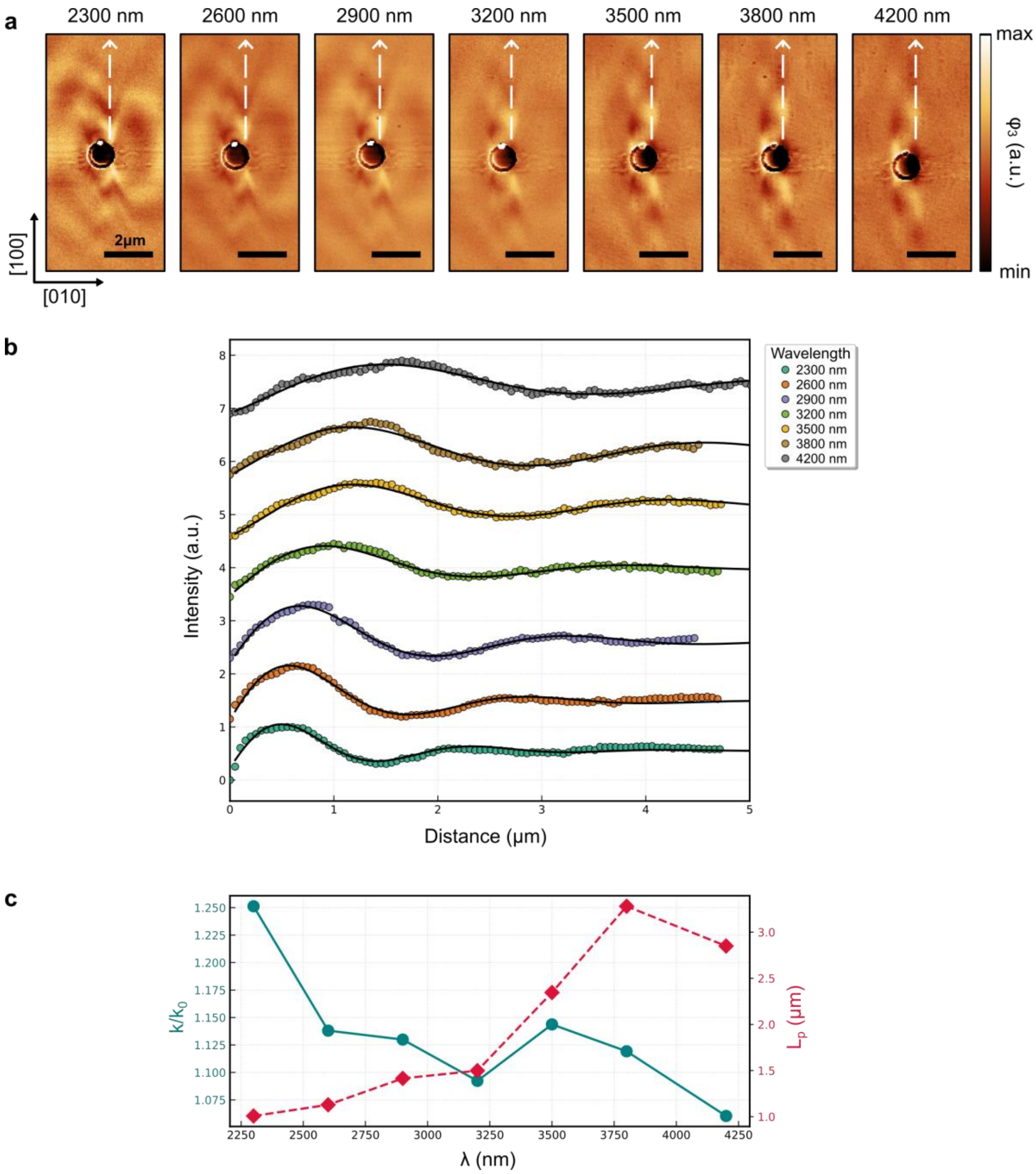

Figure 25S. Polariton propagation length on a 45 nm flake launched by a disk antenna. a sSNOM phase maps from which decay profiles are extracted along the white dashed lines. **b** Extracted profiles fitted with an exponentially decaying function. Curves are offset for clarity. **c** Retrieved wavevector and propagation length as a function of wavelength.

The results are summarized in panel c, where we plot the extracted wavevector normalized to the free-space value, k/k_0, and the propagation length as a function of wavelength. We observe a decrease in the polariton wavevector accompanied by an increase in the propagation length as the wavelength increases.

This trend is expected, as higher confinement (larger k/k_0) is generally associated with stronger losses and thus shorter propagation distances. The analysis is performed up to wavelengths until the

canalization condition, as beyond this wavelength polaritons become strongly damped, making it difficult to reliably extract a propagation length from the experimental data.

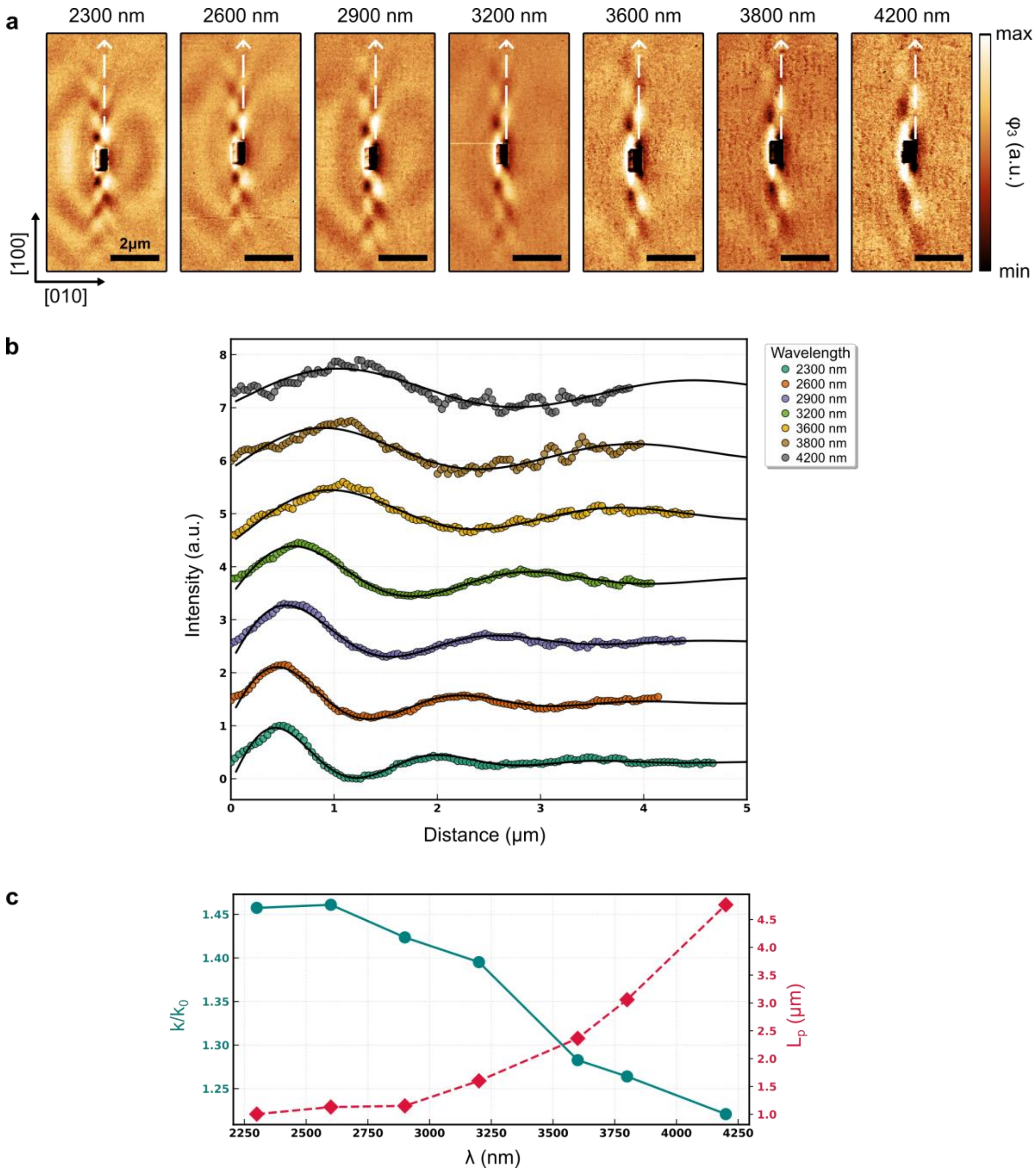

Figure 26S. **Polariton propagation length on a 45 nm flake launched by a rod antenna. a** sSNOM phase maps from which decay profiles are extracted along the white dashed lines. **b** Extracted profiles fitted with an exponentially decaying function. Curves are offset for clarity. **c** Retrieved wavevector and propagation length as a function of wavelength.

The increased damping observed near the canalization frequency can be attributed to the vanishing of the ε_y component, which leads to enhanced electric fields in the material. As a result, even moderate intrinsic losses are effectively amplified, yielding a reduction of the propagation length in the vicinity of the canalization condition.

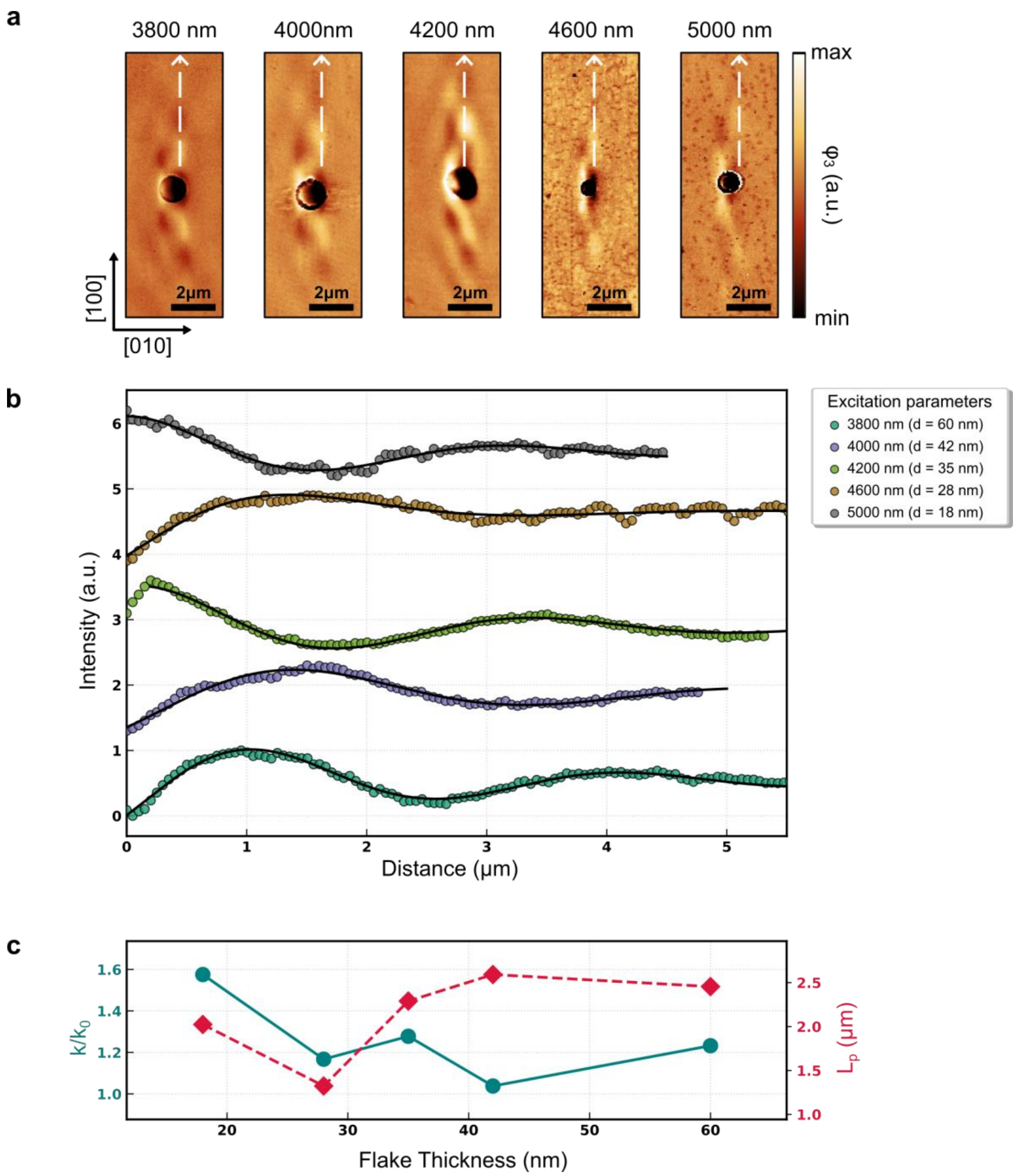

Figure 27S. **Polariton propagation length at different film thicknesses. a** sSNOM phase maps from which decay profiles are extracted along the white dashed lines. Each map is taken at a different wavelength (just before the canalization condition) and corresponds to a different flake thickness (in order of decreasing thickness, with same values as in Fig. 12S. **b** Extracted profiles fitted with an exponentially decaying function. Curves are offset for clarity. **c** Retrieved wavevector and propagation length for the different flake thicknesses (and thus different canalization frequencies).

To further investigate the evolution of the polariton propagation, we performed a thickness-dependent analysis of the polariton propagation length at wavelengths just below the canalization condition where it is easier to extract the propagation length (Fig. 27S). We observe that decreasing the flake thickness (thus redshifting the canalization frequency) is accompanied by an increase in the polariton momentum and a corresponding decrease in the propagation length. This behavior is consistent with the fact that achieving canalization at longer wavelengths requires thinner flakes, which results in stronger confinement of the polariton modes. As expected, increased confinement leads to enhanced losses and therefore shorter propagation distances.

Reviewer #3:

3) In Fig. 3e, the authors show numerical results for the thickness dependence of the topological transition. I was surprised to find in the SI, Fig. 7Sc, a very intuitive plot that would actually (potentially) explain the tuning mechanism, but which is not mentioned in the main text. The argument/observation that canalization emerges at a constant value of d*eps[100] is actually very interesting, and should be discussed in the main text.

Our answer:

We thank the reviewer for this helpful suggestion. While revising the argument for the canalization frequency shift with flake thickness we realized our argument was too unpolished and did not stand further inquiry. We revise here the principle underlying the topological transition shift. First of all, we can consider the simplest case: a material with constant dielectric function ($\varepsilon_x = -500 + 100i;\ \varepsilon_y = -2 + 0.1i$) showing canalization at 5500 nm at a thickness of 35 nm. In this section we restrict ourself to the case of a layer in vacuum to avoid any effect that could be related to the substrate refractive index. The evolution of the isofrequency contours calculated from the transfer matrix is shown in **Figure R1**. A transition from hyperbolic (at low thickness) to elliptic (high thickness) can be seen **even with a constant dielectric function**. This necessarily means that the frequency shift is not driven only by a dispersive dielectric function, but also by the relative change between the free-space momentum and the flake

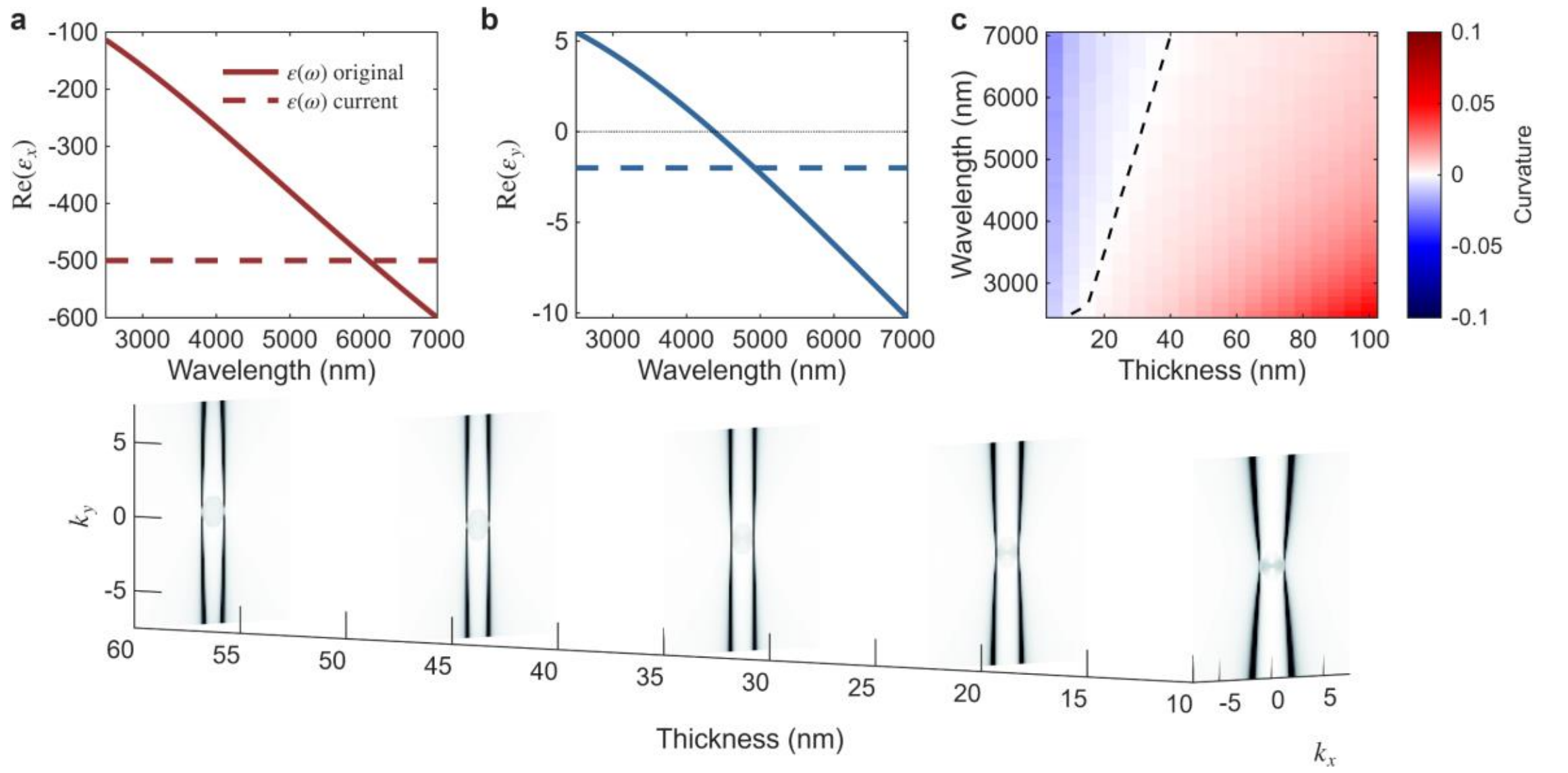

Figure R1. Canalization shift for a material with constant dielectric function (dashed lines, $\varepsilon_x = -500 + 100i;\ \varepsilon_y = -2 + 0.1i$). The used permittivity is shown along the MoOCl2 mid IR dielectric function along the **a** x and **b** y directions. **c** Shift of the canalization frequency computed from the analytical dispersion as inferred from the zero of the isofrequency contour vertex curvature. **d** Isofrequency contours calculated from the transfer matrix at 5500 nm for flakes of different thicknesses.

thickness (here fixed). Indeed, by looking at eq. 8 of the SI (dispersion for film much thinner than wavelength), the thickness always appears multiplied by k_0 in all terms. This means that, regardless of the specific isofrequency contours shape, changing the thickness by a factor xd can be compensated by varying at the same time the free space momentum k_0/x. This basically means that an **increase** in thickness leads to an **increase** in the canalization frequency in a linear way. This is opposite to what we observe in the experiments and simulations, meaning that the Drude dispersion must overcompensate this effect to produce the observed behaviour (**increased** thickness leads to a **decreased** canalization frequency in the experiments). As the terms containing the dielectric function are of the type $d\varepsilon(\lambda_0)/\lambda_0$, it is not surprising that a dielectric function that has a decreasing value (negative, so increasing absolute value) at longer wavelengths can compensate the trend from λ_0. From the analytical calculation of the

isofrequency contours evolution, we can confirm that indeed a constant dielectric function leads to a linear shift of the canalization wavelength with flake thickness.

To better understand the role played by the dispersive nature of the permittivity along the two different directions, we can analyse what happens when one axis is kept constant while the other becomes dispersive. We first keep the y direction to be $\varepsilon_y = -2 + 0.1i$, while we use the permittivity of $MoOCl_2$ for the x-direction (**Figure R2**). The resulting isofrequency contours are almost unchanged compared to the previous case of constant dielectric function, indicating that the dispersion along the x-direction does not influence much the shift of the canalization frequency. Tracking the shift of the canalization wavelength through the analytical isofrequency contours also shows that the trend is very similar to the one of constant permittivity as the canalization wavelength scales linearly with the flake thickness. The small role played by ε_x in this range is to be expected as the permittivity is very negative and the material closely resembles a perfect electric conductor. Thus, the dispersion along x is not the one responsible for the canalization frequency shift.

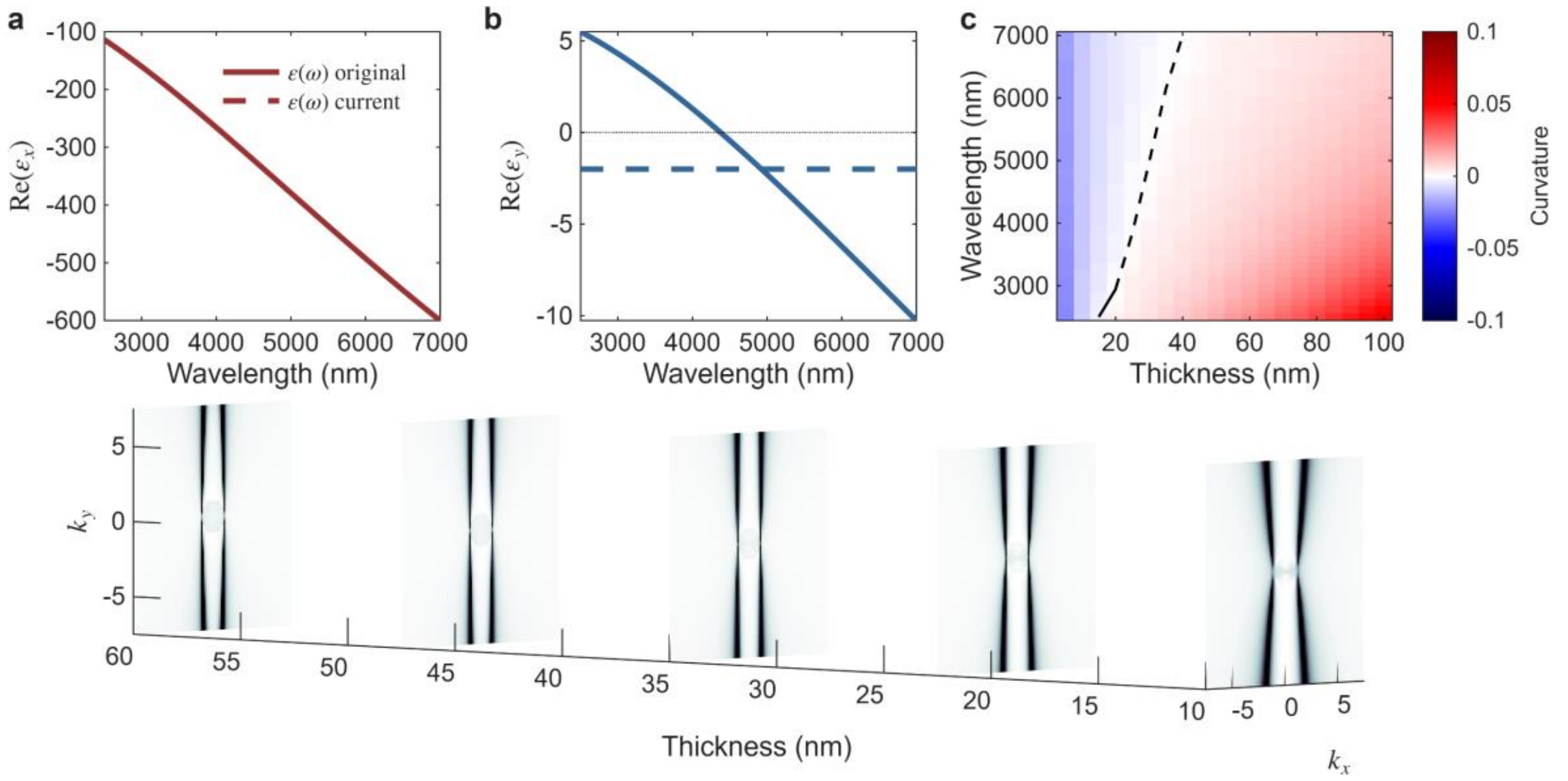

Figure R2. Canalization shift for a material with the mid-IR dielectric function of MoOCl2 along x and a constant permittivity along y (dashed lines $\varepsilon_y = -2 + 0.1i$). The used permittivity is shown along the MoOCl2 mid IR dielectric function along the **a** x and **b** y directions. **c** Shift of the canalization frequency computed from the analytical dispersion as inferred from the zero of the isofrequency contour vertex curvature. **d** Isofrequency contours calculated from the transfer matrix at 5500 nm for flakes of different thicknesses.

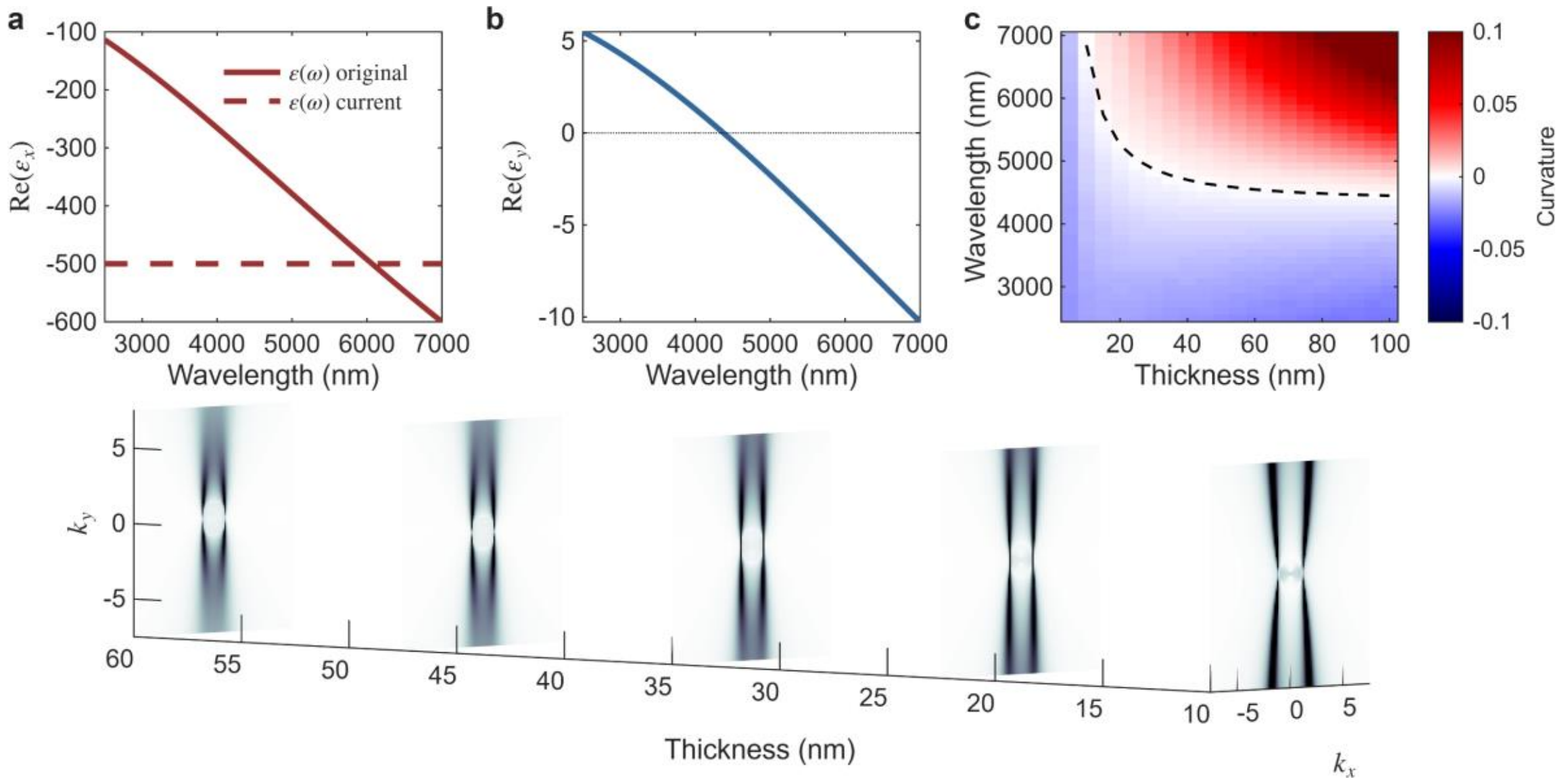

Figure R3. Canalization shift for a material with the mid-IR dielectric function of MoOCl2 along y and a constant permittivity along x (dashed lines $\varepsilon_x = -500 + 100i$). The used permittivity is shown along the MoOCl2 mid IR dielectric function along the **a** x and **b** y directions. **c** Shift of the canalization frequency computed from the analytical dispersion as inferred from the zero of the isofrequency contour vertex curvature. **d** Isofrequency contours calculated from the transfer matrix at 5500 nm for flakes of different thicknesses.

At the same time, we can investigate the opposite case, where $\varepsilon_x = -500 + 100i$ is kept constant while the permittivity along y is the one of $MoOCl_2$ in the mid-IR (**Figure R3**). The isofrequency contours show a much quicker transition towards the elliptical regime at high flake thicknesses, signalling a different qualitative trend compared to the previous ones. Indeed, tracking the calculated shift of the dielectric function yields results that are very similar to the one experimentally obtained. This observation confirms that the shift of the canalization wavelength is mainly driven by the dispersion along the y direction, while the dispersion in x plays a secondary role with only a small quantitative difference from the constant case.

Action taken: **Section 5** (previous section 4) of the SI has been completely reworked to include this analysis of the canalization wavelength shift origin and how it is related to the material in-plane dielectric function.

For the sake of brevity, in the Abstract we simply mention that the slope of the Drude term is what allows broadband canalization and thickness-tuning:

Owing to the moderate slope of the Drude permittivity, the resulting polaritons remain highly directional across a broad spectral window. This weak dispersion also enables robust thickness-dependent tuning, and we demonstrate, both experimentally and theoretically, that the canalization wavelength can be adjusted by more than 1 μm simply by varying the flake thickness.

At the end of the introduction we now added:

In contrast to conventional phonon-polaritonic platforms governed by Lorentz-type dispersions, the Drude-like response of $MoOCl_2$ enables broadband canalization over an extended spectral range while simultaneously providing efficient thickness-dependent tunability. The observed shift of the canalization wavelength is primarily driven by the dispersion along the [010] axis, whereas the strongly metallic response along [100] remains deeply negative and therefore only weakly influences the evolution of the isofrequency contours curvature with thickness.

In the description of Fig. 3 of the main text we now write:

By looking at the equation for the theoretical isofrequency contours (Supplementary Information 5), additional insight into the thickness dependence of the canalization condition can be obtained. Assuming a frequency-independent dielectric response, with one in-plane permittivity component remaining deeply negative and the other negative but closer to zero, the canalization wavelength is expected to increase linearly with thickness. This follows from the analytical expression, where the slab thickness and free-space wavevector appear only through their product, so that the canalization condition is preserved by scaling thickness and wavelength by the same factor (see Supplementary Information 5). In $MoOCl_2$, however, the opposite trend is observed experimentally and theoretically: increasing the flake thickness leads to a reduction of the canalization wavelength. This behavior is mainly driven by the dispersion of the [010] permittivity, which governs the evolution of the isofrequency contours shape across the topological transition. Since the permittivity enters the canalization condition multiplied by the flake thickness d, an increase in the absolute value of ε_y (i.e., a more negative response) requires a smaller thickness to satisfy the condition. As a result, the thickness dependence of the canalization wavelength qualitatively follows the Drude-like dispersion of the [010] axis, explaining the trend reported in Fig. 3e. In contrast, the permittivity along the [100] direction remains strongly negative throughout the relevant spectral range and therefore behaves almost as a perfect electric conductor, contributing only weakly to the thickness-induced shift of the canalization condition.

Reviewer #3:

In particular, it also means that the zero-crossing or "near-zero" behavior of the [010] permittivity is almost irrelevant in the given regime since the "other" direction seems to dominate the behavior. Is it solely a coincidence the [010] permittivity happens to also cross zero in this spectral region? Would the concept also work with a small-index dielectric (and non-dispersive) behavior along the [010] direction? I would love to see a more in-depth discussion of these questions in the revised manuscript, since this would allow to also understand the experiments in a more general way, beyond simply observing something "cool".

Our answer:

As discussed above, in revisiting the physical origin of the thickness-dependent canalization shift, we realized that our previous explanation was incomplete. The zero-crossing of the [010] permittivity is essential for obtaining the canalization condition. While the strongly negative [100] response controls the metallic character and confinement of the modes, the transition from hyperbolic to elliptical isofrequency contours is mainly governed by the dispersion of the [010] permittivity.

To clarify this point and analyse the influence of the ε_y value, we performed additional calculations where $\varepsilon_x(\omega)$ was kept equal to the fitted $MoOCl_2$ permittivity, while $\varepsilon_y$was fixed to selected non-dispersive values around 0 $(-2, -1, -0.5, 0)$. This allows us to isolate the role of the [010] permittivity and directly test whether canalization can be obtained with a fixed small-index dielectric response (Fig. 18S). These simulations show that the canalization-thickness relation keeps being linear at different negative values of ε_y, with values closer to zero pushing the canalization to higher thickness and wavelengths. Moreover, we observe that when the [010] permittivity becomes positive the transition to the elliptical regime is lost, and the canalization condition is only approached asymptotically as very flat hyperbolas. The same trend is observed with a larger range of thicknesses (last panel), where the curvature is small and negative for a broad range of wavelength and thicknesses, but never becomes positive. We would remark that this analysis is done in the small thickness limit, and as such is expected to break down at some point, where the out of plane component of the dielectric function ε_z also starts to play a role, making the canalization condition more complicated to study.

Action taken: In Section 5 of the SI we added the following:

To assess the role of the [010] permittivity in setting the canalization condition, we calculated the isofrequency contours curvature evolution while keeping $\varepsilon_x(\omega)$ equal to the fitted MoOCl2 response and fixing ε_y to selected non-dispersive values around zero (Fig. 18S). This allows us to isolate the effect of the [010] dielectric response independently of its dispersion. For negative values of ε_y, the calculated zero-curvature line remains linear as a function of thickness, with values closer to zero shifting the canalization condition toward larger thicknesses and longer wavelengths. In contrast, when ε_y becomes positive (here we analyse the $\mathrm{Re}(\varepsilon_y) = 0$, as the same happens for small positive values of $\mathrm{Re}(\varepsilon_y)$), the isofrequency contours remain hyperbolic within the thin film approximation and the curvature approaches zero only asymptotically, without crossing into the elliptical regime. This behavior is also observed when extending the calculation to a broader thickness range, where the curvature remains small and negative over a wide spectral region (last panel of Fig. 18S). These results show that a fixed small positive dielectric response along [010] is not sufficient to obtain a true hyperbolic-to-elliptical topological transition. We note that this analysis is performed within the thin-film approximation. At larger thicknesses, this approximation is expected to gradually break down, and the out-of-plane response ε_z also plays a role in determining the isofrequency contours shape. In this regime, the canalization condition is no longer governed solely by the in-plane components ε_x and ε_y, and the topological transition depends on the full biaxial dielectric tensor and its analysis more complicated.

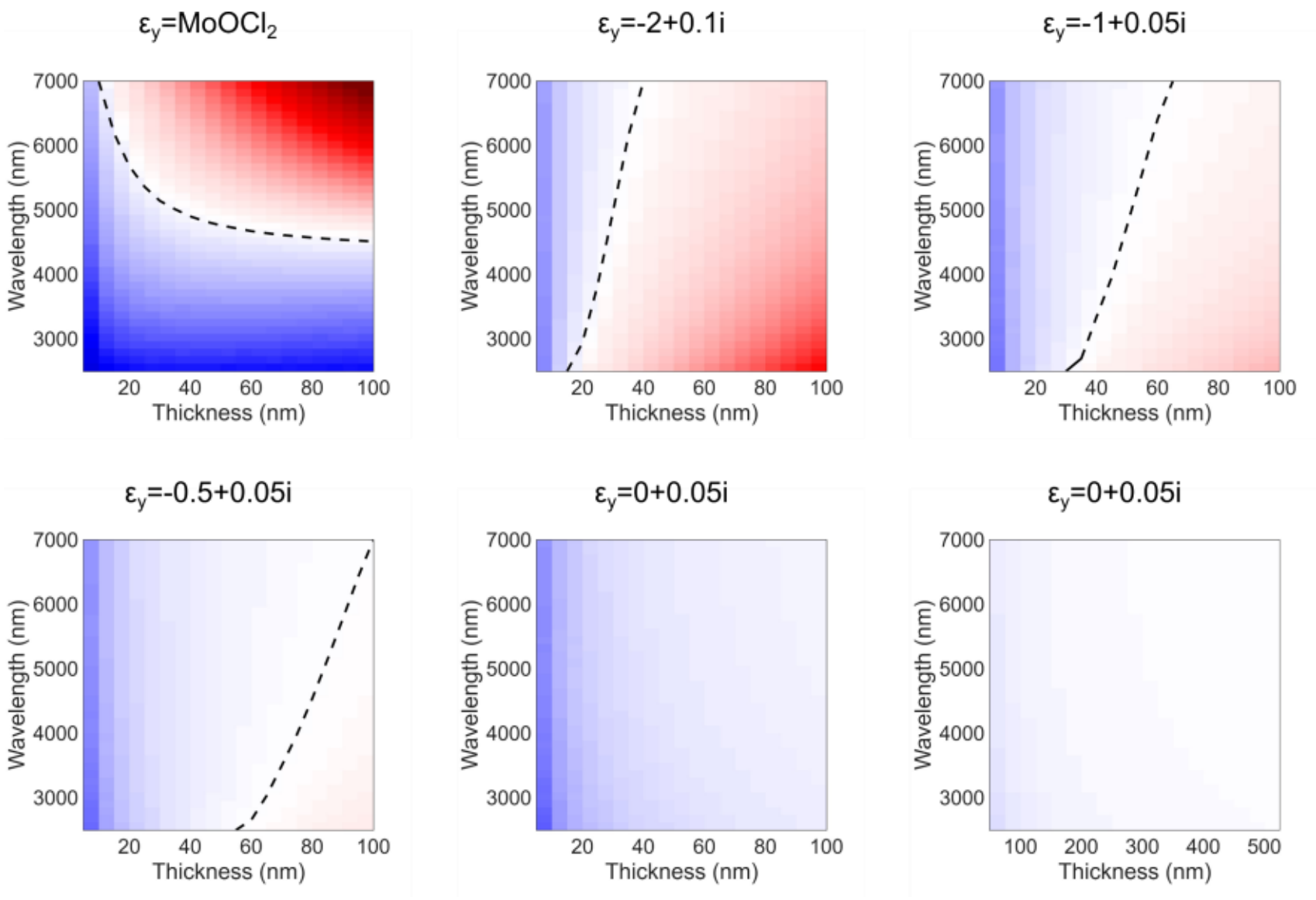

Figure 18S. Canalization shift for materials with the mid-IR dielectric function of MoOCl2 along x and a near-zero constant permittivity along y. Calculated Isofrequency contours curvature in the small-thickness limit for different constant values of the [010] permittivity. Dashed black lines indicates the canalization condition.

Our additional answer:

In addition, as discussed in our response to Reviewer 2, we analysed a model Lorentz–Lorentz material response representative of phonon-polariton van der Waals systems. In this case, despite the presence of a natural topological transition, the canalization wavelength shows only weak thickness dependence. This further supports the conclusion that the broad thickness tunability observed in $MoOCl_2$ is tied to the Drude-like dispersive response of the material.

Reviewer #3:

4) The canalized modes observed here overall have a rather limited level of confinement, likely related to the rather large negative permittivity along [100]. Could the authors sketch any credible scenario on how to further compress the canalized modes?

Our answer:

We agree with the reviewer that the moderate confinement of the canalized modes is mainly linked to the large negative value of the in-plane permittivity along the [100] direction. A simple way of increasing the confinement is to reduce the film thickness which generally pushes the isofrequency contours to higher momenta. Alternatively, the substrate can be engineered: replacing the present dielectric substrate with a metallic substrate, such as gold, would modify the symmetry and boundary conditions of the slab modes. In a metal-backed geometry, the lowest-order mode can be suppressed and canalization could be achieved with a higher-order mode. Such higher-momentum modes are intrinsically more confined, since their dispersion lies at larger in-plane wave vector.

Action taken: We confirm that higher confinement can be achieved in these geometries by simulations, and summarize these aspects in the following SI section:

The moderate confinement of the canalized modes observed in the present geometry is mainly caused by the large negative permittivity along the [100] direction, which shifts the canalized isofrequency contours toward relatively small in-plane momenta. To evaluate possible routes toward stronger compression, we analyze the confinement of the isofrequency contours as a function of film thickness and wavelength. From the conic fit of the analytical isofrequency contours, the value of the in-plane

momentum at the $k_y = 0$ intercept can be extracted. Since the isofrequency contours are nearly straight at the canalization wavelength, this value also represents the characteristic momentum of the whole canalized mode. Fig. 28S shows the extracted k_x ($k_y = 0$) as a function of film thickness and wavelength. The white dashed line marks the canalization wavelength. The corresponding confinement factor extracted along this line is also shown. The result demonstrates that thinner flakes shift the canalized response toward larger in-plane momentum, and therefore provide a direct route to stronger modal compression.

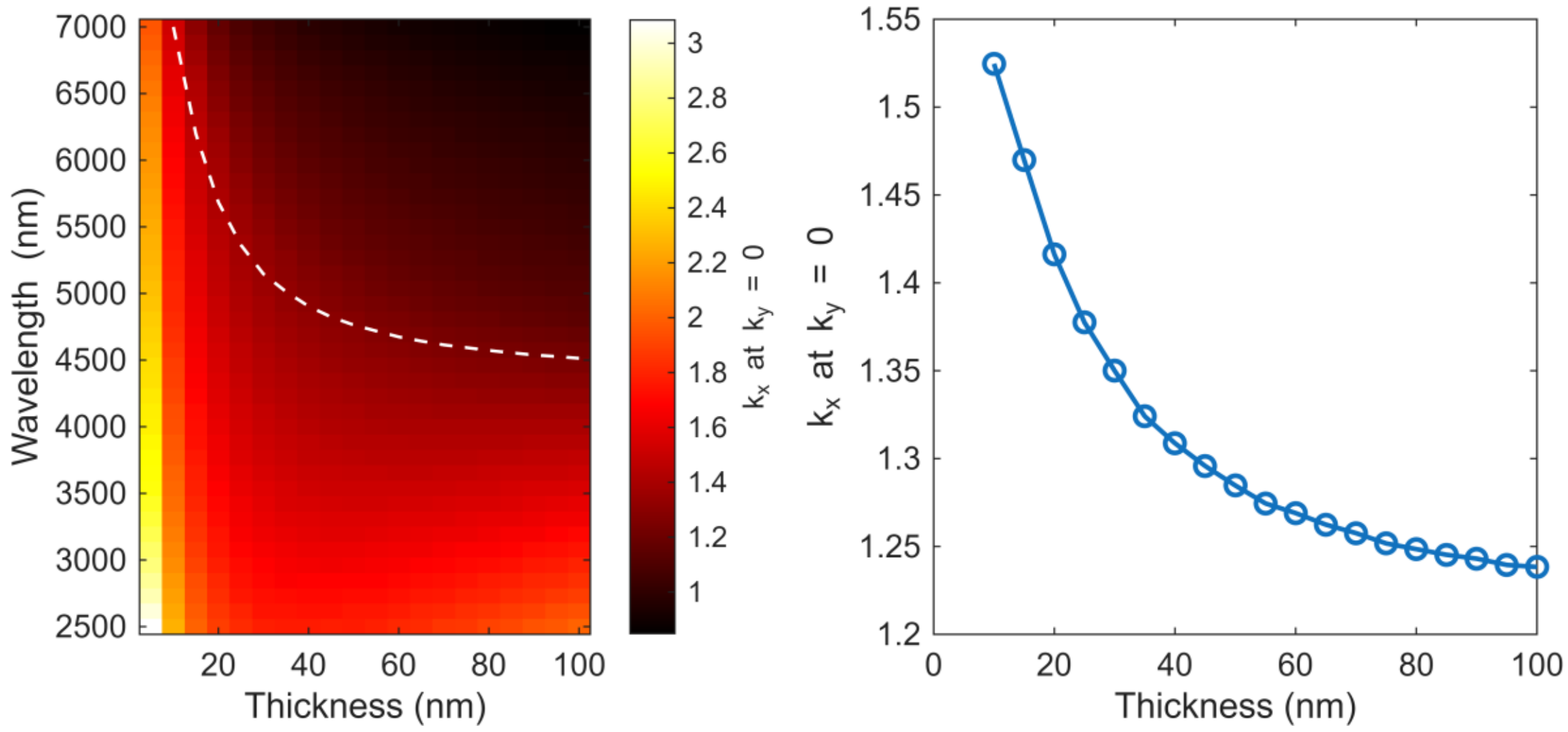

Figure 28S. Polariton confinement as a function of flake thickness. Map of the extracted in-plane momentum $k_x(k_y = 0)$ as a function of film thickness and wavelength (left). The white dashed line indicates the canalization wavelength extracted for each thickness. Confinement factor extracted along the canalization line (right).

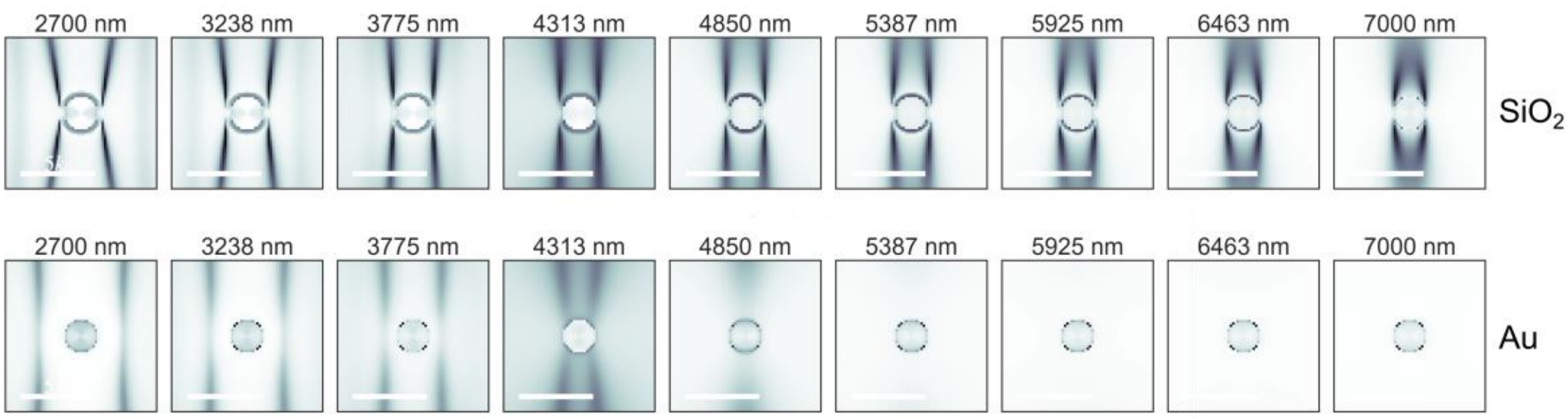

Figure 29S. Polariton dispersion on SiO2 and Au substrates. Two-dimensional transfer-matrix calculations of the polaritonic response for a MoOCl2 film placed on a SiO2 substrate (top) and an Au substrate (bot).

A second route for enhanced confinement is substrate engineering. Fig. 29S compares two-dimensional transfer-matrix calculations for the same film placed on a SiO2 substrate and on an Au substrate. In the SiO2-supported geometry, the lowest-order mode dominates the response and canalization occurs at relatively small momentum. In contrast, for the Au-supported geometry, the metallic boundary modifies the modal symmetry and suppresses the first mode through symmetry and screening at the metal interface [11]. Canalization can therefore occur on a higher order branch located at larger in-plane momenta, leading to stronger confinement.

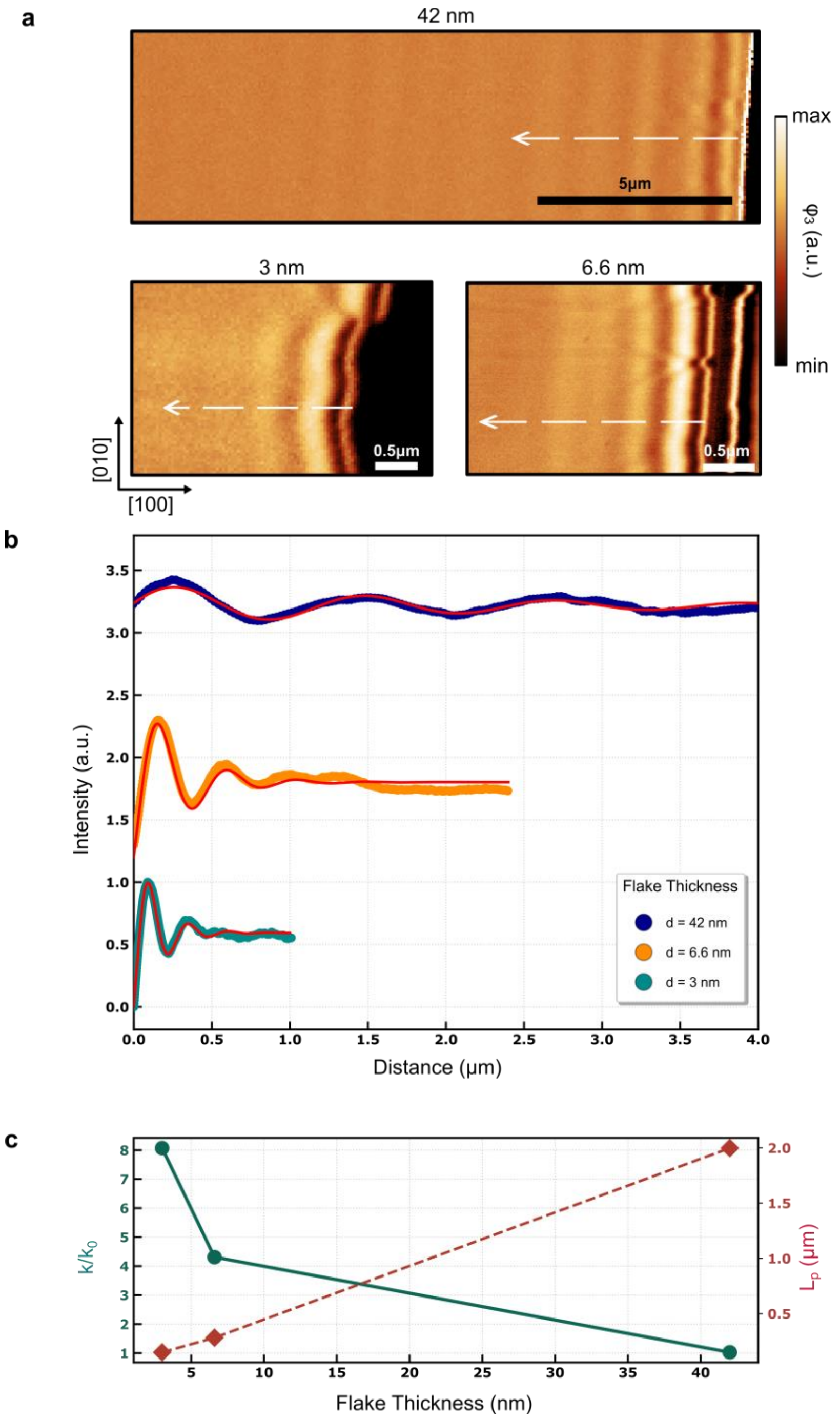

Figure 30S. **Polariton propagation length at different film thicknesses. a** sSNOM phase maps from which decay profiles are extracted along the white dashed lines. Each map is taken at the same wavelength 2300 nm and corresponds to a different flake thickness. **b** Extracted profiles fitted with an exponentially decaying function. Curves are offset for clarity. **c** Retrieved wavevector and propagation length for the different flake thicknesses.

Furthermore, we performed measurements at a fixed wavelength of 2300 nm on ultrathin films with thicknesses of 3 nm and 6.6 nm to demonstrate that SPP modes can still be sustained in this extreme thickness regime. The results were compared with those obtained for a 42 nm-thick film, and the extracted polariton momentum and propagation length as functions of flake thickness are summarized in Fig. 30S. As the film thickness decreases to 3 nm, the plasmon confinement increases dramatically, reaching values up to eight times larger than those observed in the thicker films, albeit at the expense of reduced propagation length. We also confirm (Fig. 31S) that the experimental momenta for the two

thinner flakes are compatible with being tip-launched polaritons ($k_{exp} = 2k_{SPP}$ [12]) along the [100] direction reflected by the flake edge.

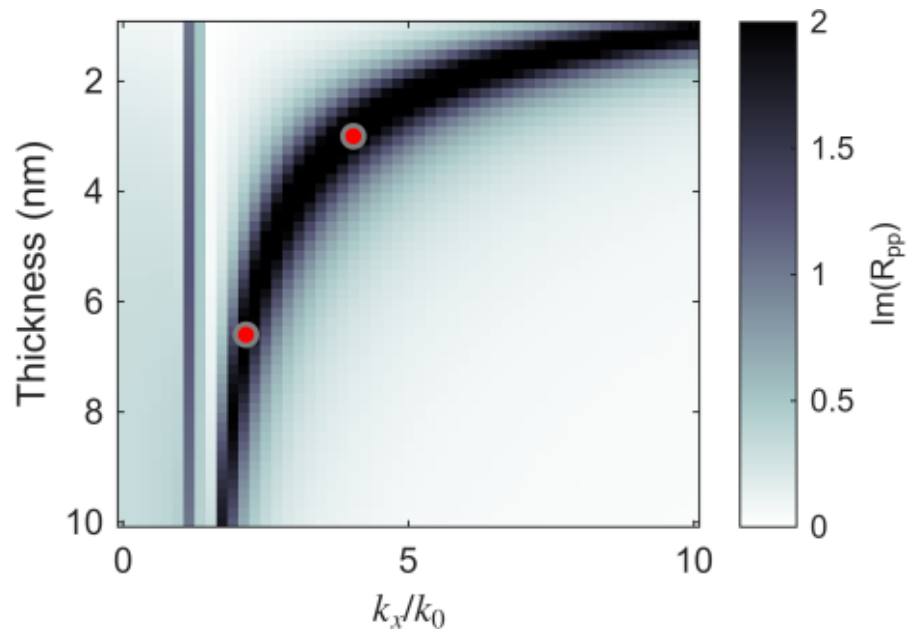

Figure 31S. Comparison of experimental momenta with transfer-matrix theory. Experimental tip-launched momenta ($k_{exp} = 2k_{SPP}$) from the data in Fig. 30S overlayed on transfer matrix calculations for MoOCl2 SPPs along the k_x direction at λ = 2300 nm.

Reviewer #3:

A few minor issues:

• Ref. 5 is not studying a vdW system as suggested in the place where it is cited. A more fitting reference specifically for that sentence could be: Kowalski et al., Nat. Mater. 24, 1735 (2025).

Our answer/action taken:

We thank the reviewer for spotting this inconsistency in Ref. 5. The reference has therefore been replaced with the one suggested by the referee.

Reviewer #3:

• Ref. 41. This is an interesting work, but I think the original study for substrate-induced topological transitions is more appropriate, or should at least be also cited: Duan et al., Sci. Adv. 7, eabf2690(2021).

Our answer:

We thank the reviewer for pointing out this relevant work. We agree that Duan et al., Sci. Adv. 7, eabf2690 (2021), provides an important earlier demonstration of substrate-induced topological transitions in polariton dispersion, and we have added it to the revised manuscript. We note, however, that in that work the measurements were performed at higher energies (compared with old Ref. 41) within the Reststrahlen band and did not focus on the canalization condition. We therefore cite it as an important precedent for substrate-induced topological transitions, while also maintaining Ref. 41 for the specific discussion of polariton canalization.

Reviewer #3:

• Considering that MoOCl2 is a monoclinic crystal, it would be appropriate to also cite the first work (before ref. 48) on highly directional shear polaritons in a monoclinic system in the introduction, amongst what is now refs. 10-18: Passler et al., Nature 602, 595 (2022).

Our answer:

We agree and have added the suggested reference in the introduction.

Reviewer #3:

• The statement "[…], losses due [to] overlapping phonon resonances, […]" on page 3 is slightly misleading in my view. For one, the losses in most plasmonic systems are much greater than in most phononic systems. But more importantly, the authors use a Drude-Lorentz oscillator model to describe the material, so also here there is a phenomenological Drude oscillator involved. I believe, in particular considering the actual losses involved in the current work, this statement should be reframed/changed. It is really not a good one.

Our answer:

We agree with the reviewer that the original phrasing could be misleading. Our intention was not to suggest that phonon-polariton systems are intrinsically more lossy than plasmonic ones, but rather to highlight that, in many experimentally studied phonon-polariton materials, the frequency of the natural topological transition often coincides with spectral regions of increased loss due to nearby transverse optical (TO) phonon (leading to peaks in the imaginary part of the permittivity).

While there is no fundamental reason for this to occur, it appears to be a recurring feature in several materials. For example, in MoO_3 the first sign change of the in-plane permittivity occurs close to the TO phonon of orthogonal component, leading to strongly damped polaritons at the canalization frequency, as also observed experimentally [10.1364/OPTICA.547698]. A similar situation is found in LiV_2O_5 [s41467-024-46935-z], rare-earth oxyorthosilicates [10.1038/s41467-024-50939-0], and V_2O_5 [s41563-020-0665-0]. In V_2O_5, a recent work [10.48550/arXiv.2604.12174] reports canalization in a regime that does not correspond to a full hyperbolic-to-elliptical transition, but rather to a transition toward suppressed propagation where both in-plane components of the permittivity are positive, again leading to weak propagation.

There are exceptions to this trend. In gypsum [10.1126/sciadv.adw3452], the transition occurs far from TO peaks, although propagation is limited by the weak phonons oscillator strength. In YVO_4 canalization has been observed far from TO phonons, albeit in a very narrow spectral window and requiring low-temperature operation.

Overall, our point is that, due to the specific spectral structure of the dielectric function in many phonon-polariton materials, the natural topological transition frequently occurs in regions where polariton propagation is strongly damped, making the observation of canalization challenging. In contrast, in $MoOCl_2$ the topological transition occurs far from any resonance (the Lorentz contributions in the [100] direction lie at significantly higher energies, and we actually describe the material without it now, while in the [010] one the Lorentz contribution is only a small perturbation over the Drude response), enabling the clear observation of naturally canalized plasmon polaritons at room temperature.

Action taken: We have revised the corresponding sentence in the manuscript introduction to better reflect this point:

A conceptually simpler approach to achieve canalization is to exploit the natural hyperbolic to elliptical transition occurring when one component of the permittivity changes sign. However, despite its simplicity, this effect has thus far been mainly explored in phonon-polariton platforms, where its observation is often hindered by the spectral structure of the dielectric response. In many cases, the transition frequency lies close to a phonon resonance, leading to increased damping and limiting the visibility of propagating canalized modes [45-50], in addition to the intrinsically narrow bandwidth of Reststrahlen bands and, in some systems, the need for out-of-plane response [51] or low-temperature operation [52].

Reviewer #3:

• Conclusion, page 12, "simple yet powerful", I would ask the authors to refrain from bolstering statements like this. How "powerful" or not the current work and concept will be remains to be seen. I am not questioning the work, only that specific piece of language.

Action taken: We removed the "powerful" adjective in the conclusion.

Reviewer #3:

• Ref. 21 appears to be incorrect. It should read: Nature Nanotechnology 21, 23 (2026).

Action taken: The reference mentioned by the referee has been corrected.

Additional Changes:

1) We realized the methods section for the sample preparation was incomplete, missing details on the launcher fabrication process. We now added:

   Flakes with suitable ranges of thicknesses were selected for the launcher fabrication via the flake colour. The thicknesses were then confirmed by AFM measurements. The nanostructures were fabricated by a standard electron-beam lithography followed by metal deposition and lift-off. A layer of poly(methyl methacrylate) resist, PMMA 950K A4, was spin-coated onto the substrate at 4000 rpm for 60 s and subsequently baked at 180 °C for 3 min. The resist was patterned using 50 kV electron-beam lithography with a 50 µm aperture. After exposure, the sample was developed for 60 s in a 1:3 mixture of methyl isobutyl ketone and isopropyl alcohol. The development was stopped by rinsing the sample in isopropyl alcohol, followed by drying under a nitrogen gas flow. A 2 nm chromium adhesion layer and a 45 nm gold layer were then deposited by electron-beam evaporation. Finally, lift-off was performed by immersing the sample in acetone for more than 3 h, leaving the patterned gold nanostructures on the flakes.
